# Visualization of Transient Protein-Protein Interactions that Promote or Inhibit Amyloid Assembly

**DOI:** 10.1016/j.molcel.2014.05.026

**Published:** 2014-07-17

**Authors:** Theodoros K. Karamanos, Arnout P. Kalverda, Gary S. Thompson, Sheena E. Radford

**Affiliations:** 1 Astbury Centre for Structural Molecular Biology and School of Molecular and Cellular Biology, University of Leeds, Leeds LS2 9JT, UK

## Abstract

In the early stages of amyloid formation, heterogeneous populations of oligomeric species are generated, the affinity, specificity, and nature of which may promote, inhibit, or define the course of assembly. Despite the importance of the intermolecular interactions that initiate amyloid assembly, our understanding of these events remains poor. Here, using amyloidogenic and nonamyloidogenic variants of β_2_-microglobulin, we identify the interactions that inhibit or promote fibril formation in atomic detail. The results reveal that different outcomes of assembly result from biomolecular interactions involving similar surfaces. Specifically, inhibition occurs via rigid body docking of monomers in a head-to-head orientation to form kinetically trapped dimers. By contrast, the promotion of fibrillation involves relatively weak protein association in a similar orientation, which results in conformational changes in the initially nonfibrillogenic partner. The results highlight the complexity of interactions early in amyloid assembly and reveal atomic-level information about species barriers in amyloid formation.

## Highlights

• Dissection of protein-protein interactions in the early stages of amyloid assembly
• Rare biomolecular collisions and the course of amyloid assembly
• Interaction surfaces and different outcomes of amyloid assembly
• Molecular description of species barriers in amyloid assembly

## Graphical Abstract

**Figure:**
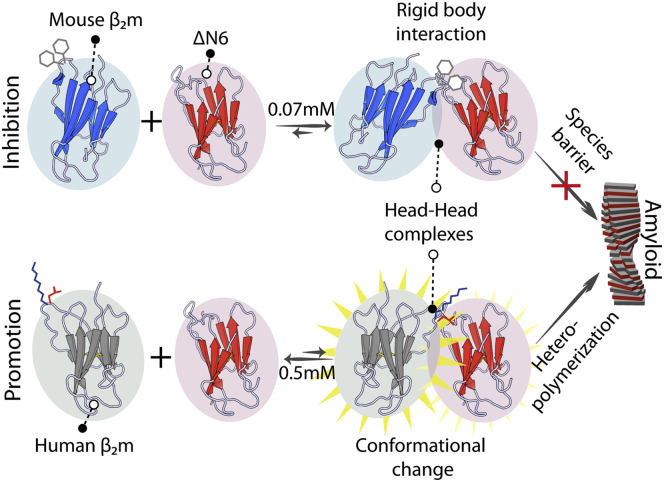

## Teaser

Karamanos et al. identify the interfaces involved in the first steps of amyloid assembly to reveal the origins of interspecies specificity in order to pave the way toward inhibition of amyloid disease.

## Introduction

The assembly of proteins into amyloid fibrils is a complex process requiring specific and sequence-dependent polymerization of initially unfolded or partially folded monomers into fibrils with elaborate cross-β architectures (Greenwald and Riek, 2010). Despite recent insights into the structural characteristics of the amyloid fold (Eisenberg and Jucker, 2012; Fitzpatrick et al., 2013), the process of amyloid assembly is less well understood in structural terms. Assembly from natively folded precursors is commonly initiated by partial unfolding (Chiti and Dobson, 2009). These nonnative species then combine, generating an array of oligomeric intermediates that are transiently populated and usually heterogeneous in mass and conformation (Cremades et al., 2012; Smith et al., 2010). Although recent advances in structural methods have enabled the conformational properties of rarely populated, partially folded monomers of aggregation-prone proteins to be determined (Eichner et al., 2011; Jahn et al., 2006; Neudecker et al., 2012), the nature of the first protein-protein interactions that initiate amyloid formation remains unclear. Early in assembly an array of biomolecular interactions arise, some of which may be productive for amyloid formation, whereas others may be unproductive, with the potential to inhibit or retard amyloid assembly (Campioni et al., 2010; Cremades et al., 2012). The course of amyloid assembly thus may depend on the stability and lifetime of the productive interactions versus their unproductive counterparts. From this viewpoint, identifying and characterizing different biomolecular interactions early in amyloid assembly are crucial for a full understanding of the structural, kinetic, and thermodynamic determinants of amyloid formation and for interpreting phenomena such as species barriers in prion formation. Such information could also pave the way toward the design of molecules able to define or control the course of amyloid assembly.

Amyloid formation is highly specific, with only proteins of closely related sequence capable of copolymerization into amyloid fibrils (Sarell et al., 2013a). Copolymerization may occur by cross-seeding, in which monomers of a different sequence are capable of extending preformed fibrils from a related protein (Giasson et al., 2003; Guo et al., 2013). In other cases, copolymerization may occur prior to the critical nucleation step of fibrillation. In this case, monomers or small oligomers coassemble into assembly-competent species in the early stages of amyloid assembly (Middleton et al., 2012; Sarell et al., 2013b). One such example can be found in prions, proteins that possess at least one conformation that is infectious by being able to transmit their structural and pathological properties onto noninfectious prion monomers (Chien and Weissman, 2001; Tessier and Lindquist, 2009). Interestingly, when prion molecules are transferred to different species they can lose their high infectivity, establishing a species barrier (Chien et al., 2003, 2004), or can confer their toxic conformation onto previously innocuous proteins of a related species (Sindi and Serio, 2009). The molecular determinants of species barriers, however, remain unclear.

**Figure 1:**
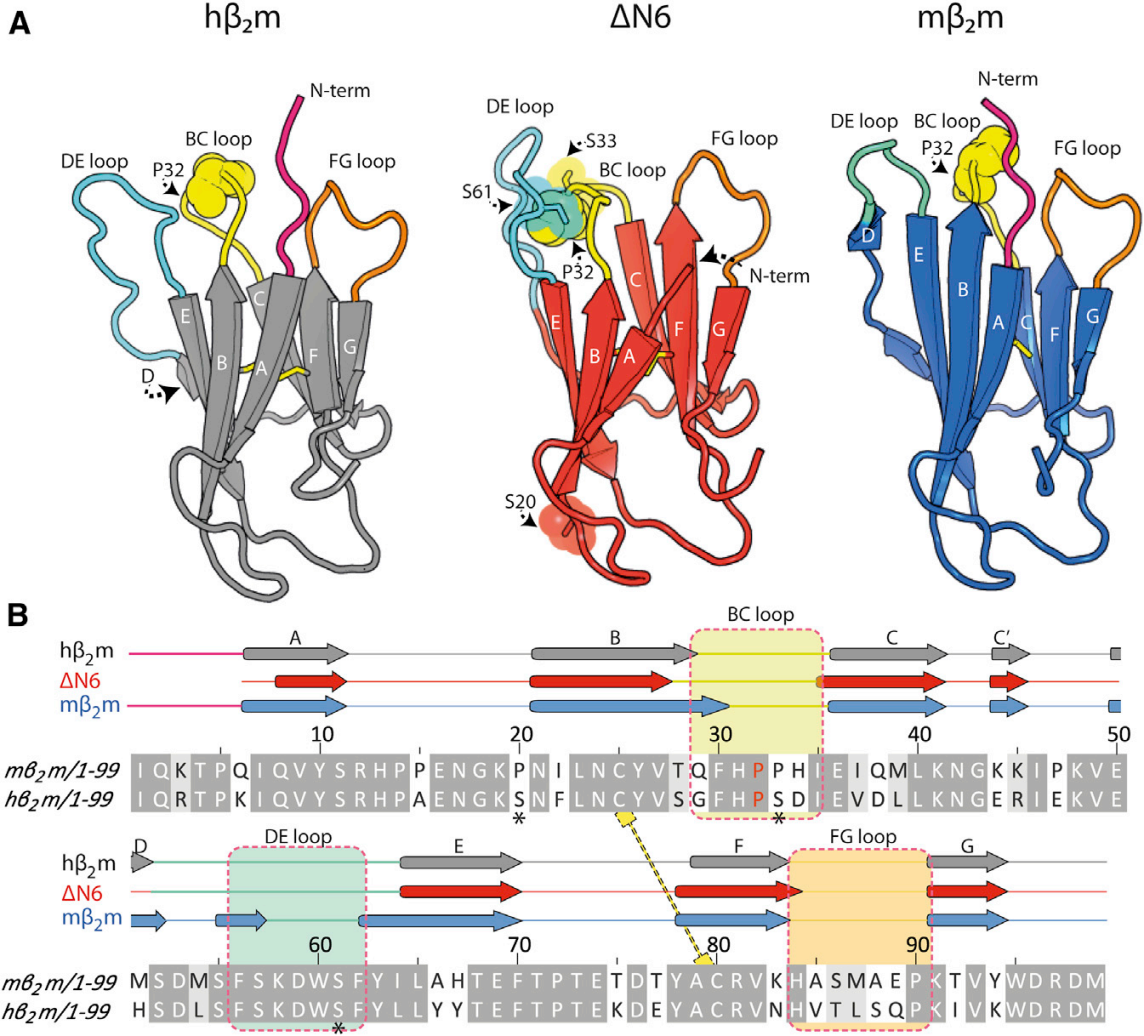
Comparison of β_2_m Variants (A) Structures of hβ_2_m (Protein Data Bank [PDB] ID code 2XKS), ΔΝ6 (PDB ID code 2XKU) (Eichner et al., 2011), and mβ_2_m (PDB ID code 1LK2) (Rudolph et al., 2004) (left to right). (B) Secondary structure and sequence alignment of hβ_2_m and mβ_2_m. Regions identical in sequence are shown in gray. Regions in close spatial proximity to P32 (BC loop, DE loop, FG loop) are highlighted in the structures in (A) and with dashed boxes in (B). The disulfide bond is shown in yellow in (B). P32 is shown as yellow spheres in (A) and highlighted in red in (B). Positions of the spin labels are shown as spheres and sticks in the structure of ΔΝ6 in (A) and with an asterisk in (B).

**Figure 2:**
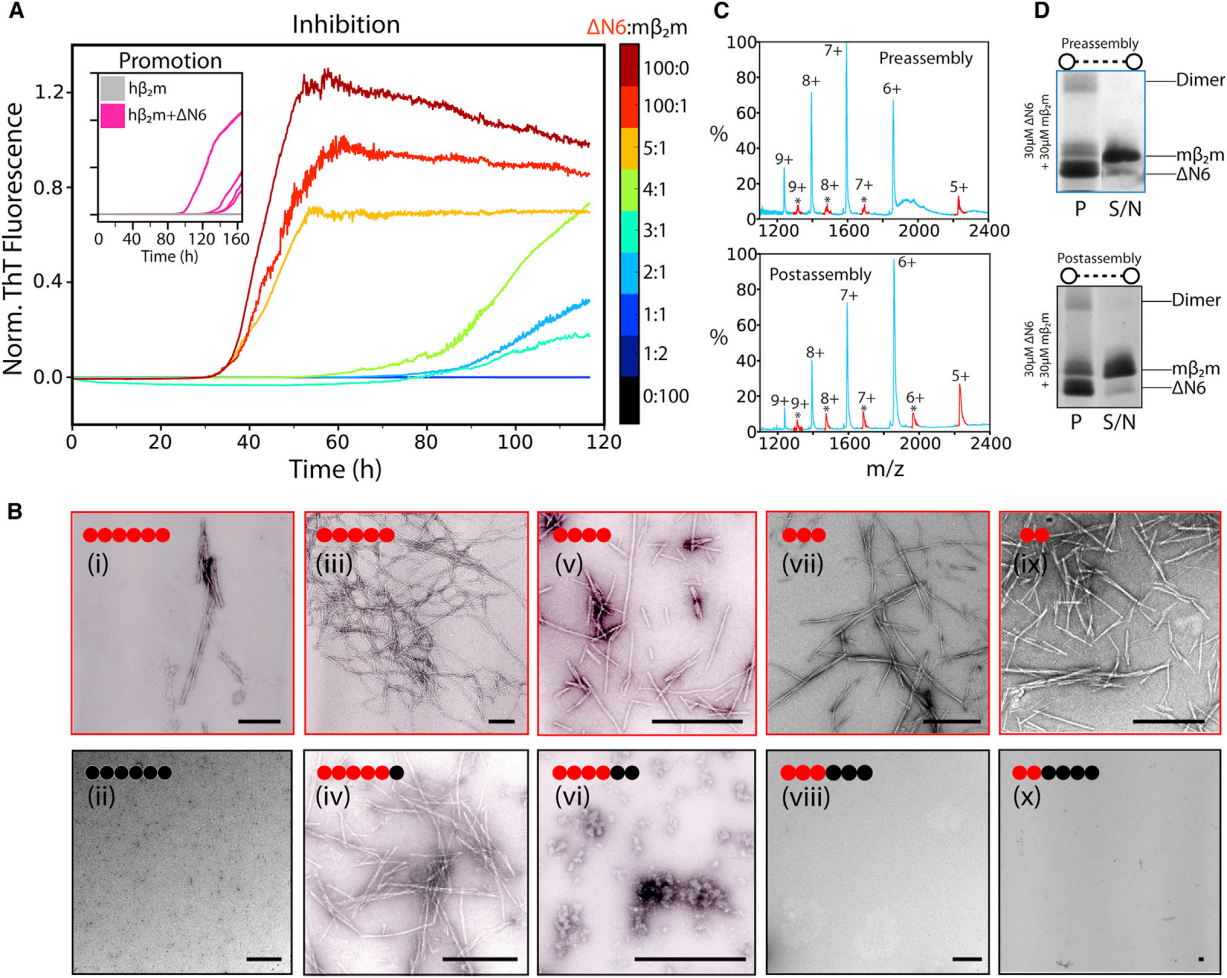
Inhibition of ΔΝ6 Fibril Formation by mβ_2_m (A) Aggregation kinetics of ΔΝ6 alone (dark red) or ΔΝ6 mixed with mβ_2_m in different molar ratios (ΔΝ6:mβ_2_m) measured using ThT fluorescence. The trace with the median lag time is shown. The total protein concentration for all of samples is 60 μM. ThT traces of 60 μΜ hβ_2_m alone (gray) or 59.4 μΜ hβ_2_m mixed with 0.6 μΜ ΔΝ6 (pink; four replicates) are shown (inset). (B) Negative-stain EM images of the endpoint of the reaction (after 120 hr) for sample traces in (A). Black spheres each represent 10 μΜ mβ_2_m and red spheres represent 10 μΜ ΔΝ6. Scale bars represent 100 nm. ΔΝ6 alone, top row; mixtures of ΔΝ6:mβ_2_m, bottom row. (C) ESI mass spectrum of the pellet formed from 30 μΜ ΔΝ6 + 30 μΜ mβ_2_m where the proteins were mixed either prior to fibril assembly (top) or subsequent to assembly (bottom). Fibrils formed after 350 hr of incubation (Figure S1) were pelleted by centrifugation, depolymerized in 100% (v/v) HFIP, and subjected to analysis by ESI-MS. Peaks corresponding to mβ_2_m and ΔΝ6 are shown in red and blue, respectively. (D) SDS-PAGE analysis of the samples shown in (C). P, pellet; S/N, signal to noise.

Here we have explored the nature of protein-protein interactions in the first steps of amyloid formation of β_2_-microglobulin (β_2_m), a 99-residue protein that forms amyloid deposits in dialysis-related amyloidosis (DRA) (Gejyo et al., 1985). Despite being the main constituent of fibrils in DRA, wild-type human β_2_m (hβ_2_m) is not capable of forming amyloid-like fibrils on an experimentally tractable timescale in vitro at neutral pH without the addition of external factors or cosolvents (Calabrese and Miranker, 2009; Eichner and Radford, 2011). By contrast, a truncated variant of β_2_m in which the N-terminal six amino acids are deleted (ΔΝ6), a species that is found in amyloid fibrils in DRA (Esposito et al., 2000), is able to form amyloid fibrils spontaneously at neutral pH in vitro (Eichner et al., 2011; Esposito et al., 2000). NMR studies have shown this variant to be a close structural mimic of a folding intermediate of full-length hβ_2_m that contains a nonnative *trans* X-prolyl bond at Pro32 (I_T_) (Figure 1A), the formation of which has been shown to initiate aggregation (Eichner et al., 2011; Jahn et al., 2006). Importantly, ΔΝ6 is able to convert hβ_2_m into an aggregation-competent state at neutral pH when added in a substoichiometric molar ratio (Figure 2A, inset), in a mechanism reminiscent of conformational conversion associated with prions (Eichner et al., 2011). By contrast with the behaviors of ΔN6 and hβ_2_m, murine β_2_m (mβ_2_m), which is 70% identical in sequence to hβ_2_m (Figure 1B), is unable to form amyloid fibrils at neutral pH (Eichner et al., 2011; Ivanova et al., 2004) and is capable of inhibiting the self-association of ΔΝ6 into amyloid fibrils when added in a stoichiometric ratio (Eichner et al., 2011).

Such diversity in the outcomes of interactions between β_2_m molecules that are similar in sequence and structure provides an ideal system with which to study the principles of protein self-association in amyloid assembly and how different protein-protein interactions can lead to different molecular responses. Here we have combined the powers of different NMR approaches (paramagnetic relaxation enhancement [PRE] and chemical shift perturbation) with other biophysical and biochemical techniques to identify the molecular details of the protein-protein interactions that lead to the promotion (ΔΝ6-hβ_2_m) or inhibition (ΔΝ6-mβ_2_m) of fibril formation. The results reveal that the surfaces involved in the inhibition and promotion of fibrillation are similar. However, the spatial distribution and chemical properties of the generated ensembles differ in detail, sufficient to alter the affinities of these interactions and the effects of the biomolecular collision on the conformational properties of the monomeric precursors involved. Our findings highlight the complexity of the first steps in amyloid assembly, wherein protein association via similar binding surfaces results in different molecular outcomes. They also reveal information about the origins of species barriers in amyloid formation and identify the surfaces to target by molecular design to enable the course of amyloid assembly to be controlled and/or defined.

## Results

### mβ_2_m Kinetically Inhibits ΔN6 Assembly

In previous studies, we have shown that mβ_2_m is able to inhibit the assembly of ΔΝ6 into amyloid-like fibrils when added in a stoichiometric ratio (Eichner et al., 2011), despite the high structural and sequence similarity of the two proteins (sequence identity 70%, sequence homology 90%, root-mean-square deviation 0.91 Å) (Figures 1A and 1B). This phenomenon was further investigated here by measuring the kinetics of fibril formation of ΔΝ6 at pH 6.2 (the pH optimum for ΔΝ6 fibril formation in vitro; Eichner et al., 2011) to which mβ_2_m had been added in different molar ratios. To account for the effect of protein concentration on the kinetics of amyloid formation, the total protein concentration was maintained at 60 μΜ in all experiments. Figure 2A shows that ΔΝ6 assembles into fibrils able to bind thioflavin T (ThT) with lag-dependent kinetics typical of β_2_m amyloid formation (Xue et al., 2008), whereas mβ_2_m does not form fibrils under the conditions employed. Measured over more than ten replicates, the mean lag time of ΔΝ6 assembly was 32.7 ± 3.8 hr, after which time long straight fibrils typical of amyloid formed (Figure 2Bi). When mβ_2_m was mixed with ΔΝ6 in substoichiometric molar ratios, 4:1 ΔΝ6:mβ_2_m or 2:1 ΔΝ6:mβ_2_m, the mean lag time increased to 63.2 ± 3.8 and 91.0 ± 6.2 hr, respectively (Figure 2A), although fibrils formed over the 1 week (120 hr) time course of the experiment using a 5:1 molar ratio of the two proteins (Figure 2B). When the two proteins were mixed in a ≥1:1 molar ratio, complete inhibition ensued (Figure 2A). The dependence of the lag time on the concentration of mβ_2_m added (Figure S1A available online) suggests that inhibition of fibrillation is a kinetically determined process. Accordingly, increasing the molar ratio of mβ_2_m to ΔΝ6 delays, but does not inhibit, the formation of amyloid. In support of this notion, the mixtures of ΔΝ6:mβ_2_m that did not show evidence of fibril formation after 120 hr were incubated for longer periods of time (≥350 hr) and the extent of fibril formation was again measured using ThT fluorescence and negative-stain electron microscopy (EM). These experiments showed that fibrils were able to form after extended incubation times, with the lag time depending on the excess of mβ_2_m added (Figures S1B–S1D). These findings confirm that the interaction between ΔN6 and mβ_2_m retards fibril assembly but, because fibrils are thermodynamically favored, the kinetic barrier to their formation is eventually overcome.

To identify whether mβ_2_m is incorporated into fibrils when mixed with ΔN6, the aggregates formed in samples containing different molar ratios of ΔΝ6:mβ_2_m after 350 hr were collected by centrifugation, depolymerized by incubation at 100°C in SDS-PAGE loading buffer or by incubation in 1,1,1,3,3,3-hexafluoro-2-isopropanol (HFIP), and subjected to analysis by SDS-PAGE or electrospray ionization mass spectrometry (ESI-MS) (Experimental Procedures; Supplemental Experimental Procedures). As a control, fibrils were assembled from ΔΝ6 alone, incubated subsequently with the same concentrations of monomeric mβ_2_m, and analyzed in a similar manner. The results of these experiments showed that mβ_2_m associates with the ΔΝ6 fibrils to a similar extent irrespective of whether the protein was added pre- or postassembly (Figures 2C and 2D). These results indicate that mβ_2_m is not incorporated into the ΔN6 fibrils but associates with the fibril surface subsequent to assembly. By contrast, hβ_2_m has been shown to be incorporated into the fibril core when incubated with ΔN6 in a 1:1 ratio at pH 6.2 (Sarell et al., 2013b).

### Different Binding Affinities for the Inhibition and Promotion of Fibril Assembly

To investigate the interfaces involved in the inhibition (ΔΝ6-mβ_2_m) or promotion (ΔΝ6-hβ_2_m) of amyloid assembly, NMR studies were carried out by mixing ^14^N-labeled ΔΝ6 with ^15^N-labeled mβ_2_m or hβ_2_m (80 μM) and monitoring the chemical shift perturbations upon binding using ^1^H-^15^N HSQC spectra (Experimental Procedures). For both interaction types, the exchange was found to be in the intermediate-to-fast regime (data not shown), giving rise to small, but significant, chemical shift changes upon binding.

**Figure 3:**
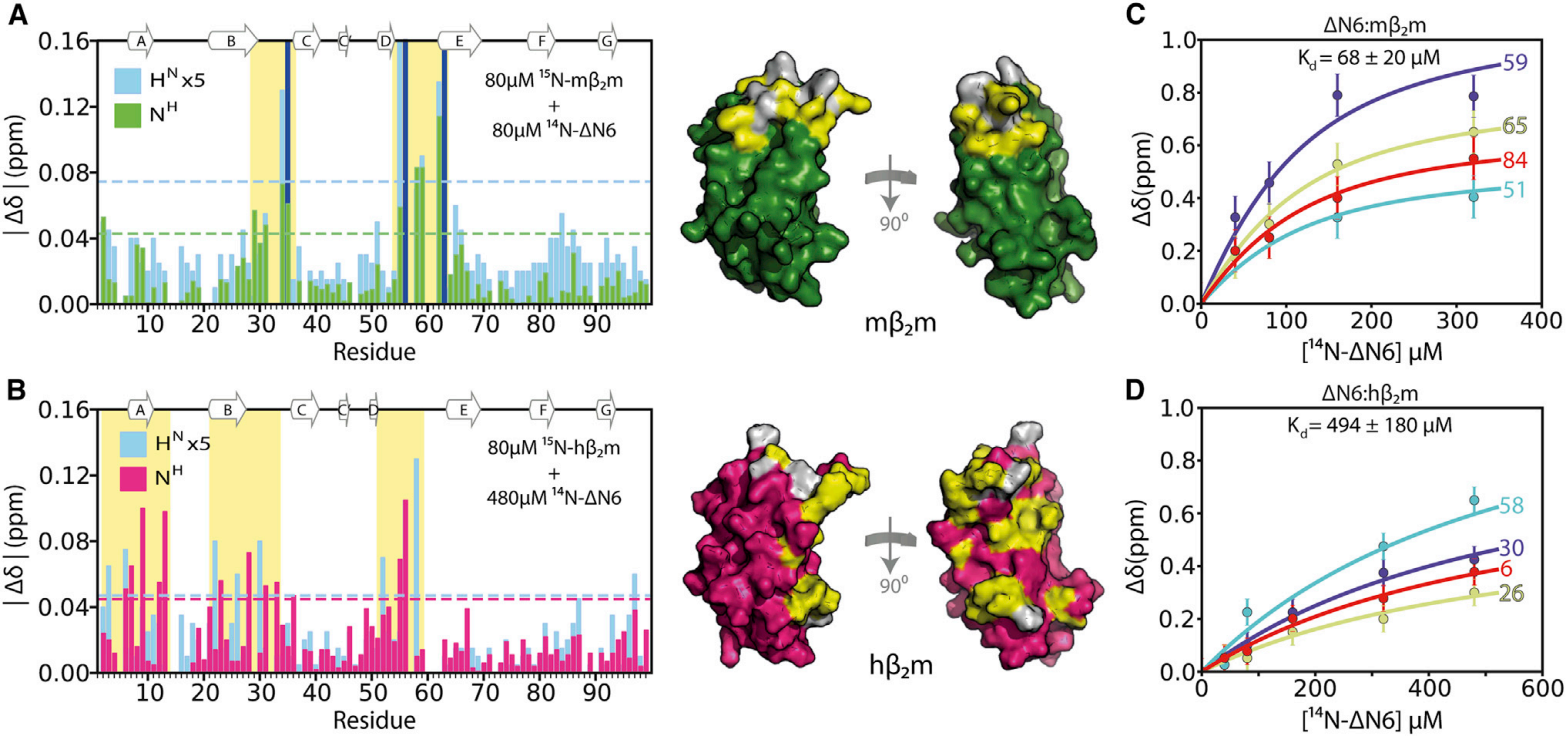
Chemical Shift Changes and Binding Affinities for Different Complexes (A) Chemical shift differences (^1^H, cyan; ^15^N, green) when ^15^N-labeled mβ_2_m and ^14^N-labeled ΔΝ6 are mixed in a 1:1 ratio (80 μΜ each; ∼41% mβ_2_m-bound). All residues experiencing significant chemical shift differences (yellow boxes) locate to the top half of the molecule (BC and DE loops; highlighted in yellow on the surface of the molecule; right-hand side). Residues that show large chemical shift differences in the presence of 40 μΜ ΔΝ6 but are broadened beyond detection at these protein concentrations are marked with dark blue bars. (B) As in (A) but for ^15^N-labeled hβ_2_m and ^14^N-labeled ΔΝ6 mixed in a 1:6 ratio (80 μΜ hβ_2_m; 480 μM ΔΝ6; ∼47% hβ_2_m-bound). Residues with missing assignments are colored gray on the structure of mβ_2_m/hβ_2_m and have missing bars in (A) and (B). Dotted lines in (A) and (B) represent two standard deviations of the mean over the entire data set for each atom type. (C) Plots of the chemical shifts of different residues (51, 59, 65, 84) in ^15^N-labeled mβ_2_m upon titration with ^14^N-labeled ΔN6. Solid lines represent global fits to a binding hyperbola. Error bars were calculated using resonances known not to be involved in the binding interface. For these residues the chemical shift was measured in each spectrum, and the error bars represent the standard deviation of the mean of their peak positions (see Experimental Procedures and Supplemental Experimental Procedures). (D) As in (C) but for ^15^N-labeled hβ_2_m upon titration with ^14^N-labeled ΔN6. Curves for residues 6, 26, 30, and 58 are shown.

In the case of the inhibitory complex (ΔΝ6-mβ_2_m), changes in the ^1^H-^15^N HSQC spectrum, including chemical shift differences and exchange line broadening, were observed for a subset of resonances, even when the proteins were mixed in substoichiometric ΔΝ6:mβ_2_m ratios. Residues that show significantly altered chemical shifts upon binding are localized in the BC and DE loops in the apical region of mβ_2_m (Figure 3A). By contrast, an excess (≥80 μM) of ^14^N-labeled ΔΝ6 was required to observe significant chemical shift changes in the spectrum of ^15^N-labeled hβ_2_m (Figure 3B). In this case, the residues experiencing significant chemical shift differences include the N-terminal regions, the B strand, and the BC and DE loops (Figure 3B). Globally fitting the resulting data (Supplemental Experimental Procedures) yielded K_d_ values of 68 ± 20 μM for the mβ_2_m-ΔN6 interaction and 494 ± 180 μM for the interaction between ΔN6 and hβ_2_m (Figures 3C and 3D). Together, these data suggest a larger interface for the ΔΝ6-hβ_2_m interaction (more residues experience significant chemical shift perturbations) in comparison to its inhibitory ΔΝ6-mβ_2_m counterpart, despite an ∼7-fold decrease in binding affinity.

### Inhibition and Promotion of Fibril Formation Involve Similar Binding Interfaces

Although chemical shifts are excellent probes of protein-protein interactions, they can be affected by long-range effects upon binding (Zhuravleva and Gierasch, 2011). Thus, we next sought to investigate the nature of the protein-protein interactions that lead to inhibition (ΔΝ6-mβ_2_m) or promotion (ΔΝ6-hβ_2_m) of fibril formation in more detail using PRE studies. The PRE depends on the distance between a paramagnet and adjacent nuclei and can provide long-distance (∼30 Å) information quantified by the H_N_-Γ_2_ PRE rate (Supplemental Experimental Procedures) for each amide proton (Clore and Iwahara, 2009). The PRE approach is ideally suited to the analysis of weak intermolecular associations (Clore and Iwahara, 2009), providing distance information that can be used to visualize transient and lowly populated (<0.5%) protein states (Tang et al., 2006, 2008) such as those occurring in the early stages of amyloid formation. To enable these experiments, variants containing a solvent-exposed cysteine were created in ΔΝ6 by mutating either S20 (AB loop), S33 (BC loop), or S61 (DE loop) to cysteine (Figure 1A) while maintaining the disulfide bond involving C25 and C80 (Experimental Procedures). Chemical modification with (1-oxyl-2,2,5,5-tetramethyl-Δ3-pyrroline-3-methyl) methanethiosulfonate (MTSL) yielded ΔΝ6 molecules 100% labeled at a single site (Experimental Procedures). These chemically modified molecules were then used in PRE studies to map the interactions between ^14^N-labeled and MTSL-labeled ΔN6 with ^15^N-labeled hβ_2_m or mβ_2_m, each pair in a stoichiometric ratio (60 μM each) at pH 6.2 and 25°C (Experimental Procedures). Under the conditions employed, and in the absence of agitation, fibril formation does not occur for either pair of proteins over the time course of the experiment (<40 hr). Accordingly, the difference in the ^1^H R_2_ relaxation rates of the ^15^N-labeled protein (hβ_2_m/mβ_2_m) in the presence of oxidized or reduced MTSL-labeled ^14^N-ΔΝ6 was measured (H_N_-Γ_2_ rate) (Experimental Procedures) and used to map the interaction surfaces of the different protein pairs.

**Figure 4:**
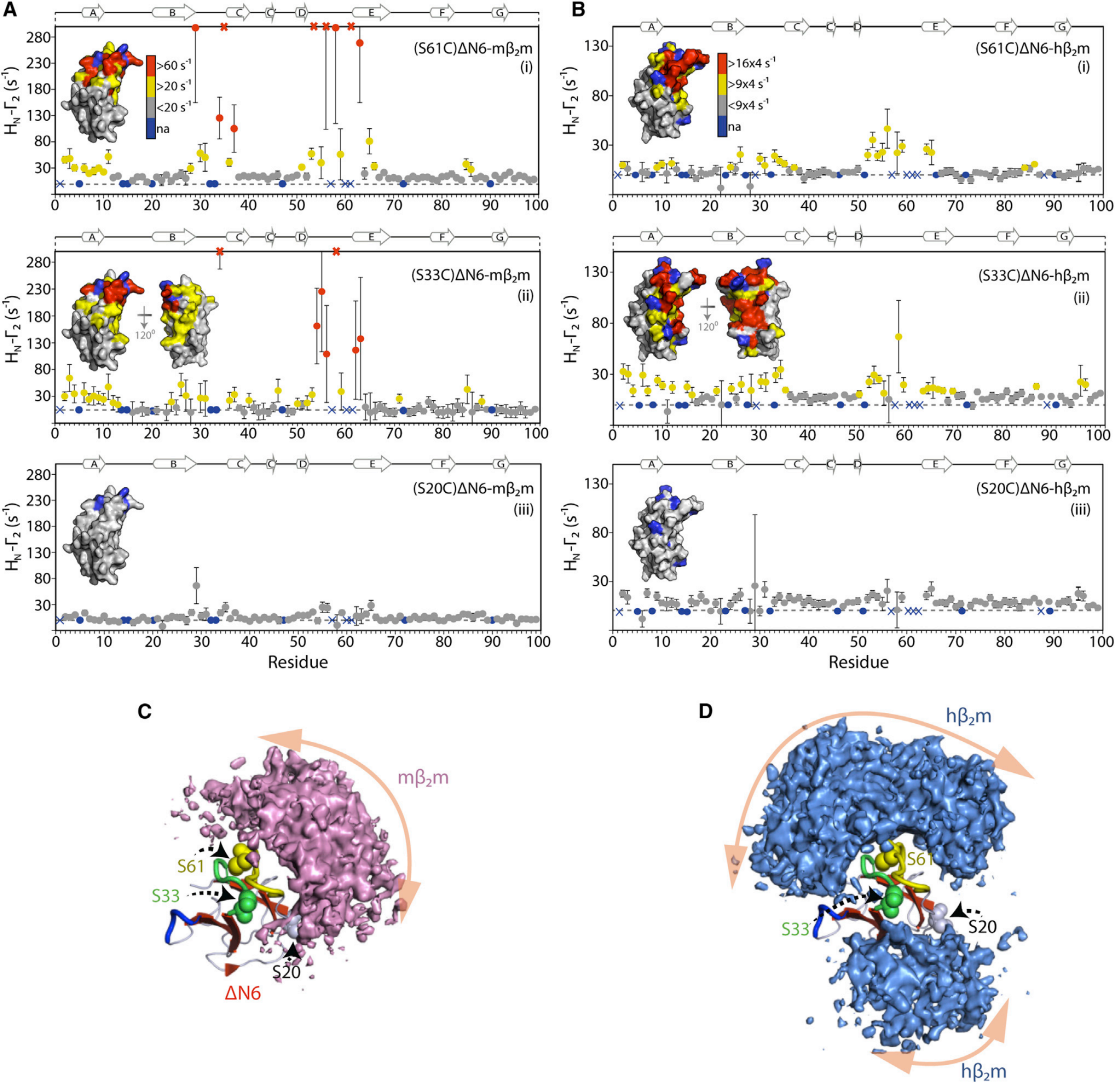
Interaction Interfaces in Different Protein Complexes (A) Per-residue Γ_2_ rates of mβ_2_m (60 μΜ) when MTSL is attached to S61 (i), S33 (ii), or S20 (iii) on ΔN6 (60 μΜ) colored according to their amplitude (blue, not assigned; gray, insignificant; yellow, >20 s^−1^; red, >60 s^−1^; pH 6.2, 25°C). The structure of mβ_2_m as a surface representation colored by the amplitude of the Γ_2_ rates is shown (insets). Red crosses indicate residues for which the Γ_2_ rate is either too large to appear on this scale or resonances broadened beyond detection when the spin label is oxidized and hence the Γ_2_ rate cannot be measured. Blue dots represent proline or overlapping resonances, and blue crosses denote residues for which the assignments are missing. Error bars were calculated from the noise level in the experiment. (B) As in (A) but for the interaction between ^14^N- and MTSL-labeled ΔΝ6 (60 μΜ) and ^15^N-labeled hβ_2_m (60 μΜ). The structure of hβ_2_m is colored according to the amplitude of the Γ_2_ rates after extrapolation to the same % bound as in (A) (blue, not assigned; gray, insignificant; yellow, >9 × 4 s^−1^; red, >16 × 4 s^−1^). Note that the scale is expanded in (B). (C) The distribution of the mβ_2_m molecules in the ΔN6-mβ_2_m complex, with the mβ_2_m ensemble shown as a pink surface around ΔΝ6 (cartoon). The 50 top-scoring ensembles (N = 2, 2 × 50 structures) were included in the calculation. (D) As in (C) but for the ΔΝ6-hβ_2_m association. The pose of ΔΝ6 is identical to (C) and the ensemble of hβ_2_m subunits is colored in blue. The BC, DE, and FG loops of ΔΝ6 are highlighted in green, yellow, and blue, respectively, and the positions of the spin label (S20, S33, and S61) are shown as spheres.

The PRE data collected for the inhibitory interaction between ^14^N-labeled ΔΝ6 (S61C-MTSL) and ^15^N-labeled mβ_2_m are shown in Figure 4Ai and Figure S2A. Backbone assignments for mβ_2_m at pH 6.2 were obtained using standard triple-resonance NMR experiments and uniformly ^15^N/^13^C-labeled protein (Experimental Procedures). The results showed high Γ_2_ values (Γ_2_ > 60 s^−1^) for residues in the BC and DE loops of mβ_2_m and lower Γ_2_ values (<60 s^−1^) for residues in the N-terminal 10 residues and the FG loop. These regions cluster on one side of mβ_2_m surrounding P32 (Figure 1A; Figure 4A, inset), a residue that undergoes *cis*-*trans* isomerization known to be required for amyloid formation from hβ_2_m (Eichner et al., 2011; Sakata et al., 2008). A similar PRE pattern was obtained when the spin label was attached at position 33 (Figure 4Aii). The results suggest that the region of mβ_2_m surrounding P32 is involved in the interaction with ΔΝ6 to create a heterodimer (as supported by analytical ultracentrifugation; see below) that kinetically inhibits amyloid formation. Consistent with this supposition, when the spin label is moved to position 20 on ^14^N-labeled ΔΝ6, the Γ_2_ rates of mβ_2_m in the BC and DE loops are substantially reduced (<25 s^−1^) (Figure 4Aiii), suggesting that S20 is distant from the site of interaction (Supplemental Experimental Procedures). These data suggest, therefore, that a head-to-head configuration of the ΔΝ6-mβ_2_m heterodimer, involving the BC and DE loops from both monomers, creates the inhibitory complex.

Having identified the protein-protein interactions that lead to the inhibition of ΔN6 fibril formation, we next investigated the interactions that lead to ΔΝ6-induced promotion of hβ_2_m fibril assembly. Again, ^14^N-labeled ΔΝ6 was spin labeled with MTSL at residues 61, 33, or 20 and PREs to ^15^N-labeled hβ_2_m were measured (Figure 4B; Figure S2B). In marked contrast with the results obtained for the ΔΝ6-mβ_2_m interaction, the magnitude of the Γ_2_ values is reduced significantly when the spin-labeled ΔΝ6 variants are mixed with hβ_2_m (compare Figures 4Ai and 4Aii with Figures 4Bi and 4Bii), consistent with the ∼7-fold lower K_d_ of the hβ_2_m-ΔΝ6 complex (Figures 3C and 3D). Despite the differences in magnitude of the Γ_2_ rates for the two complexes, the pattern of H_N_-Γ_2_ values obtained is similar to that for the ΔΝ6-mβ_2_m interaction, with the largest PREs observed for residues 55–65 in the DE loop and 26–34 in the BC loop when the spin label is attached at position 61 (Figure 4Bi). Residues in the N-terminal region (residues 2–10) showed increased PRE rates when the spin label is attached at position 33, which were not observed when MTSL was added at position 61 (Figures 4Bi and 4Bii). Again, only very small PREs were observed when MTSL was added at position 20 (Figure 4Biii). These results suggest that the promotion of hβ_2_m fibril formation also involves a head-to-head association of the two monomers.

### Distinct Conformational Ensembles with Structurally Similar Binding Surfaces

To obtain more detailed insights into the protein complexes that give rise to the inhibition or promotion of amyloid formation, the PRE data were used in a rigid body/torsion angle simulated annealing approach to generate structural ensembles of the different complexes by minimizing the difference between the observed and calculated Γ_2_ values. PRE data for each complex obtained using spin labels at positions 33 and 61 in ΔΝ6 were fitted simultaneously, along with data from chemical shift perturbations upon binding that were treated as ambiguous distance restraints (see below and Experimental Procedures). Data arising from spin-labeled ΔΝ6 at position 20 were not included (Supplemental Experimental Procedures). The population of the interconverting species was set to 18% in both cases based on the known K_d_s of each complex.

**Figure 5:**
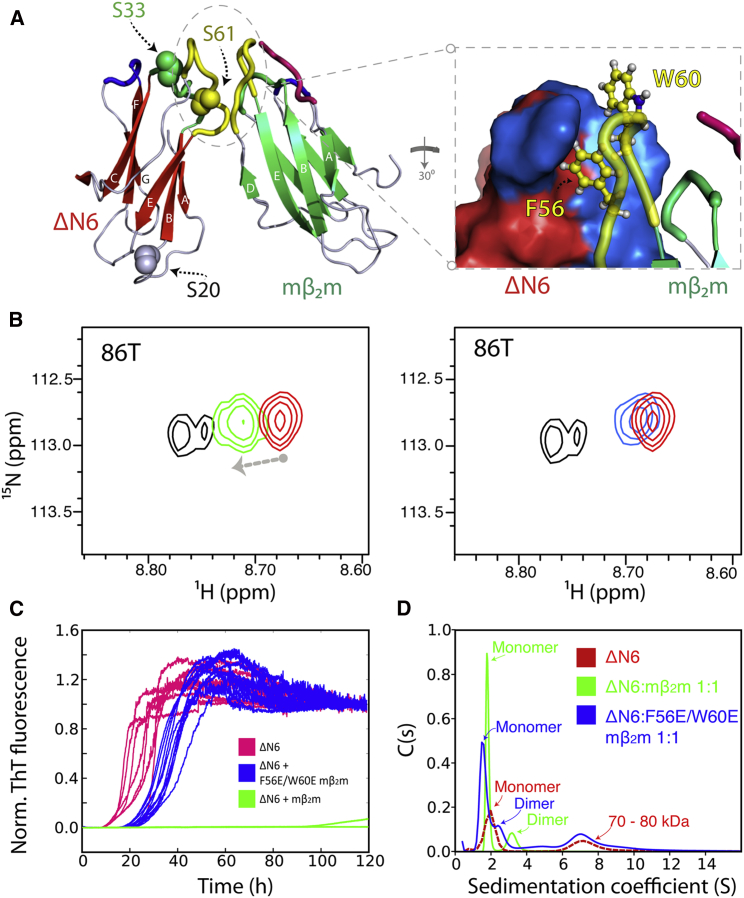
F56 and W60 in mβ_2_m Form Interactions Required for Amyloid Inhibition (A) The lowest-energy calculated structure of the ΔN6 (red)-mβ_2_m (green) complex highlighting F56 and W60 in the interface. Interface residues are colored blue on ΔΝ6 (right). (B) Representative sample resonances in the ^1^H-^15^N HSQC spectrum of ^15^N-labeled ΔΝ6 (80 μM; red) that show chemical shift changes upon the addition of ^14^N-labeled mβ_2_m (green) but not its F56E/W60E variant (160 μM; blue). Addition of mβ_2_m shifts the resonances of ΔΝ6 toward their positions at pH 8.2 (black), where ΔN6 is not amyloidogenic (Eichner et al., 2011) (additional examples are shown in Figure S5A). (C) Fibrillation kinetics of ΔΝ6 alone (20 μΜ; pink) at pH 6.2 and in the presence of a 2-fold molar excess of mβ_2_m (green) or F56E/W60E mβ_2_m (blue). (D) Sedimentation velocity AUC traces of ΔΝ6 alone (60 μM; red), ΔΝ6 (60 μM) mixed with an equimolar concentration of mβ_2_m (green), or F56E/W60E mβ_2_m (blue).

In a first series of simulated annealing calculations, the interconverting species were represented as a single conformer (N = 1) (Experimental Procedures). The results of this analysis revealed a head-to-head configuration for the association of ΔΝ6 with mβ_2_m in which the DE loops from each monomer make the majority of the intermolecular contacts (Figure 5A). Interestingly, the high Q factor (0.54; Figure S2C; Supplemental Experimental Procedures) suggests that multiple conformations are required to satisfy the experimental restraints. In exchanging systems the observed PRE rate is the weighted population average of the species in solution, as long as those are in the fast exchange regime (Iwahara et al., 2004). In this case, the PRE methodology allows the visualization of the ensemble of the interconverting species. Increasing the number of conformers to two (N = 2) results in a significant decrease in the Q factor for the ΔΝ6-mβ_2_m interaction (Q = 0.37), with no further significant decrease (Q = 0.36) when N is increased to three (Figures S2C and S2D). Similar analysis of the ΔΝ6-hβ_2_m association revealed that (at least) two conformers are also required to describe the experimentally measured PRE data (Figures S2E and S2F).

The associating monomers in the ΔΝ6-mβ_2_m and ΔΝ6-hβ_2_m ensembles were visualized as atomic probability density maps as described (Tang et al., 2006) (Figures 4C and 4D). The resulting ensemble for the ΔΝ6-mβ_2_m complex shows that mβ_2_m molecules cluster around the DE loop of ΔΝ6 (residues 52–63), which makes the majority of contacts with mβ_2_m (Figure 4C; Figure S3A; Movie S1). ΔΝ6, by contrast, shows a bimodal distribution around the DE loop of mβ_2_m, with one cluster of molecules facing the β sheet composed of the A, B, E, and D strands, whereas the second cluster of ΔΝ6 molecules locates opposite the edge strands D and C (Movie S2). On the other hand, the ΔΝ6-hβ_2_m interaction is more heterogeneous, extending to both sides of the apical region of ΔΝ6 (around P32) (Figure 4D; Figure S3B; Movie S1). The volume of the ΔΝ6-mβ_2_m density map is calculated to be 7,157 Å^3^, whereas that of the ΔΝ6-hβ_2_m cluster is almost twice as large (13,670 Å^3^; a cutoff of 40% was used in both cases; Table S1). Interestingly, the distributions of mβ_2_m and hβ_2_m molecules around ΔΝ6 do not completely overlay. Areas showing high intermolecular contacts unique to the ΔΝ6-hβ_2_m complex involve the BC and FG loops of ΔΝ6 interacting with the BC and DE loops of hβ_2_m (Figure S3B). A correlation between the hydrophobic surface area of mβ_2_m (shaped mainly by the region surrounding the DE loop) and the distribution of ΔN6 molecules is observed, indicating that this interaction interface is predominantly hydrophobic in nature, with residues F56, W60, and F62 participating in key intermolecular contacts (Figure S3C; Movie S2). By contrast, the apical region of hβ_2_m (DE, BC, and FG loops) displays less solvent-exposed hydrophobic surface area and a greater predominance of charged residues that reflect the differences in the sequence of the proteins in these regions (Figure 1; Figure S3D; Table S1; Movie S2). Together, the results indicate that inhibition of ΔΝ6 fibril formation involves a “specific” head-to-head protein association driven by hydrophobic interactions with mβ_2_m. On the other hand, the ΔΝ6-hβ_2_m interaction, although also adopting a head-to-head configuration, is weaker, more heterogeneous, and involves electrostatic interactions. Whether these data reflect the formation of a range of “encounter complexes” between ΔΝ6 and hβ_2_m that is not observed for the ΔΝ6-mβ_2_m interaction, or whether they report on the transient formation of higher-order oligomers between ΔΝ6 and hβ_2_m, remains to be resolved.

### Mutation of Aromatic Residues Prevents Inhibition of ΔN6 Assembly by mβ_2_m

To confirm that the head-to-head association of ΔΝ6 with mβ_2_m is involved in inhibition of fibril formation, two amino acid substitutions (F56E and W60E) were introduced into mβ_2_m at sites that were found to participate in the majority of intermolecular contacts between the two molecules (Figure 5A; Figure S4A). The ability of this variant to bind to ΔN6 and to inhibit fibril assembly was then monitored using NMR and ThT fluorescence assays, respectively. When ^14^N-labeled F56E/W60E mβ_2_m (160 μM) was mixed with ^15^N-labeled ΔΝ6 (80 μM) at pH 6.2, only small changes in the chemical shifts of ΔΝ6 (∼20% in comparison to wild-type mβ_2_m) were observed in the BC, DE, and FG loops (Figure 5B; Figures S5A and S5B), consistent with the proteins no longer interacting tightly. Consistent with these observations, F56E/W60E mβ_2_m is unable to inhibit ΔN6 fibril assembly when added in a 2-fold molar excess (Figure 5C; Figures S5C and S5D), conditions under which wild-type mβ_2_m delays the onset of amyloid for more than 120 hr (Figure 2A; Figure S1C). The interaction of wild-type mβ_2_m with ΔΝ6 prevents the formation of oligomeric species by the latter protein as observed by sedimentation velocity analytical ultracentrifugation (AUC) (Figure 5D), resulting in a monomer-dimer (∼80:20) equilibrium, consistent with a specific interaction as suggested by the analysis of the PRE data. Notably, under identical conditions, the F56E/W60E variant abolishes the ability of the murine protein to dissociate preformed oligomers of ΔΝ6 (Figure 5D).

### Binding-Induced Unfolding versus Rigid Body Docking: A Rationale for the Outcome of Biomolecular Collision

**Figure 6:**
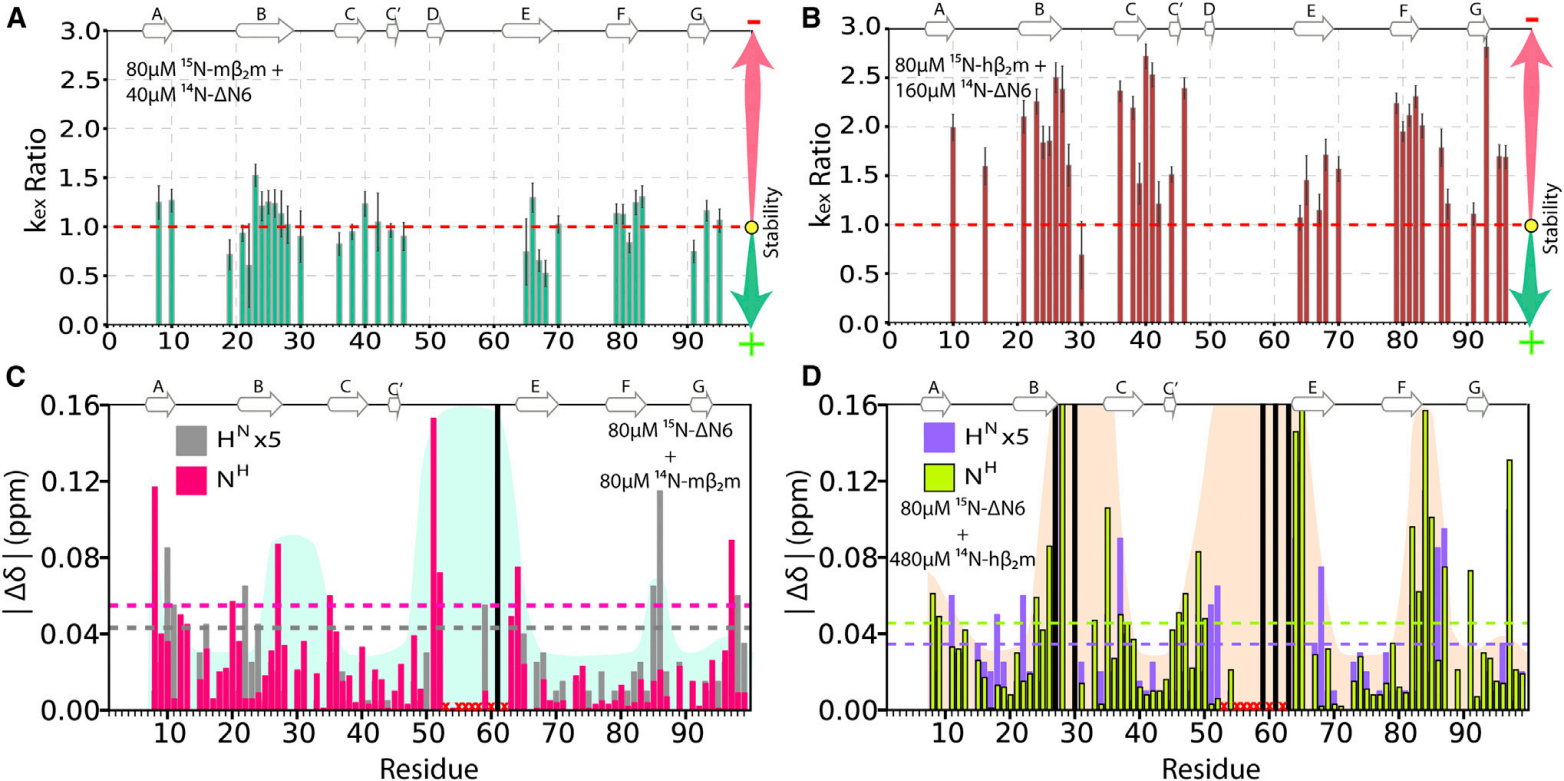
Dynamic versus Rigid Body Interactions in Different Protein Complexes (A) The ratio of the H/D exchange rates of ^15^N-labeled mβ_2_m bound (∼22%) to unlabeled ΔΝ6 (80 μΜ mβ_2_m + 40 μΜ ΔΝ6) versus free (80 μΜ) ^15^N-labeled mβ_2_m (k_ex_ ratio, bound:free) plotted against residue number. An increase in k_ex_ ratio indicates a loss of H/D exchange protection upon binding. Error bars represent the propagated error of the fits to the raw data shown in Figure S6. (B) As in (A) but for free (80 μΜ) ^15^N-labeled hβ_2_m versus ∼22% ^15^N-labeled hβ_2_m bound to ΔΝ6 (80 μΜ hβ_2_m + 160 μΜ ΔΝ6). Note that exchange of hβ_2_m occurs by a mixed EX1/EX2 mechanism (Hodkinson et al., 2009), ruling out analysis of these data in terms of the free energy of binding. (C) Differences in ^1^H (gray) and ^15^N (red) chemical shifts when ^15^N-labeled ΔΝ6 and ^14^N-labeled mβ_2_m are mixed in a 1:1 ratio (80 μΜ each; ∼45% ΔΝ6-bound). Dotted lines represent two standard deviations of the mean over the entire data set for each nucleus. (D) As in (C) but for ^15^N-labeled ΔΝ6 mixed with ^14^N-labeled hβ_2_m (80 μΜ hβ_2_m + 480 μΜ ΔΝ6; ∼45% ΔΝ6-bound). Black bars denote residues that are broadened beyond detection but show significant chemical shift changes when less mβ_2_m/hβ_2_m is added. Red crosses denote ΔΝ6 residues that are broadened at pH 6.2. Residues that experience significant chemical shift changes on binding are highlighted in blue and pink backgrounds in (C) and (D), respectively.

To investigate why biomolecular collision of hβ_2_m or mβ_2_m with ΔN6 results in different outcomes of assembly, the effect of ΔN6 binding on the conformational dynamics of each monomer was measured using hydrogen-deuterium (H/D) exchange. In each case, the rate of H/D exchange of monomeric (unbound) hβ_2_m/mβ_2_m was compared with its ΔΝ6-bound counterpart at pH 6.2, using samples in which the protein concentrations of hβ_2_m/mβ_2_m were adjusted to generate complexes containing a similar percent (∼20%) of ΔΝ6-bound hβ_2_m/mβ_2_m monomer. These experiments showed that the (H/D) exchange rates of mβ_2_m are unaffected (k_ex_ increases by less than ∼1.3-fold) upon interaction with ΔΝ6 (Figure 6A; Figure S6A). By contrast, the addition of ΔΝ6 to hβ_2_m causes a 2- to 3-fold increase in the H/D exchange rates of residues throughout the sequence of hβ_2_m (Figure 6B; Figure S6B), consistent with an increase in global dynamics of the protein upon interaction with ΔN6. These results were confirmed using a variety of ΔΝ6 concentrations for both complexes, ranging from 40 to 320 μΜ.

Close examination of the chemical shift changes that occur when ^14^N-labeled ΔΝ6 is added to ^15^N-labeled mβ_2_m reveals that the residues that undergo significant chemical shift changes also experience increased PRE rates (BC and DE loops), confirming that these regions of the protein form the interaction interface (Figure 3A). On the other hand, residues in the N-terminal region including the AB loop of hβ_2_m (residues 12–13) show significant chemical shift changes upon binding to ΔΝ6 (Figure 3B) but minor PREs (Figure 4B), consistent with these residues not being involved in the interface of the lowest-energy structures of the ΔΝ6-hβ_2_m complex (Figure S3B). These observations suggest that the binding of ΔΝ6 to hβ_2_m provides sufficient energy to alter the conformation of the N-terminal 12 residues of hβ_2_m (observed previously by H/D exchange and relaxation NMR methods; Eichner et al., 2011) such that a more amyloidogenic conformation is adopted. By contrast, the nonamyloidogenic (and thermodynamically less stable) mβ_2_m (ΔG_un_° _mouse_ = −10.7 kJ/mol, ΔG_un_° _human_ = −22.5 kJ/mol; C. Pashley and S.E.R., unpublished data) is not affected significantly by binding. Differences in cooperativity or local stability of the interacting monomers thus dictate the progress of amyloid assembly.

Finally, the consequences of binding on the conformational properties of ΔΝ6 were investigated by measuring the changes in the chemical shifts of ^15^N-labeled ΔΝ6 (80 μM) upon titration with ^14^N-labeled mβ_2_m (80 μM) or ^14^N-labeled hβ_2_m (480 μM) (∼45% ΔΝ6 bound in each case) (Figures 6C and 6D). Significant chemical shift differences were observed for residues in the BC and DE loops of ΔN6 upon binding to hβ_2_m and mβ_2_m, consistent with the head-to-head structure of both complexes. The larger number of ΔΝ6 residues showing chemical shift differences observed upon binding and the greater Δδ observed for the ΔΝ6-hβ_2_m complex are consistent with the larger interface of this interaction, but could also suggest that ΔΝ6 responds to binding hβ_2_m by undergoing conformational change. The picture that emerges, therefore, is that the promotion of hβ_2_m fibril formation by ΔΝ6 involves weak binding that nonetheless leads to conformational changes in one or both of the interacting partners. By contrast, the ΔΝ6-mβ_2_m complex, even though employing a similar head-to-head interaction, involves the formation of a relatively specific, tight binding, inhibitory complex with little or no effect on the conformational properties of the interacting partners.

## Discussion

### Protein Interaction Surfaces and the Molecular Mechanism of β_2_m Aggregation

Amyloid fibrils share similar structural features based upon a cross-β core, irrespective of the organism of origin, the protein involved, or the sequence of the protein precursor (Eisenberg and Jucker, 2012). Despite their similarity in structure, amyloid fibrils can be beneficial to the organism concerned, whereas for others amyloid formation is deleterious (Otzen, 2010). For each scenario, mechanisms have evolved that either facilitate assembly or protect against the accumulation of aggregation-competent proteins, depending on whether the fibrils are beneficial or not (Bucciantini et al., 2002; Otzen, 2010; Maji et al., 2009). One such example can be found in prions, proteins that possess at least one amyloid-competent conformation that is infectious by being able to transmit its structural and pathological properties onto innocuously folded prion monomers (Sindi and Serio, 2009). When prion molecules are transferred between species, they can lose their infectivity or allow propagation depending on the organism involved, establishing a so-called species barrier (Chien et al., 2003; Tessier and Lindquist, 2009; Baskakov, 2014). The precise molecular details of how and why species barriers occur between very similar proteins remain unclear. ΔΝ6 has been shown to possess prion-like properties in its ability to convert hβ_2_m in an aggregation-prone conformation by biomolecular collision (although the protein is not infectious) (Eichner et al., 2011). Here we show that the prion-like characteristics of ΔΝ6 are not only limited to its ability to convert hβ_2_m into an amyloid-competent conformation but also in its ability to experience species barriers (when the molecule interacts with mβ_2_m, amyloid assembly is inhibited). The results show that aggregation propensity is not simply related to the kinetic and/or thermodynamic properties of the proteins involved (the least stable β_2_m variant studied here [mβ_2_m] inhibits assembly, whereas propagation involves interaction of ΔN6 with the most stable variant [hβ_2_m]). Instead, the fate of amyloid assembly involves a fine interplay between molecular recognition and protein plasticity, which is governed by the precise location and chemical properties of the interfaces involved in the first biomolecular interaction events.

### Interactions that Result in Inhibition or Promotion of Amyloid Assembly

Amyloid diseases are usually late-onset disorders, with symptoms appearing many decades into life, even for individuals carrying the most deleterious of mutations (Greenwald and Riek, 2010). Why this is the case remains unclear; possibilities include the time taken to nucleate fibril formation, and/or atrophy or overload of the proteostatic mechanisms that protect cells from protein misfolding and aggregation (Balch et al., 2008). Defining the nature of the complex network of protein-protein interactions that form in the earliest stages of amyloid assembly is of crucial importance, therefore, in our quest to understand the events that initiate protein aggregation at a molecular level. Such knowledge will also open the door to the design of inhibitors able to arrest amyloid formation by targeting specific surfaces that block the formation of fibrils and their toxic precursors, thereby halting the disease process at its outset.

**Figure 7:**
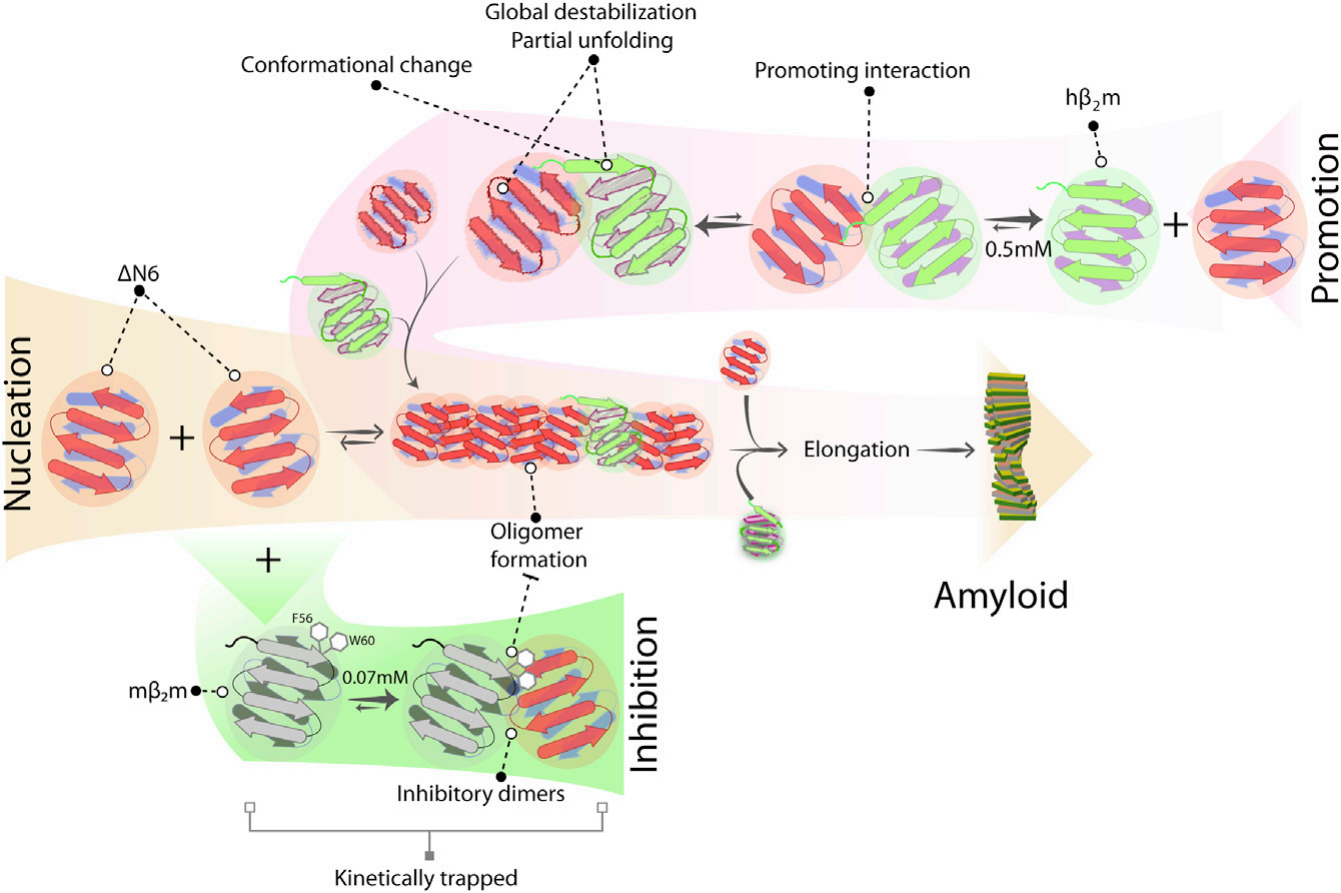
Model for the Nucleation, Inhibition, and Promotion of ΔΝ6 Fibril Formation ΔΝ6 self-assembles into amyloid fibrils in a reaction that involves the formation of preamyloid oligomers (middle). mβ_2_m has the ability to push the equilibrium back to monomers and trapped heterodimers, destroying or delaying the formation of the critical nucleus and kinetically inhibiting the formation of fibrils. This interaction involves the DE loops of both molecules and results in the accumulation of kinetically trapped heterodimers (bottom). hβ_2_m interacts with ΔΝ6 in a similar head-to-head manner as mβ_2_m, but this interaction causes conformational changes and/or partial unfolding of hβ_2_m. The destabilization of the native fold generates species with increased amyloid potential, presumably facilitated by *cis*-*trans* isomerization of P32 in destabilized, ΔN6-bound hβ_2_m, explaining the mechanism by which ΔΝ6 is able to enhance the amyloid potential of hβ_2_m.

Attempts to identify the intermolecular interactions that form early in amyloid assembly have remained a significant challenge as a consequence of the interactions’ heterogeneity and transient nature (Cremades et al., 2012). By exploiting the power of biomolecular NMR methods and applying them to β_2_m sequences from different species, we have been able to define the intermolecular surfaces that determine the course of amyloid assembly. Specifically, we show that the interaction of ΔΝ6 with mβ_2_m inhibits aggregation via trapping the amyloidogenic precursor (ΔN6) in kinetically stable dimers (K_d_ = 68 ± 20 μΜ). These involve the formation of a relatively well defined interface, stabilized by hydrophobic interactions involving the side chains of residues in the DE and BC loops of both molecules, including F56 and W60 (Figure 7, bottom). Interestingly, mβ_2_m is the least stable variant of the three β_2_m homologs studied here, as shown by its increased H/D exchange rates and decreased unfolding free energy relative to ΔN6 and hβ_2_m (T.K.K., C. Pashley, and S.E.R., unpublished data). Thermodynamically and kinetically unstable proteins, therefore, and not only their stable counterparts (e.g., antibodies or affibodies; Dumoulin et al., 2003; Hoyer et al., 2008), can act as efficient and specific inhibitors of aggregation. Surprisingly, the amyloid-promoting association of ΔΝ6 with hβ_2_m also involves a head-to-head interaction similar, but not identical, to that of the inhibitory complex. Consistent with this finding, the folding intermediate I_T_ of hβ_2_m that structurally resembles ΔN6 (Eichner and Radford, 2009; Eichner et al., 2011) was recently shown to form transient oligomers during folding that are also organized in a head-to-head configuration, although the structures formed and their implications for aggregation were not described (Rennella et al., 2013).

We show here that the amyloid-promoting interaction between ΔΝ6 and hβ_2_m is thermodynamically weaker than its inhibitory counterpart (K_d_ = 494 ± 180 μΜ) and involves multiple interaction sites that involve complementary electrostatic interactions between the interacting molecules that are not utilized in its inhibitory ΔΝ6-mβ_2_m counterpart. These differences in the interaction interfaces result in binding-induced conformational changes in hβ_2_m that are manifested by a 2- to 3-fold increase in its hydrogen exchange rates (Figure 7, top). This interaction also alters the conformation of the AB loop of hβ_2_m, as shown previously (Eichner et al., 2011). Accordingly, ΔN6 is able to act as protein saboteur, each molecule interacting with numerous copies of hβ_2_m, destabilizing the native fold of hβ_2_m and allowing P32 to relax from its native *cis* isomer to its *trans* form, which then traps the protein irreversibly in an aggregation-competent state. *cis* Pro32 in hβ_2_m, therefore, acts as a key switch in amyloid formation. Accordingly, any event that promotes relaxation of Pro32 to the *trans* conformer (mutation, formation of ΔN6 or I_T_, interaction with Cu^2+^ ions, chaperones, or proline isomerase) promotes formation of amyloid fibrils (reviewed in Eichner and Radford, 2011).

### Implications for the Origins of Transmissibility in Amyloid Diseases

The results presented reveal that subtle differences in the nature of protein-protein interactions can give rise to fundamentally different outcomes of amyloid assembly that depend on the affinity of the interaction, the stability of the interacting partners, and the chemical nature of the interacting surfaces. The results have significance that extends beyond the specific case of the β_2_m variants studied here. The catalytic templating model proposed to explain the conversion of the cellular human prion protein (PrP^C^) to its infectious scrapie form (PrP^SC^) is one such case (Aguzzi et al., 2008). Accordingly, mutations that have little effect on the structural and thermodynamic properties of the monomeric PrP precursors (Bae et al., 2009) could alter the surface properties of the protein, influencing the network of intermolecular interactions formed, and hence lead to increased or decreased infectivity. Other amyloid proteins that are intrinsically disordered (such as A_β40_ and α-synuclein) are known to mutually enhance each other’s aggregation (Guo et al., 2013), possibly involving a similar mechanism of binding-induced conformational change. Indeed, heteropolymerization in amyloid assembly seems to be more common than initially anticipated (Sarell et al., 2013a). As shown here, protein association, response to binding, and the effect of transient intermolecular association on the course of assembly are all interlinked. Binding, even to similar surfaces, can cause a different response on the partners involved and thus lead to a different outcome of assembly. The HET-S/HET-s prion strains in filamentous fungi represent another example (Greenwald et al., 2010). HET-S, even though 97% identical in sequence to HET-s, does not aggregate, and can also inhibit the propagation of the prion form of HET-s by biomolecular interaction, resembling the effect of mβ_2_m on ΔΝ6 assembly. A model for prion inhibition by HET-S has been proposed in which HET-S, although able to interact with HET-s and adopt the amyloid β-solenoid fold, is incompetent for further polymerization (Greenwald et al., 2010), further highlighting the observation that collision of similar proteins can result in different outcomes of assembly. Application of the approach taken here for β_2_m to other proteins involved in human disease, including the classic examples of species barriers in PrP propagation (Baskakov, 2014), prion compatibility in yeast and other fungi (Tessier and Lindquist, 2009), and other proteins purported to be infectious (Brundin et al., 2010), will reveal the similarities and distinctions between ΔN6-induced conformational conversion and amyloid inhibition and the molecular events occurring in other systems.

As well as providing insights into the molecular origins of species barriers in amyloid formation, the results presented provide opportunities for the design of molecules to control amyloid disease by targeting intermolecular contacts in the specific surfaces involved. The design of small molecules able to disrupt protein-protein interactions and the generation of other reagents (antibodies, affibodies, or nucleic acid aptamers [Bunka et al., 2007] selected to bind to a specific surface) are exciting possibilities for future avenues of research. The complexity of amyloid assembly, especially in the cellular environment, may require multiple routes involving different strategies to delay, prevent, or revert disease to be deployed simultaneously (for example by combining interference of protein assembly with small molecules or aptamers in concert with regulation of the cellular mechanisms that recognize protein misfolding events). The ability to target the earliest biomolecular events in the aggregation cascade offers potential for a route toward amyloid therapy that will add to the arsenal of approaches currently being developed to combat these devastating disorders.

## Experimental Procedures

### Protein Preparation

hβ_2_m, mβ_2_m, and ΔΝ6 (^14^N- and ^15^N-labeled) and their variants were expressed and purified as described (Platt et al., 2005).

### Assembly of Amyloid-like Fibrils

Samples containing 0.6–60 μΜ protein, 10 mM sodium phosphate buffer (pH 6.2), 83.3 mΜ NaCl (total ionic strength 100 mM), 0.02% (w/v) sodium azide, and 10 μΜ ThT were incubated at 37°C in sealed 96-well plates with agitation at 600 rpm (Supplemental Experimental Procedures).

### PRE Experiments

The ΔN6 variants (^14^N-labeled) C20S, C33S, and S61C modified with MTSL (Supplemental Experimental Procedures) were mixed with ^15^Ν-labeled hβ_2_m or mβ_2_m (60 μM, unless otherwise stated) in 10 mM sodium phosphate buffer (pH 6.2) and H_N_-PRE data were measured as described in Supplemental Experimental Procedures.

### Simulated Annealing Calculations

All structure calculations were performed using a torsion angle-simulated annealing protocol in XPLOR-NIH as described (Iwahara et al., 2004) (Supplemental Experimental Procedures).

### K_d_ Measurements

Binding affinities for the complexes of mβ_2_m and hβ_2_m with ΔN6 were determined at pH 6.2 and 25°C by titrating 80 μΜ ^15^N-labeled mβ_2_m with 0–320 μΜ ^14^N-labeled ΔN6 or 80 μΜ ^15^N-labeled hβ_2_m with 0–480 μΜ ^14^N-labeled ΔN6 and measurement of the resulting chemical shift changes using ^1^H-^15^N HSQC spectra (Supplemental Experimental Procedures).

### Hydrogen Exchange Experiments

The rate of H/D exchange of samples of ^15^N-labeled hβ_2_m or mβ_2_m (80 μM) alone or mixed with ^14^N-labeled ΔN6 (160 or 40 μM, respectively) to produce ∼22% bound complexes in each case was measured at pH 6.2. Hydrogen exchange was measured using SOFAST-HMQC NMR methods as previously described (Schanda et al., 2005) (Supplemental Experimental Procedures).

### Additional Procedures and Further Information

Detailed description of all other methods and protocols can be found in Supplemental Experimental Procedures.

## Accession Numbers

Assignments for the backbone atoms of mβ_2_m have been deposited in the BioMagResBank under accession number 19772.

## Supplemental Information

Document S1. Supplemental Experimental Procedures, Figures S1–S6, and Table S1

Movie S1. The mβ_2_m-ΔN6 and hβ_2_m-ΔN6 Complexes Involve Different Subunit Orientations of a Common Head-to-Head Dimer, Related to Figure 4 Movie animation of the structural ensembles shown in Figures 4C and 4D. ΔΝ6 is shown as a cartoon representation with its BC loop highlighted in green, the DE loop in yellow, and the FG loop in blue. The ensemble of mβ_2_m molecules around ΔΝ6 is shown as a pink surface on the left-hand side, whereas the hβ_2_m ensemble is shown as a blue surface on the right-hand side.

Movie S2. The mβ_2_m-ΔN6 and hβ_2_m-ΔN6 Complexes Show Different Chemical Properties in the Interface, Related to Figure 4 mβ_2_m (left-hand side) and hβ_2_m (right-hand side) are shown as a surface representation colored according to their electrostatic potential (±2 K_B_T, where K_B_ is the Boltzmann constant and T is the temperature in Kelvin), with the BC, DE, and FG loops on the top. The ensemble of ΔΝ6 molecules around mβ_2_m and/or hβ_2_m is shown as green and yellow mesh, respectively. This representation is essentially the complementary picture of the ensembles shown in Figures 4C and 4D (where mβ_2_m and/or hβ_2_m are shown as weighted atomic probability density maps). Note the high correlation between the distribution of ΔΝ6 molecules around mβ_2_m with the hydrophobic surface of the latter. By contrast, part of the ΔΝ6 density map locates opposite the negatively charged part of the BC loop of hβ_2_m. The electrostatic surface potential was calculated using APBS (Baker et al., 2001), and movies were rendered in PyMOL (version 1.7rc1; Schrödinger, LLC).

Document S2. Article plus Supplemental Information
