# Supplementary material for: Visualization of Transient Protein-Protein Interactions that Promote or Inhibit Amyloid Assembly
Source: Mol Cell. 2014 Jul 17;55(2):214–26. doi: 10.1016/j.molcel.2014.05.026 (PMC4104025; doi:10.1016/j.molcel.2014.05.026)
Supplement: Document S1. Supplemental Experimental Procedures, Figures S1–S6, and Table S1 [file mmc1.pdf]

Molecular Cell, Volume *55*

Supplemental Information

Visualization of Transient Protein-Protein Interactions that Promote or Inhibit Amyloid Assembly

Theodoros K. Karamanos, Arnout P. Kalverda, Gary S. Thompson, and Sheena E. Radford

Supplemental Figures and Legends

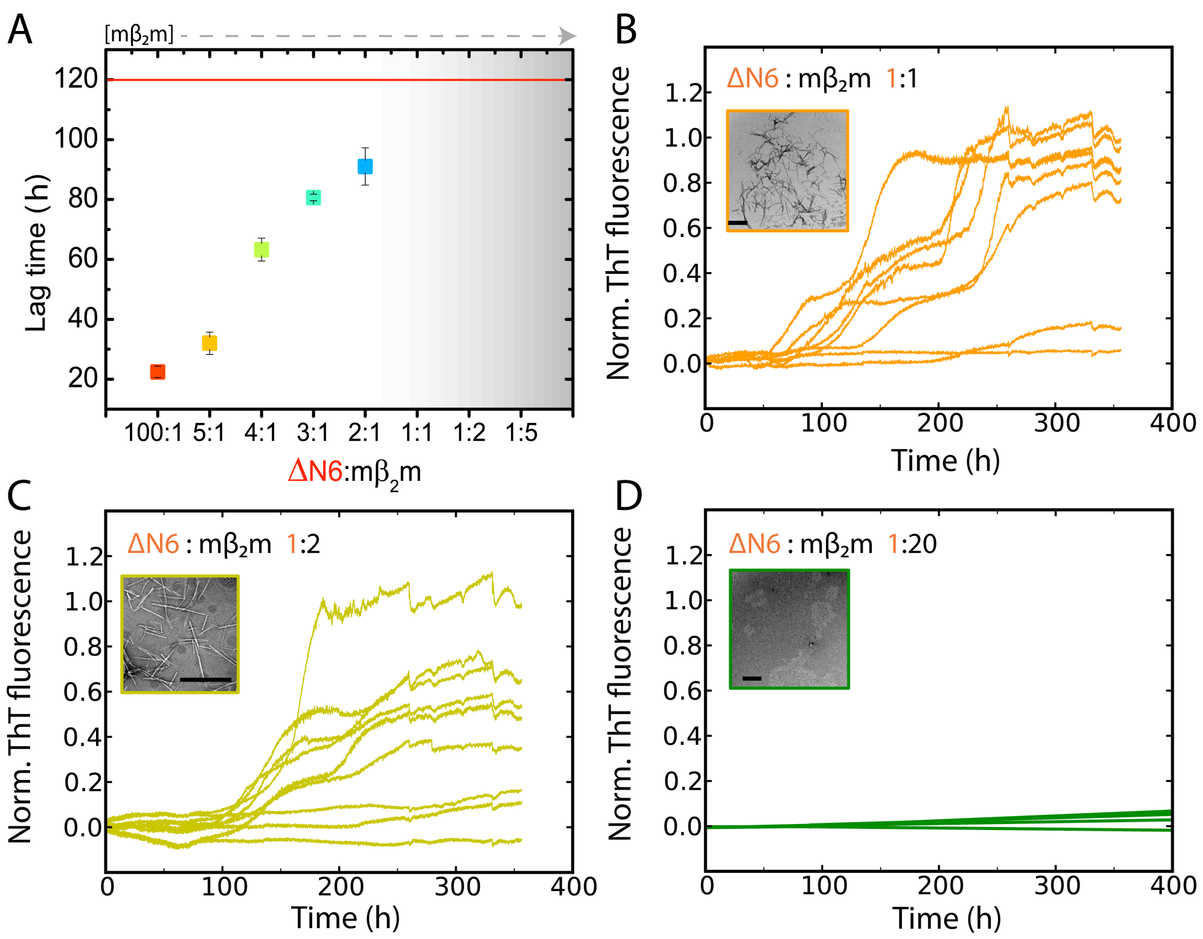

Figure S1 - related to Figure 2: Kinetic inhibition of ΔN6 amyloid formation by mβ_2m. (A) Plot of the average lag time of fibril formation for different molar ratios of ΔN6:mβ_2m. The grey-shaded area represents experiments where the lag time could not be estimated because the protein mixtures did not show an increase in ThT fluorescence by the end of the experiment shown in Figure 2A (120h-red line). Error bars represent the standard error of the mean. (B) Kinetics of amyloid assembly of mixtures of ΔN6:mβ_2m: 30µM:30µM, (C) 20µM:40µM and (D) 10µM:200µM over timescales up to 400h monitored using ThT fluorescence. Negative stain EM images of the fibrils formed at the end of each reaction are inset (scale bar = 500nm).

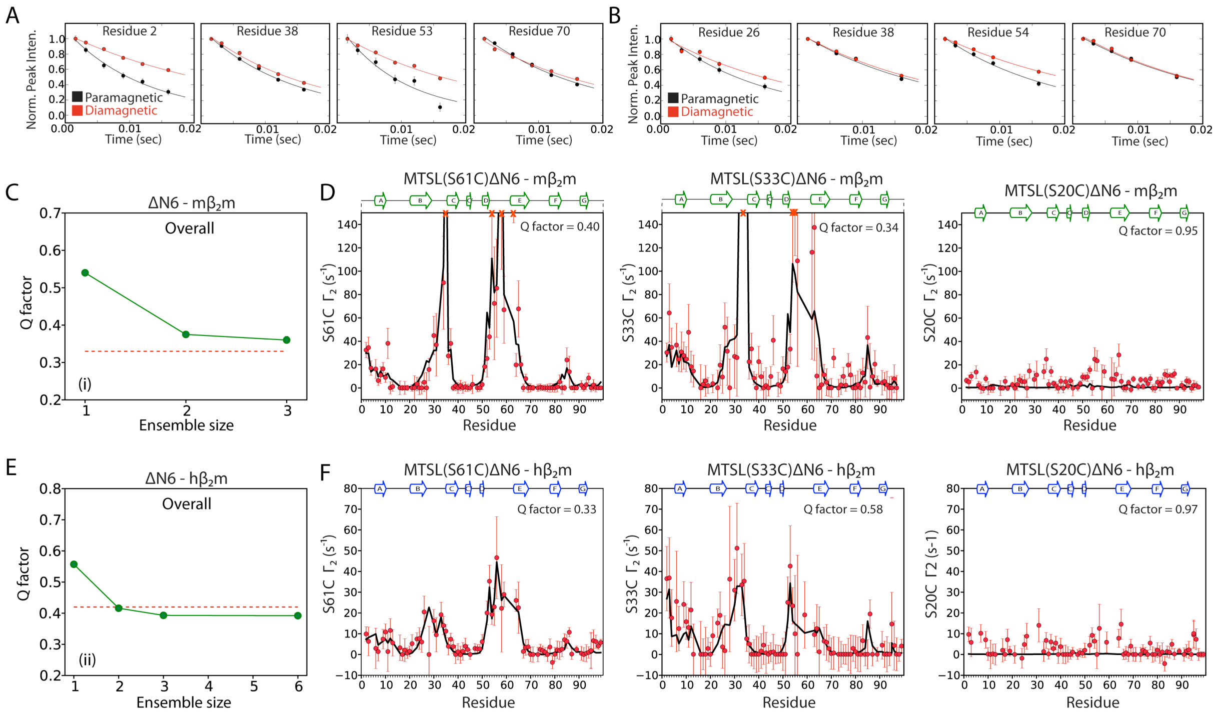

Figure S2 – related to Figure 4. Agreement between the experimental and back-calculated intermolecular PRE data for the ΔN6-mβ_2m and ΔN6-hβ_2m interactions. Intermolecular PRE profiles for the interaction between ^{14}N-ΔN6 spin labeled at position 61 and ^{15}N-labeled mβ_2m (A) or ^{15}N-labeled hβ_2m (B). (C) Plots of the calculated Q factor versus the number of ensemble members for the interaction of ΔN6 with mβ_2m. Dashed red lines represent the best Q factor possible for each dataset, if the quality of the fit is comparable to the error of the experimental data and is calculated as described in (Tang et al., 2008). (D) Experimental and calculated PRE data for the ΔN6-mβ_2m interaction when the spin label is attached at position 61 (left), 33 (middle) or 20 (right). Predicted PRE rates for an ensemble size of 2 (N=2) are shown in black lines while red dots denote the experimentally measured PRE rates. Data arising from the spin label on position 20 were not used in the fitting. The calculated Γ_2 rates shown represent the average values (per residue), back-calculated from 50 independent calculations (50x2 structures). All ensemble members were equally weighted during the calculations. (E) As in (C) and (F) as in (D) but for the association of ΔN6 with hβ_2m.

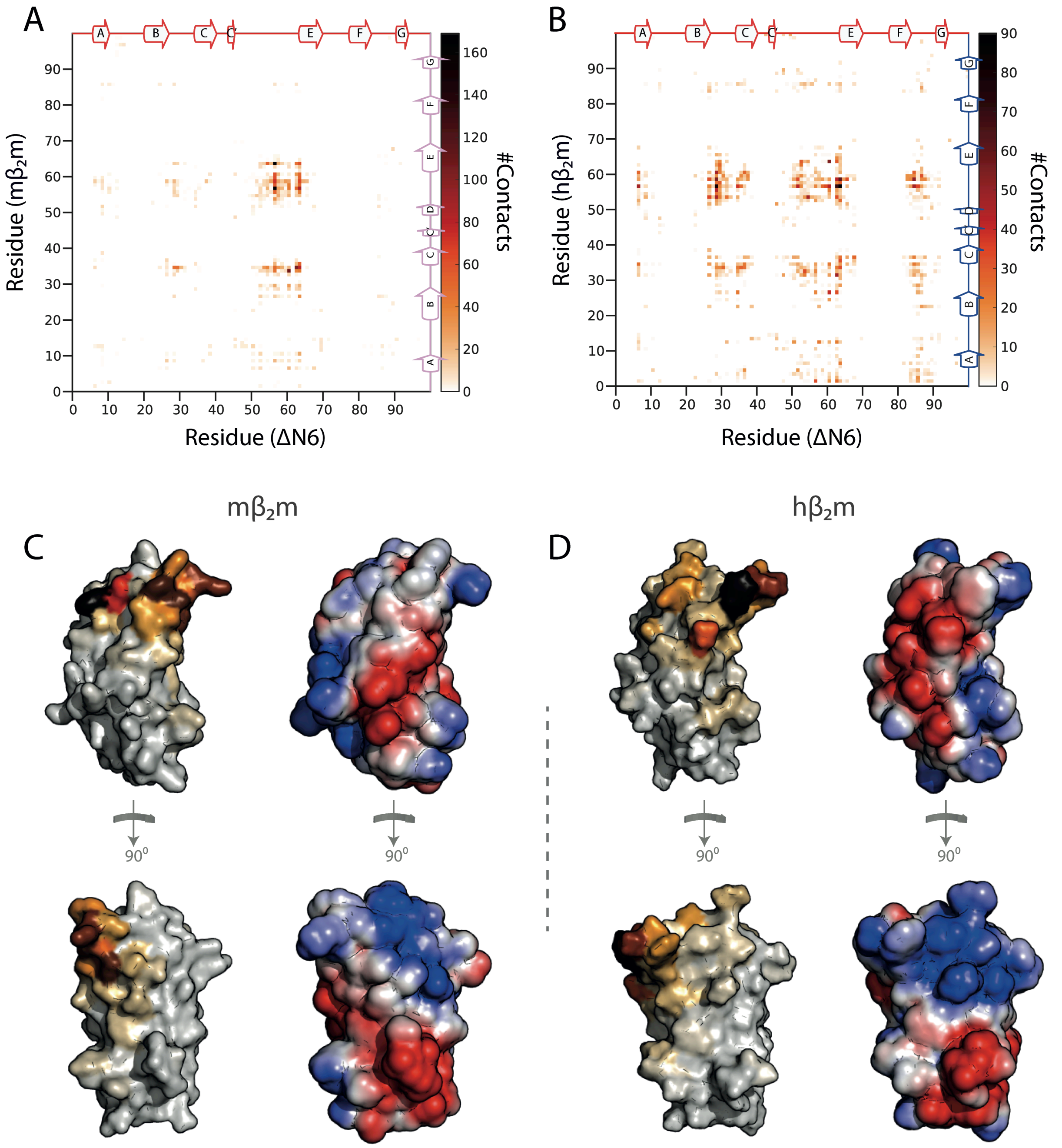

Figure S3 – related to Figure 4. Analysis of the ΔN6-mβ$_2$m and the ΔN6-hβ$_2$m interaction ensembles. (A) Contact map for the 50 best scoring ensembles for the ΔN6-mβ$_2$m or (B) the ΔN6-hβ$_2$m interaction. Every non-hydrogen atom with an intermolecular distance less than 4Å to any other (non-hydrogen) atom is identified as a contact. The number of contacts for atoms of each residue is color-coded as shown in the color-bar. (C) Structures mβ$_2$m colored according to the number of intermolecular contacts, using the same color scale as in (A) (left). A surface representation of the protein, colored according to its electrostatic potential (red

negative, blue positive, $\pm 2k_BT$) is shown in the right. (D) As in (C) but for hβ_2m. The pose of the proteins is the same in both cases, with the BC, DE and FG loops at the top of the molecule.

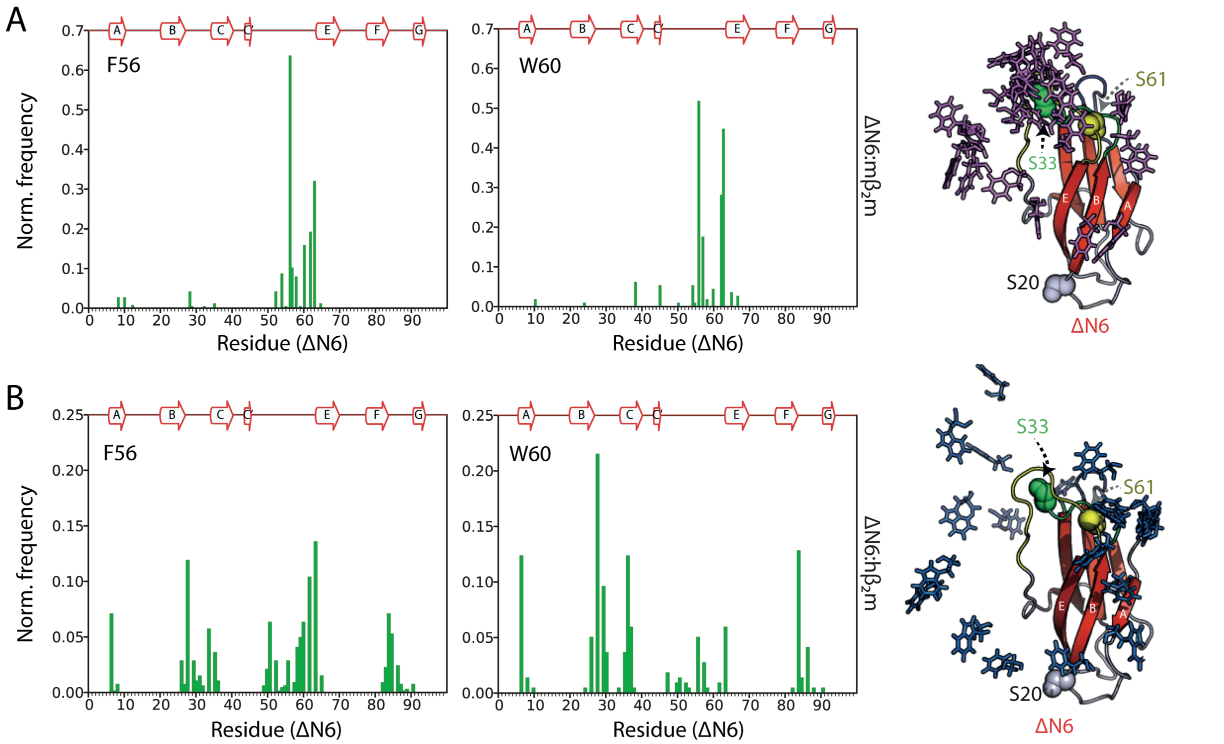

Figure S4 – related to Figure 5. The roles of F56 and W60 in the interface of different complexes. (A) Normalized frequency histograms of the number of contacts that F56 (left) or W60 (middle) of mβ_2m make with residues of ΔN6 in the ΔN6-mβ_2m complex. The 50 best-scoring ensembles (N=2, 50x2 structures) were analysed. These histograms essentially represent horizontal slices of the heat maps shown in Figure S3. The rightmost panel shows the position of F56 and W60 of mβ_2m (sticks) in the top 10 ensembles. ΔN6 is shown as red cartoon and the positions of the spin labels (S20, S33 and S61) are highlighted in spheres. (B) As in (A) but for the ΔN6-hβ_2m interaction. The distribution of F56 and W60 in the ΔN6-hβ_2m complex is much more diverse in comparison its ΔN6-mβ_2m counterpart. As a consequence, whilst the F56E/W60E mβ_2m mutant prevents the association of ΔN6 with mβ_2m, a more detailed mutational analysis in the interface of the ΔN6-hβ_2m complex is required to unpick the roles of specific residues in the course of assembly (Figure S4B).

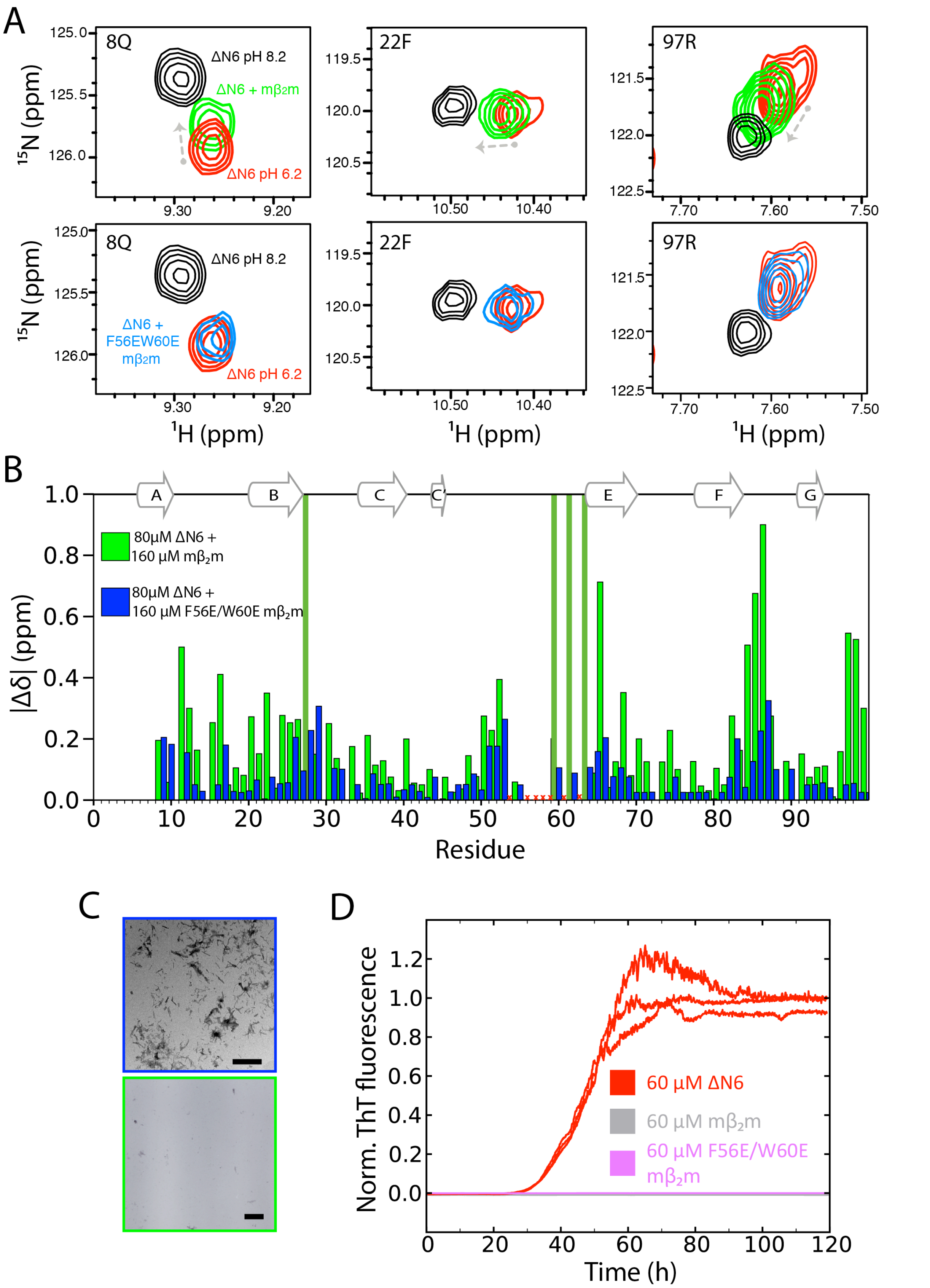

Figure S5 –related to Figure 5. Residual interactions between F56E/W60E mβ$_2$m and ΔN6 are not sufficient to inhibit fibrilation. (A) Additional examples of resonances of ^{15}N-labeled

ΔN6 (80μM, red) that show chemical shift changes upon the addition of ^{14}N-labeled mβ_2m (green), but not its F56E/W60E variant (160μM, blue) at pH6.2, 25°C. (B) Changes in the chemical shifts of 80μM ^{15}N-labelled ΔN6 upon addition of 160μM ^{14}N-labelled mβ_2m (green) or F56E/W60E mβ_2m (blue) (pH 6.2, 25ºC). Residues that are broadened beyond detection because of exchange line broadening in the spectrum of ΔN6:mβ_2m have an arbitrary value of 1. The small chemical shift differences observed in the F56E/W60E mβ_2m:ΔN6 sample suggest a residual interaction with increased K_d. (C) Negative stain electron micrograph of 20μM ΔN6 mixed with two molar equivalents of F56E/W60E mβ_2m (top) or wild-type mβ_2m (bottom). Bar represents 500nm. (D) Aggregation kinetics of ΔN6, F56E/W60E mβ_2m or wild-type mβ_2m (60μM each alone) followed by ThT fluorescence. Three example traces of each protein variant are shown.

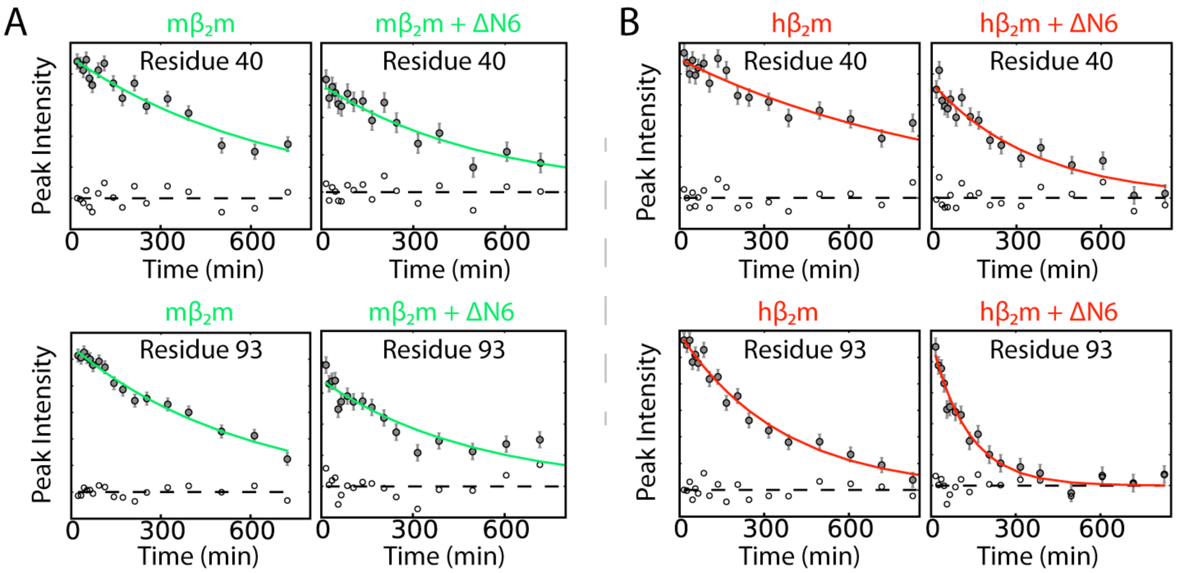

Figure S6 – related to Figure 6. H/D exchange rates of mβ_2m and hβ_2m upon interaction with ΔN6. (A) Plots of peak intensity versus time after the initiation of H/D exchange for example residues of ^{15}N-labeled mβ_2m alone (80μM, left column) or in the presence of 40μM ^{14}N-labeled ΔN6 (right column). Solid grey dots represent the raw data and solid lines show fits to single exponentials. Error bars were calculated from the noise level of the experiment. Open symbols show the residuals of the fits. (B) as in (A) but for ^{15}N-labeled hβ_2m alone (80μM) or in the presence of 160μM ^{14}N-labeled ΔN6.

Supplemental Movies

Movie S1, related to Figure 4. The mβ_2m-ΔN6 and hβ_2m-ΔN6 complexes involve different subunit orientations of a common head-to-head dimer. Movie animation of the structural ensembles shown in Figures 4C and 4D. ΔN6 is shown as cartoon representation with its BC loop highlighted in green, the DE loop in yellow and the FG loop in blue. The ensemble of mβ_2m molecules around ΔN6 is shown as a pink surface on the left hand side, while the hβ_2m ensemble is shown as a blue surface in the right had side.

Movie S2, related to Figure 4. The mβ_2m-ΔN6 and hβ_2m-ΔN6 complexes show different chemical properties in the interface. Mβ_2m (left hand side) and hβ_2m (right hand side) are shown as a surface representation coloured according to their electrostatic potential ($\pm 2k_BT$), with the BC, DE and FG loops on the top. The ensemble of ΔN6 molecules around mβ_2m and/or hβ_2m is shown as green and yellow mesh respectively. This representation is essentially the complementary picture of the ensembles shown in Figures 4C and 4D (where mβ_2m and/or hβ_2m were shown as weighted atomic probability density maps). Note the high correlation between the distribution of ΔN6 molecules around mβ_2m with the hydrophobic surface of the latter. By contrast, part of the ΔN6 density map locates opposite the negatively charged part of the BC loop of hβ_2m. The electrostatic surface potential was calculated using APBS (Baker et al., 2001) and movies were rendered in Pymol (Schrodinger, LLC, 2010).

Supplemental Tables

Table S1 – related to Figure 4. Analysis of the interfaces of different β_2m complexes. The buried surface area is calculated as the sum for the two subunits for each complex and is measured using XPLOR-NIH (Schwieters et al., 2003). Interface residues were identified as NACCESS (Hubbard and Thornton, 1993). Errors represent the standard deviation.

	**ΔN6 - mβ_2m complex**	**ΔN6 - hβ_2m complex**
Density volume (Å^3)	7,157	13,670
Buried surface area (Å^2)[1]	1481±290	1359±357
% Hydrophobic residues in the interface	48.6±14.5	43.81±12.6
% Charged residues in the interface	20.4±12.0	31.0±14.8

[1] Within each ensemble member

Supplemental Experimental Procedures

Protein preparation

Mβ_2m and F56A/W60A mβ_2m were purified using the protocol described in (Eichner et al., 2011), but the refolding buffer was adjusted to pH 8.5 and gel filtration was carried out in 10mM sodium phosphate, pH 8.2. S20C, S33C and S61C mutants of ΔN6 were refolded in 10mM TrisHCl buffer containing 0.64M L-arginine and/or 2mM reduced glutathione and/or 0.2mM oxidized glutathione before purification as in (Ladner et al., 2010).

Assembly of amyloid-like fibrils

Each experiment was repeated over at least 10 replicate samples and the median and/or mean lag time and standard error of the mean (SEM) were determined. Analysis of the soluble and insoluble material in each reaction was carried out at the end of fibril growth (120h or 350h) by collecting insoluble material by centrifugation (15.000*g*, 20min) and analysis by SDS-PAGE or ESI-MS (the latter subsequent to depolymerization by incubation for 10h in 100% *(v/v)* 1,1,1,3,3,3-hexafluoro-2-isopropanol (HFIP) (Sarell et al., 2013). Fibril morphology was analyzed using negative stain EM.

Negative-stain EM

Carbon coated copper grids were prepared by the application of a thin layer of formvar with an overlay of thin carbon. Samples were centrifuged (14,000*g*, 10min) and the pellets were resuspended in fresh 10mM sodium phosphate buffer, pH 6.2, diluted to a final protein concentration of 12μM with deionized water and then applied to the grid in a drop-wise fashion. The grid was then carefully dried with filter paper before it was negatively stained by

the addition of 18 µl of 2% (*w/v*) uranyl acetate. Micrographs were recorded on a Philips CM10 or a JEOL JEM-1400 electron microscope.

Analytical ultracentrifugation

For sedimentation velocity experiments, a total volume of 450µl sample in 10mM sodium phosphate buffer, pH 6.2, 83.3mM NaCl was inserted in standard double-sector Epon centerpieces equipped with sapphire windows, inserted in an An60 Ti four-cell rotor. Sample concentrations included 60µM or 120µM (ΔN6 alone), 60µM ΔN6 mixed with 60µM mβ_2m or 60µM F56E/W60E mβ_2m at pH 6.2. Absorbance data at 280nm were acquired at a rotor speed of 50,000rpm at 25°C. Data were analyzed using the c(s) continuous distribution of the Lamm equations with the software SEDFIT (Brown and Schuck, 2006).

$$D(s) = \frac{\sqrt{2}}{18\pi} kTs^{-1/2}(\eta(f/f_0)_w)^{-3/2}((1-\bar{v}\rho)/\bar{v})^{1/2} \qquad ,$$

where D(s) is the diffusion coefficient, k Boltzmann's constant, T the temperature in K, s the sedimentation coefficient, f is the frictional coefficient, f_0 the frictional coefficient of a compact smooth sphere, η the solvent viscosity, ρ represents the solvent density and $\bar{v}$ the partial specific volume.

Backbone assignments of mβ_2m

Backbone assignments for mβ_2m were obtained using triple resonance NMR techniques (HNCA, HNCO, HNCACB, HNCACO, HN(CO)CACB) and samples containing 500-750µM uniformly labeled $^{13}C/^{15}N$ mβ_2m in 10mM sodium phosphate buffer, pH 6.2, 83.3mM NaCl. Assignments are deposited in BMRB (19772).

PRE experiments

The ΔN6 variants (^{14}N-labeled) S20C, S33C and S61C (1-2mg/mL) were incubated with 5mM DTT for 20min, and then labeled immediately with MTSL by incubation with a 40-fold molar excess of the spin label for 4h in 25mM sodium phosphate buffer, pH 7.0, 1mM EDTA at room temperature. Excess spin label was removed by gel filtration (PD10 column, GE Healthcare). Spin-labeled ΔN6 was used directly or stored at -80°C. In all cases 100% labeling resulted at a single site as revealed by ESI-MS (not shown). For each PRE experiment MTSL-labeled ^{14}N-ΔN6 (10-60μM) was mixed with ^{15}N-labeled hβ_2m or mβ_2m (60-150μM) and the difference of the proton R_2 rates between oxidized and reduced (by addition of 1mM ascorbic acid) MTSL-labeled ^{14}N-ΔN6 was measured. Data were recorded at 25°C using a ^{1}H-^{15}N correlation based pulse sequence with 5-6 time-points (0.0016-0.016s) and at least 32 scans per incremental delay, utilizing a Varian-Inova 750MHz spectrometer equipped with a cryogenic probe. R_2 rates were extracted by fitting the relaxation data to single exponentials using in-house scripts. The H_N-Γ_2 rate was then calculated as the difference between the R_2 rate in the paramagnetic versus the diamagnetic sample:

$$\Gamma_2 = R_{2,para} - R_{2,dia} \quad .$$

Errors were calculated based on the noise of the experiment. The small PRE signal observed when ΔN6 is modified with MTSL at position 20 can be attributed to non-specific binding of the spin label itself, since addition of free MTSL results in a similar PRE profile. Thus, data arising from spin-labeled ΔN6 at position 20 were not included in quantitative analysis of the PRE experiments.

Simulated annealing calculations

Simulated annealing calculations were carried out in XPLOR-NIH (Schwieters et al., 2003). To account for the flexibility of the MTSL side chain, the paramagnetic group was

represented as a 5 membered ensemble. The agreement between the experimental and the back-calculated data is described by the PRE Q factor (Tang et al., 2006) defined as:

$$Q = \left[\sum i(\Gamma_{2,i}^{obs} - \Gamma_{2,i}^{cal})^2 / \sum i(\Gamma_{2,i}^{obs})^2 \right]^{1/2} ,$$

where $\Gamma_{2,i}{}^{obs}$ is the observed Γ_2 value for residue i and $\Gamma_{2,i}{}^{calc}$ is the calculated Γ_2 value. All calculations were started from randomized starting positions.

The computational strategy employed included two PRE potential terms (arising from S61C-ΔN6 and S33C-ΔN6) and classic geometry restraints to restrict deviation from bond lengths, angles and dihedrals. To generate a dataset suitable for this analysis, PREs arising from position 61 and 33 for the ΔN6-hβ_2m interaction were (each) measured in two independent experiments and the average PRE value for each residue was used for fitting. For the ΔN6-mβ_2m interaction, resonances in the BC and DE loop are not visible in the spectrum of the oxidized sample when the spin label is attached at positions 33 or 61 when the proteins are mixed in a 1:1 molar ratio. To obtain an estimate for the Γ_2 rate for these residues, the PRE experiments (using spin-labeled ΔN6 at positions 33 or 61) were repeated at different protein concentrations to: 1) improve the signal-to-noise ratio and 2) reduce the concentration of the bound complex so that a more accurate Γ_2 rate can be measured. PREs were then extrapolated to their values for a 1:1 complex using the measured K_d and the resulting dataset was used for quantitative analysis of the structural properties of the complex. Resonances for which an estimation of the R_2 rate in the presence of oxidized spin label was not possible, were incorporated in the protocol as nOe-type of restraints with an upper bound of 11.5Å and a lower bound of 9Å. Additionally, chemical shift perturbations observed upon binding were incorporated as sparse, highly ambiguous intermolecular distance restraints as described in (Clore and Schwieters, 2003). As chemical shifts can be influenced by numerous factors upon protein-protein interaction, the treatment of the derived data undertaken here results in a loose

potential term that is unlikely to bias the structure calculation. Finally, the protocol also included a weak radius of gyration restraint (R_{gyr}) calculated as $2.2N^{0.38}$, where N is the number of atoms in the complex. R_{gyr} is required in order to prevent bias towards more extended structures and tends to underestimate the true value of the radius of gyration (Kuszewski et al., 1999).

The aforementioned potential terms were used in a rigid-body energy minimization/ simulated annealing in torsion angle space protocol to minimize the difference between the observed and calculated Γ_2 rates, starting from random orientations. The first step in the structure calculation consisted of 5000 steps of energy minimization against only the sparse chemical shift restraints, followed by simulated annealing dynamics with all the potential terms active, where the temperature is slowly decreased (3000-25K) over 4fs. During the hot phase (T=3000K) the PRE and nOe terms were underweighted to allow the proteins to sample a large conformational space and they were geometrically increased during the cooling phase. Proteins were treated as rigid bodies until the initiation of the cooling phase, where side chains were allowed to float (semi-rigid body calculation). The final step included torsion angle minimization using all potential terms. Ensemble calculations where the interacting species are represented as multiple states (N>1) were carried out as before but in this case the Γ_2^{cal} is calculated as the average value between the conformers. The population of each conformer was set by specifying its weight in the calculation. For ensemble calculations the Q factor is calculated by averaging the predicted PRE value over all ensemble members corrected by their weight (the Q factor of the ensemble of ensembles).

In the case of the ΔN6-mβ_2m interaction when N=1, 7 out of 10 lowest energy structures share a backbone RMSD for the mβ_2m subunit of 4.4Å, suggesting that the complex shown in

Figure 5A represents the main associating species in solution. On the other hand, a single conformer representation (N=1) for the ΔN6-hβ_2m association yields a hβ_2m subunit RMSD of 20.5Å for the 10 lowest energy structures, an observation that also supports the larger conformational ensemble between ΔN6 and hβ_2m shown in Figure 4D. Buried surface area calculations were carried out in XPLOR-NIH (Schwieters et al., 2003).

Fitting K_d values

The total chemical shift differences as each ^{15}N-labeled protein was titrated with ^{14}N-labeled ΔN6 (pH 6.2, 25°C) was calculated using the function:

$$\Delta\delta_{tot} = \sqrt{(5 * \delta^1 H)^2 + (\delta^{15} N)^2}\ .$$

Residues for which the difference in chemical shift upon binding was ≥2 standard deviations from the mean were considered significant. These were used globally to extract the K_d using the function:

$$\Delta\delta = \Delta\delta_{\max} \frac{[L_T] + [U_T] + K_d - \sqrt{([L_T] + [U_T] + K_d)^2 - 4[L_T][U_T]}}{2[L_T]}\ ,$$

where [L_T], [U_T] are the total concentrations of the labeled and unlabeled protein added respectively, and $\Delta\delta_{max}$ represents the highest value of the chemical shift difference upon titration. A total of 10 and 8 residues were fitted for the ΔN6:mβ_2m and the ΔN6:hβ_2m interactions respectively. Four representative examples are shown in Figures 3C and 3D for clarity. Errors on the measured peak positions were calculated as the standard deviation of the mean for residues that show insignificant chemical shift changes. K_ds were extracted by Monte Carlo analysis with 1000 steps performed using in-house scripts.

Hydrogen exchange measurements

Samples for H/D exchange NMR were made in 10mM sodium phosphate pH 6.2 buffer and

freezed-dried. On the day of the experiment, samples were dissolved in 100% (*v/v*) D_2O containing 83.3mM NaCl and hydrogen exchange was measured using SOFAST-HMQC NMR methods (Schanda et al., 2005) utilizing a 750MHz Varian Inova spectrometer (Agilent) equipped with a cryogenic probe. The dead time of the experiment was 5-10min and each spectrum was acquired for 10-15min. H/D exchange rates for mβ_2m/hβ_2m were measured at 25ºC and 37ºC respectively. Rates were extracted by fitting single exponentials (Figure S6).

Supplemental References

Baker, N.A., Sept, D., Joseph, S., Holst, M.J., and McCammon, J.A. (2001). Electrostatics of nanosystems: Application to microtubules and the ribosome. Proc. Natl. Acad. Sci. USA *98*, 10037–10041.

Brown, P.H., and Schuck, P. (2006). Macromolecular size-and-shape distributions by sedimentation velocity analytical ultracentrifugation. Biophys. J. *90*, 4651–4661.

Clore, G.M., and Schwieters, C.D. (2003). Docking of protein–protein complexes on the basis of highly ambiguous intermolecular distance restraints derived from ^{1}H / ^{15}N chemical shift mapping and backbone ^{1}H - ^{15}N residual dipolar couplings using conjoined rigid body/torsion angle dynamics. J. Am. Chem. Soc. *125*, 2902–2912.

Eichner, T., Kalverda, A.P., Thompson, G.S., Homans, S.W., and Radford, S.E. (2011). Conformational conversion during amyloid formation at atomic resolution. Mol. Cell *41*, 161–172.

Hubbard, S.J., and Thornton, J.M. (1993). "NACCESS," computer program.

Kuszewski, J., Gronenborn, A., and Clore, M. (1999). Improving the packing and accuracy of NMR structures with a pseudopotential for the radius of gyration. J. Am. Chem. Soc. *121*, 2337–2338.

Ladner, C.L., Chen, M., Smith, D.P., Platt, G.W., Radford, S.E., and Langen, R. (2010). Stacked sets of parallel, in-register β-strands of β_2-microglobulin in amyloid fibrils revealed by site-directed spin labeling and chemical labeling. J. Biol. Chem. *285*, 17137–17147.

Sarell, C.J., Woods, L.A., Su, Y., Debelouchina, G.T., Ashcroft, A.E., Griffin, R.G., Stockley, P.G., and Radford, S.E. (2013). Expanding the repertoire of amyloid polymorphs by co-polymerization of related protein precursors. J. Biol. Chem. *288*, 7327–7337.

Schanda, P., Kupče, Ē., and Brutscher, B. (2005). SOFAST-HMQC experiments for recording two-dimensional heteronuclear correlation spectra of proteins within a few seconds. J. Biomol. NMR *33*, 199–211.

Schrodinger, LLC (2010). The PyMOL Molecular Graphics System, Version~1.7

Schwieters, C.D., Kuszewski, J.J., Tjandra, N., and Clore, G.M. (2003). The Xplor-NIH NMR molecular structure determination package. J. Magn. Reson. *160*, 65–73.

Tang, C., Ghirlando, R., and Clore, G.M. (2008). Visualization of transient ultra-weak protein self-association in solution using paramagnetic relaxation enhancement. J. Am. Chem. Soc. *130*, 4048–4056.

Tang, C., Iwahara, J., and Clore, G.M. (2006). Visualization of transient encounter complexes in protein-protein association. Nature *444*, 383–386.
