# Supplementary figures and images for: Visualization of Transient Protein-Protein Interactions that Promote or Inhibit Amyloid Assembly
Source: Mol Cell. 2014 Jul 17;55(2):214–26. doi: 10.1016/j.molcel.2014.05.026 (PMC4104025; doi:10.1016/j.molcel.2014.05.026)

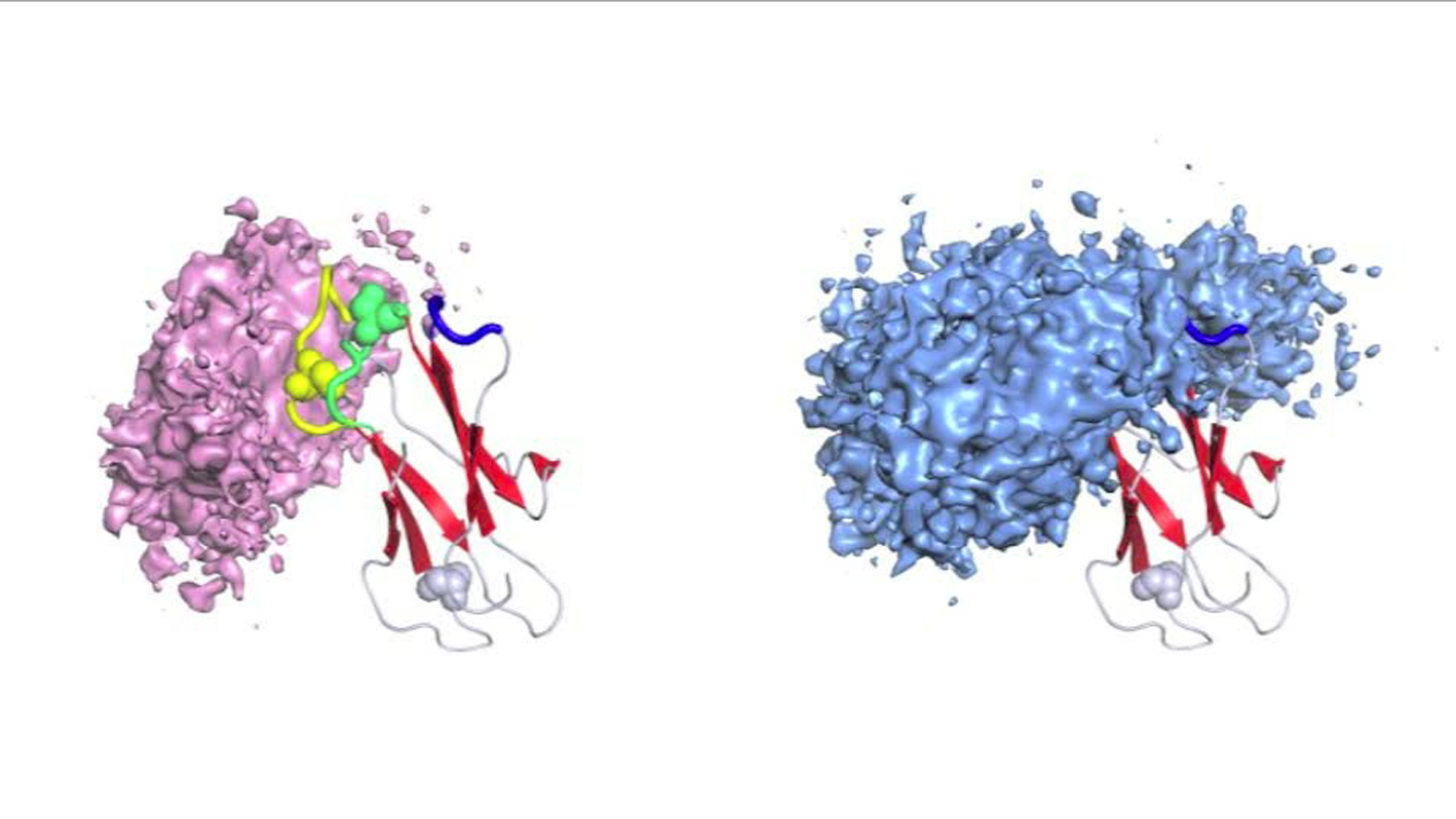

Supplement: Movie S1. The mβ2m-ΔN6 and hβ2m-ΔN6 Complexes Involve Different Subunit Orientations of a Common Head-to-Head Dimer, Related to Figure 4 — Movie animation of the structural ensembles shown in Figures 4C and 4D. ΔΝ6 is shown as a cartoon representation with its BC loop highlighted in green, the DE loop in yellow, and the FG loop in blue. The ensemble of mβ2m molecules around ΔΝ6 is shown as a pink surface on the left-hand side, whereas the hβ2m ensemble is shown as a blue surface on the right-hand side. [file mmc2.jpg]

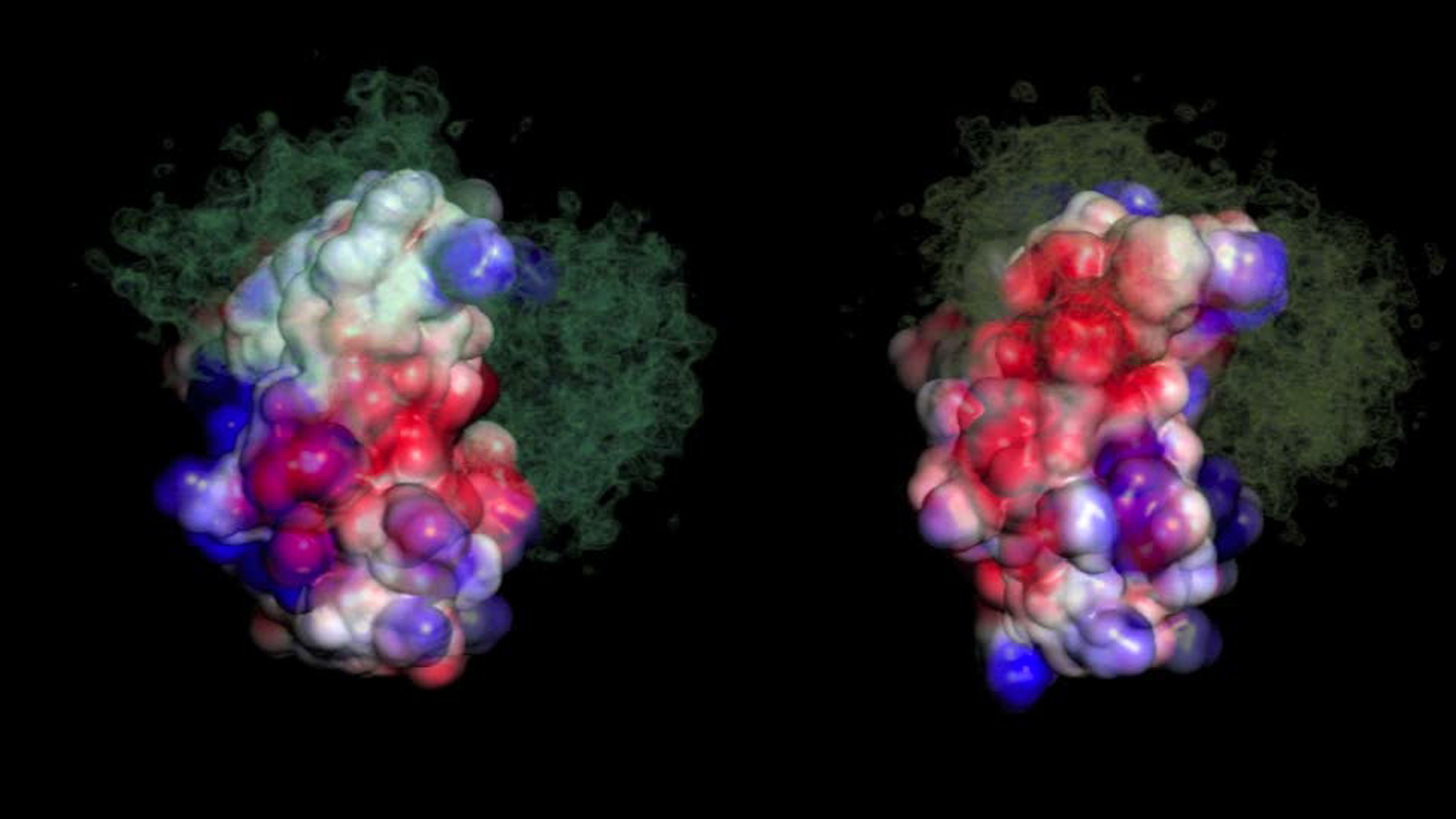

Supplement: Movie S2. The mβ2m-ΔN6 and hβ2m-ΔN6 Complexes Show Different Chemical Properties in the Interface, Related to Figure 4 — mβ2m (left-hand side) and hβ2m (right-hand side) are shown as a surface representation colored according to their electrostatic potential (±2 KBT, where KB is the Boltzmann constant and T is the temperature in Kelvin), with the BC, DE, and FG loops on the top. The ensemble of ΔΝ6 molecules around mβ2m and/or hβ2m is shown as green and yellow mesh, respectively. This representation is essentially the complementary picture of the ensembles shown in Figures 4C and 4D (where mβ2m and/or hβ2m are shown as weighted atomic probability density maps). Note the high correlation between the distribution of ΔΝ6 molecules around mβ2m with the hydrophobic surface of the latter. By contrast, part of the ΔΝ6 density map locates opposite the negatively charged part of the BC loop of hβ2m. The electrostatic surface potential was calculated using APBS (Baker et al., 2001), and movies were rendered in PyMOL (version 1.7rc1; Schrödinger, LLC). [file mmc3.jpg]
