# Supplementary material for: Visualization of Transient Protein-Protein Interactions that Promote or Inhibit Amyloid Assembly
Source: Mol Cell. 2014 Jul 17;55(2):214–26. doi: 10.1016/j.molcel.2014.05.026 (PMC4104025; doi:10.1016/j.molcel.2014.05.026)
Supplement: Document S2. Article plus Supplemental Information [file mmc4.pdf]

Molecular Cell
Article

Visualization of Transient Protein-Protein Interactions that Promote or Inhibit Amyloid Assembly

Theodoros K. Karamanos,[1] Arnout P. Kalverda,[1] Gary S. Thompson,[1] and Sheena E. Radford[1,*]
[1]Astbury Centre for Structural Molecular Biology and School of Molecular and Cellular Biology, University of Leeds, Leeds LS2 9JT, UK
*Correspondence: s.e.radford@leeds.ac.uk
http://dx.doi.org/10.1016/j.molcel.2014.05.026
This is an open access article under the CC BY license (http://creativecommons.org/licenses/by/3.0/).

SUMMARY

In the early stages of amyloid formation, heterogeneous populations of oligomeric species are generated, the affinity, specificity, and nature of which may promote, inhibit, or define the course of assembly. Despite the importance of the intermolecular interactions that initiate amyloid assembly, our understanding of these events remains poor. Here, using amyloidogenic and nonamyloidogenic variants of β_2-microglobulin, we identify the interactions that inhibit or promote fibril formation in atomic detail. The results reveal that different outcomes of assembly result from biomolecular interactions involving similar surfaces. Specifically, inhibition occurs via rigid body docking of monomers in a head-to-head orientation to form kinetically trapped dimers. By contrast, the promotion of fibrillation involves relatively weak protein association in a similar orientation, which results in conformational changes in the initially nonfibrillogenic partner. The results highlight the complexity of interactions early in amyloid assembly and reveal atomic-level information about species barriers in amyloid formation.

INTRODUCTION

The assembly of proteins into amyloid fibrils is a complex process requiring specific and sequence-dependent polymerization of initially unfolded or partially folded monomers into fibrils with elaborate cross-β architectures (Greenwald and Riek, 2010). Despite recent insights into the structural characteristics of the amyloid fold (Eisenberg and Jucker, 2012; Fitzpatrick et al., 2013), the process of amyloid assembly is less well understood in structural terms. Assembly from natively folded precursors is commonly initiated by partial unfolding (Chiti and Dobson, 2009). These nonnative species then combine, generating an array of oligomeric intermediates that are transiently populated and usually heterogeneous in mass and conformation (Cremades et al., 2012; Smith et al., 2010). Although recent advances in structural methods have enabled the conformational properties of rarely populated, partially folded monomers of aggregation-prone proteins to be determined (Eichner et al., 2011; Jahn et al., 2006; Neudecker et al., 2012), the nature of the first protein-protein interactions that initiate amyloid formation remains unclear. Early in assembly an array of biomolecular interactions arise, some of which may be productive for amyloid formation, whereas others may be unproductive, with the potential to inhibit or retard amyloid assembly (Campioni et al., 2010; Cremades et al., 2012). The course of amyloid assembly thus may depend on the stability and lifetime of the productive interactions versus their unproductive counterparts. From this viewpoint, identifying and characterizing different biomolecular interactions early in amyloid assembly are crucial for a full understanding of the structural, kinetic, and thermodynamic determinants of amyloid formation and for interpreting phenomena such as species barriers in prion formation. Such information could also pave the way toward the design of molecules able to define or control the course of amyloid assembly.

Amyloid formation is highly specific, with only proteins of closely related sequence capable of copolymerization into amyloid fibrils (Sarell et al., 2013a). Copolymerization may occur by cross-seeding, in which monomers of a different sequence are capable of extending preformed fibrils from a related protein (Giasson et al., 2003; Guo et al., 2013). In other cases, copolymerization may occur prior to the critical nucleation step of fibrillation. In this case, monomers or small oligomers coassemble into assembly-competent species in the early stages of amyloid assembly (Middleton et al., 2012; Sarell et al., 2013b). One such example can be found in prions, proteins that possess at least one conformation that is infectious by being able to transmit their structural and pathological properties onto noninfectious prion monomers (Chien and Weissman, 2001; Tessier and Lindquist, 2009). Interestingly, when prion molecules are transferred to different species they can lose their high infectivity, establishing a species barrier (Chien et al., 2003, 2004), or can confer their toxic conformation onto previously innocuous proteins of a related species (Sindi and Serio, 2009). The molecular determinants of species barriers, however, remain unclear.

Here we have explored the nature of protein-protein interactions in the first steps of amyloid formation of β_2-microglobulin (β_2m), a 99-residue protein that forms amyloid deposits in dialysis-related amyloidosis (DRA) (Gejyo et al., 1985). Despite being the main constituent of fibrils in DRA, wild-type human β_2m (hβ_2m) is not capable of forming amyloid-like fibrils on an experimentally tractable timescale in vitro at neutral pH without the addition of

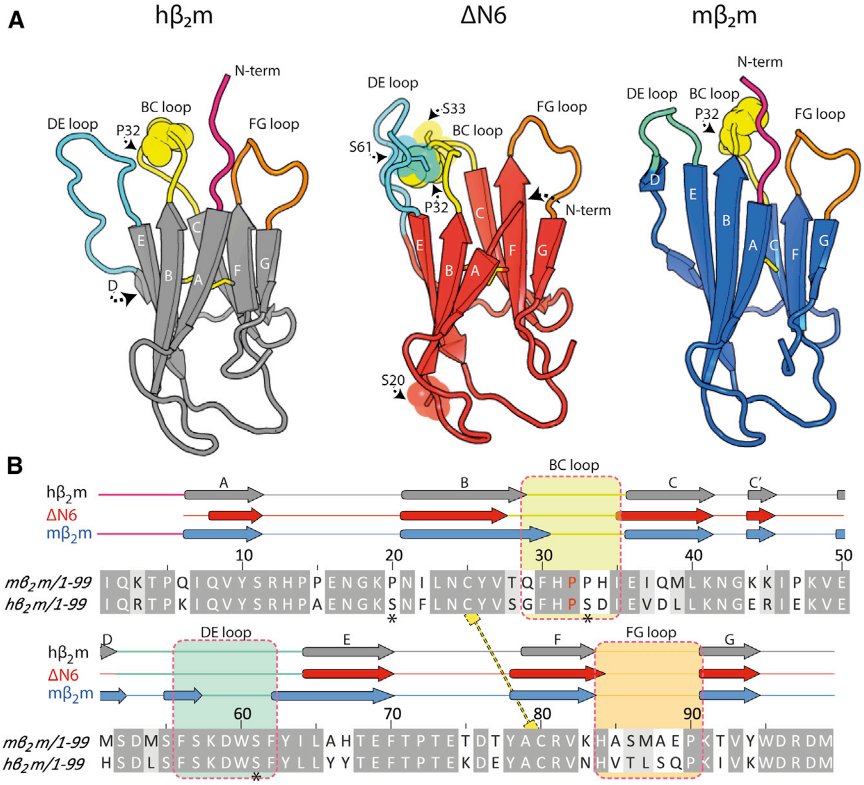

Figure 1. Comparison of β_2m Variants

(A) Structures of hβ_2m (Protein Data Bank [PDB] ID code 2XKS), ΔN6 (PDB ID code 2XKU) (Eichner et al., 2011), and mβ_2m (PDB ID code 1LK2) (Rudolph et al., 2004) (left to right).

(B) Secondary structure and sequence alignment of hβ_2m and mβ_2m. Regions identical in sequence are shown in gray. Regions in close spatial proximity to P32 (BC loop, DE loop, FG loop) are highlighted in the structures in (A) and with dashed boxes in (B). The disulfide bond is shown in yellow in (B). P32 is shown as yellow spheres in (A) and highlighted in red in (B). Positions of the spin labels are shown as spheres and sticks in the structure of ΔN6 in (A) and with an asterisk in (B).

external factors or cosolvents (Calabrese and Miranker, 2009; Eichner and Radford, 2011). By contrast, a truncated variant of β_2m in which the N-terminal six amino acids are deleted (ΔN6), a species that is found in amyloid fibrils in DRA (Esposito et al., 2000), is able to form amyloid fibrils spontaneously at neutral pH in vitro (Eichner et al., 2011; Esposito et al., 2000). NMR studies have shown this variant to be a close structural mimic of a folding intermediate of full-length hβ_2m that contains a nonnative *trans* X-prolyl bond at Pro32 (I_T) (Figure 1A), the formation of which has been shown to initiate aggregation (Eichner et al., 2011; Jahn et al., 2006). Importantly, ΔN6 is able to convert hβ_2m into an aggregation-competent state at neutral pH when added in a substoichiometric molar ratio (Figure 2A, inset), in a mechanism reminiscent of conformational conversion associated with prions (Eichner et al., 2011). By contrast with the behaviors of ΔN6 and hβ_2m, murine β_2m (mβ_2m), which is 70% identical in sequence to hβ_2m (Figure 1B), is unable to form amyloid fibrils at neutral pH (Eichner et al., 2011; Ivanova et al., 2004) and is capable of inhibiting the self-association of ΔN6 into amyloid fibrils when added in a stoichiometric ratio (Eichner et al., 2011).

Such diversity in the outcomes of interactions between β_2m molecules that are similar in sequence and structure provides an ideal system with which to study the principles of protein self-association in amyloid assembly and how different protein-protein interactions can lead to different molecular responses. Here we have combined the powers of different NMR approaches (paramagnetic relaxation enhancement [PRE] and chemical shift perturbation) with other biophysical and biochemical techniques to identify the molecular details of the protein-protein interactions that lead to the promotion (ΔN6-hβ_2m) or inhibition (ΔN6-mβ_2m) of fibril formation. The results reveal that the surfaces involved in the inhibition and promotion of fibrillation are similar. However, the spatial distribution and chemical properties of the generated ensembles differ in detail, sufficient to alter the affinities of these interactions and the effects of the biomolecular collision on the conformational properties of the monomeric precursors involved. Our findings highlight the complexity of the first steps in amyloid assembly, wherein protein association via similar binding surfaces results in different molecular outcomes. They also reveal information about the origins of species barriers in amyloid formation and identify the surfaces to target by molecular design to enable the course of amyloid assembly to be controlled and/or defined.

RESULTS

mβ_2m Kinetically Inhibits ΔN6 Assembly

In previous studies, we have shown that mβ_2m is able to inhibit the assembly of ΔN6 into amyloid-like fibrils when added in a stoichiometric ratio (Eichner et al., 2011), despite the high structural and sequence similarity of the two proteins (sequence identity 70%, sequence homology 90%, root-mean-square deviation 0.91 Å) (Figures 1A and 1B). This phenomenon was further investigated here by measuring the kinetics of fibril formation of ΔN6 at pH 6.2 (the pH optimum for ΔN6 fibril formation in vitro; Eichner et al., 2011) to which mβ_2m had been added in different molar ratios. To account for the effect of protein concentration on the kinetics of amyloid formation, the total protein concentration was maintained at 60 μM in all experiments. Figure 2A shows that ΔN6 assembles into fibrils able to bind thioflavin T (ThT) with lag-dependent kinetics typical of β_2m amyloid formation (Xue et al., 2008), whereas mβ_2m does not form fibrils under the conditions employed. Measured over more than ten replicates, the mean lag time of ΔN6 assembly was 32.7 ± 3.8 hr, after which time long straight fibrils typical of amyloid formed (Figure 2Bi). When mβ_2m was mixed with ΔN6 in substoichiometric molar ratios, 4:1 ΔN6:mβ_2m or 2:1 ΔN6:mβ_2m, the mean lag time

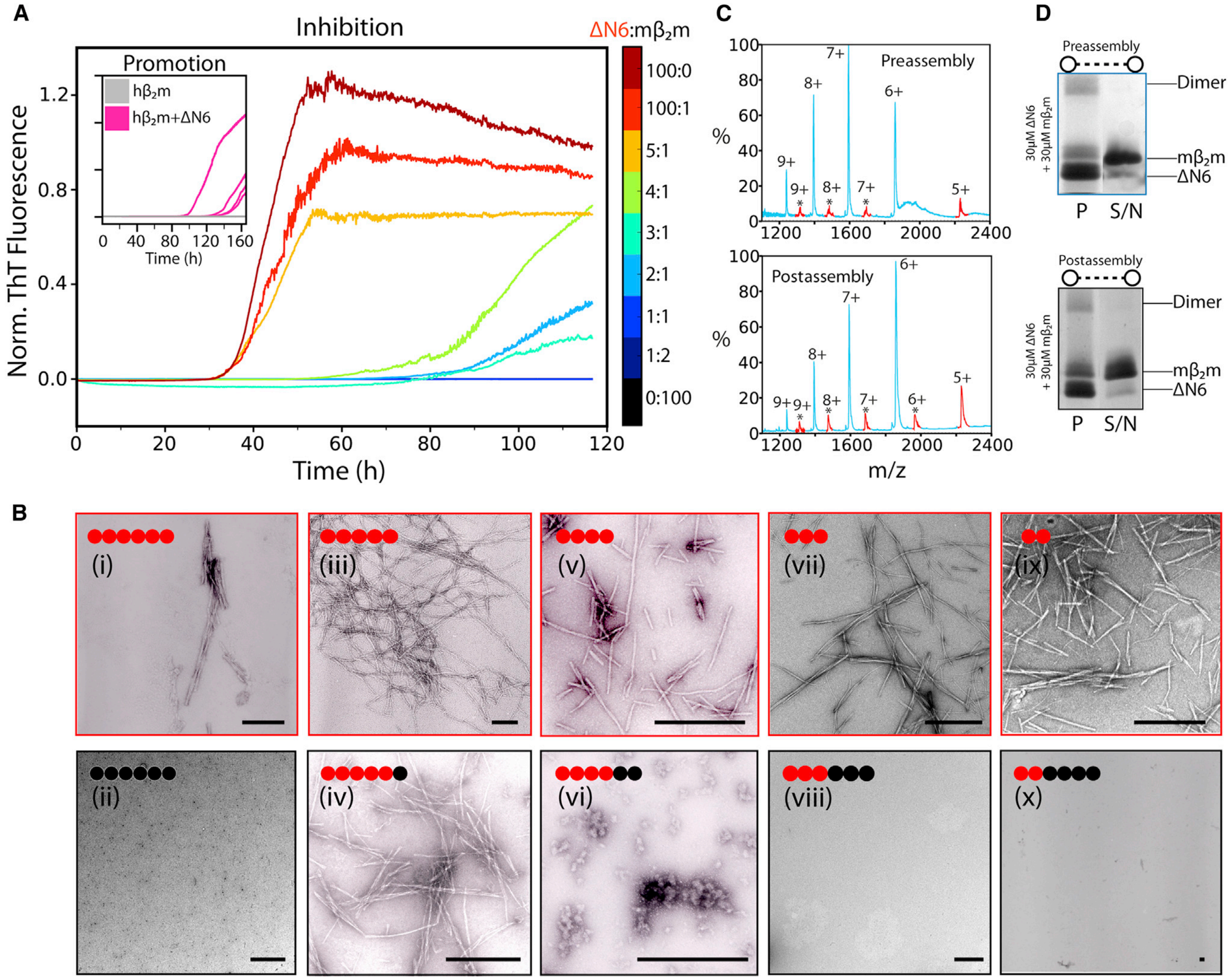

Figure 2. Inhibition of ΔN6 Fibril Formation by mβ_2m

(A) Aggregation kinetics of ΔN6 alone (dark red) or ΔN6 mixed with mβ_2m in different molar ratios (ΔN6:mβ_2m) measured using ThT fluorescence. The trace with the median lag time is shown. The total protein concentration for all samples is 60 μM. ThT traces of 60 μM hβ_2m alone (gray) or 59.4 μM hβ_2m mixed with 0.6 μM ΔN6 (pink; four replicates) are shown (inset).

(B) Negative-stain EM images of the endpoint of the reaction (after 120 hr) for sample traces in (A). Black spheres each represent 10 μM mβ_2m and red spheres represent 10 μM ΔN6. Scale bars represent 100 nm. ΔN6 alone, top row; mixtures of ΔN6:mβ_2m, bottom row.

(C) ESI mass spectrum of the pellet formed from 30 μM ΔN6 + 30 μM mβ_2m where the proteins were mixed either prior to fibril assembly (top) or subsequent to assembly (bottom). Fibrils formed after 350 hr of incubation (Figure S1) were pelleted by centrifugation, depolymerized in 100% (v/v) HFIP, and subjected to analysis by ESI-MS. Peaks corresponding to mβ_2m and ΔN6 are shown in red and blue, respectively.

(D) SDS-PAGE analysis of the samples shown in (C). P, pellet; S/N, signal to noise.

increased to 63.2 ± 3.8 and 91.0 ± 6.2 hr, respectively (Figure 2A), although fibrils formed over the 1 week (120 hr) time course of the experiment using a 5:1 molar ratio of the two proteins (Figure 2B). When the two proteins were mixed in a ≥1:1 molar ratio, complete inhibition ensued (Figure 2A). The dependence of the lag time on the concentration of mβ_2m added (Figure S1A available online) suggests that inhibition of fibrillation is a kinetically determined process. Accordingly, increasing the molar ratio of mβ_2m to ΔN6 delays, but does not inhibit, the formation of amyloid. In support of this notion, the mixtures of ΔN6:mβ_2m that did not show evidence of fibril formation after 120 hr were incubated for longer periods of time (≥350 hr) and the extent of fibril formation was again measured using ThT fluorescence and negative-stain electron microscopy (EM). These experiments showed that fibrils were able to form after extended incubation times, with the lag time depending on the excess of mβ_2m added (Figures S1B–S1D). These findings confirm that the interaction between ΔN6 and mβ_2m retards fibril assembly but, because fibrils are thermodynamically favored, the kinetic barrier to their formation is eventually overcome.

To identify whether mβ_2m is incorporated into fibrils when mixed with ΔN6, the aggregates formed in samples containing

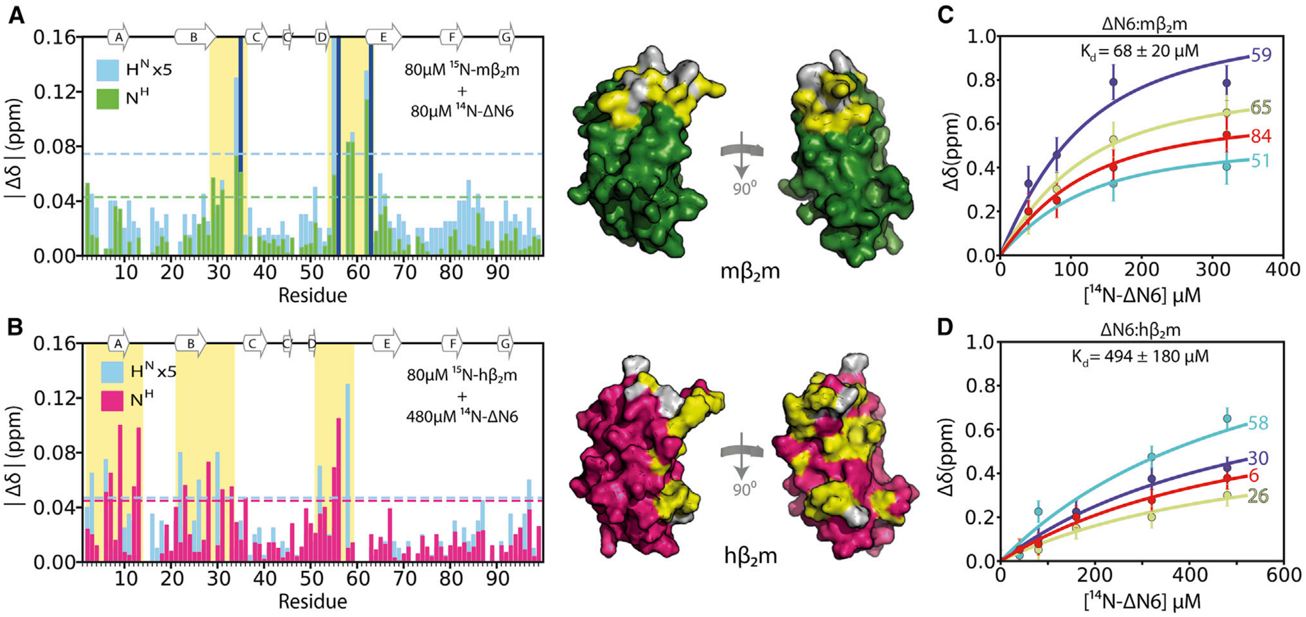

Figure 3. Chemical Shift Changes and Binding Affinities for Different Complexes

(A) Chemical shift differences (^{1}H, cyan; ^{15}N, green) when ^{15}N-labeled mβ$_2$m and ^{14}N-labeled ΔN6 are mixed in a 1:1 ratio (80 µM each; ~41% mβ$_2$m-bound). All residues experiencing significant chemical shift differences (yellow boxes) locate to the top half of the molecule (BC and DE loops; highlighted in yellow on the surface of the molecule; right-hand side). Residues that show large chemical shift differences in the presence of 40 µM ΔN6 but are broadened beyond detection at these protein concentrations are marked with dark blue bars.

(B) As in (A) but for ^{15}N-labeled hβ$_2$m and ^{14}N-labeled ΔN6 mixed in a 1:6 ratio (80 µM hβ$_2$m; 480 µM ΔN6; ~47% hβ$_2$m-bound). Residues with missing assignments are colored gray on the structure of mβ$_2$m/hβ$_2$m and have missing bars in (A) and (B). Dotted lines in (A) and (B) represent two standard deviations of the mean over the entire data set for each atom type.

(C) Plots of the chemical shifts of different residues (51, 59, 65, 84) in ^{15}N-labeled mβ$_2$m upon titration with ^{14}N-labeled ΔN6. Solid lines represent global fits to a binding hyperbola. Error bars were calculated using resonances known not to be involved in the binding interface. For these residues the chemical shift was measured in each spectrum, and the error bars represent the standard deviation of the mean of their peak positions (see Experimental Procedures and Supplemental Experimental Procedures).

(D) As in (C) but for ^{15}N-labeled hβ$_2$m upon titration with ^{14}N-labeled ΔN6. Curves for residues 6, 26, 30, and 58 are shown.

different molar ratios of ΔN6:mβ$_2$m after 350 hr were collected by centrifugation, depolymerized by incubation at 100°C in SDS-PAGE loading buffer or by incubation in 1,1,1,3,3,3-hexafluoro-2-isopropanol (HFIP), and subjected to analysis by SDS-PAGE or electrospray ionization mass spectrometry (ESI-MS) (Experimental Procedures; Supplemental Experimental Procedures). As a control, fibrils were assembled from ΔN6 alone, incubated subsequently with the same concentrations of monomeric mβ$_2$m, and analyzed in a similar manner. The results of these experiments showed that mβ$_2$m associates with the ΔN6 fibrils to a similar extent irrespective of whether the protein was added pre- or postassembly (Figures 2C and 2D). These results indicate that mβ$_2$m is not incorporated into the ΔN6 fibrils but associates with the fibril surface subsequent to assembly. By contrast, hβ$_2$m has been shown to be incorporated into the fibril core when incubated with ΔN6 in a 1:1 ratio at pH 6.2 (Sarell et al., 2013b).

Different Binding Affinities for the Inhibition and Promotion of Fibril Assembly

To investigate the interfaces involved in the inhibition (ΔN6-mβ$_2$m) or promotion (ΔN6-hβ$_2$m) of amyloid assembly, NMR studies were carried out by mixing ^{14}N-labeled ΔN6 with ^{15}N-labeled mβ$_2$m or hβ$_2$m (80 µM) and monitoring the chemical shift perturbations upon binding using ^{1}H-^{15}N HSQC spectra (Experimental Procedures). For both interaction types, the exchange was found to be in the intermediate-to-fast regime (data not shown), giving rise to small, but significant, chemical shift changes upon binding.

In the case of the inhibitory complex (ΔN6-mβ$_2$m), changes in the ^{1}H-^{15}N HSQC spectrum, including chemical shift differences and exchange line broadening, were observed for a subset of resonances, even when the proteins were mixed in substoichiometric ΔN6:mβ$_2$m ratios. Residues that show significantly altered chemical shifts upon binding are localized in the BC and DE loops in the apical region of mβ$_2$m (Figure 3A). By contrast, an excess (≥80 µM) of ^{14}N-labeled ΔN6 was required to observe significant chemical shift changes in the spectrum of ^{15}N-labeled hβ$_2$m (Figure 3B). In this case, the residues experiencing significant chemical shift differences include the N-terminal regions, the B strand, and the BC and DE loops (Figure 3B). Globally fitting the resulting data (Supplemental Experimental Procedures) yielded K_d values of 68 ± 20 µM for the mβ$_2$m-ΔN6 interaction and 494 ± 180 µM for the interaction between ΔN6 and hβ$_2$m (Figures 3C and 3D). Together, these data suggest a larger interface for the ΔN6-hβ$_2$m interaction (more

residues experience significant chemical shift perturbations) in comparison to its inhibitory ΔN6-mβ_2m counterpart, despite an ~7-fold decrease in binding affinity.

Inhibition and Promotion of Fibril Formation Involve Similar Binding Interfaces

Although chemical shifts are excellent probes of protein-protein interactions, they can be affected by long-range effects upon binding (Zhuravleva and Gierasch, 2011). Thus, we next sought to investigate the nature of the protein-protein interactions that lead to inhibition (ΔN6-mβ_2m) or promotion (ΔN6-hβ_2m) of fibril formation in more detail using PRE studies. The PRE depends on the distance between a paramagnet and adjacent nuclei and can provide long-distance (~30 Å) information quantified by the H_N-Γ_2 PRE rate (Supplemental Experimental Procedures) for each amide proton (Clore and Iwahara, 2009). The PRE approach is ideally suited to the analysis of weak intermolecular associations (Clore and Iwahara, 2009), providing distance information that can be used to visualize transient and lowly populated (<0.5%) protein states (Tang et al., 2006, 2008) such as those occurring in the early stages of amyloid formation. To enable these experiments, variants containing a solvent-exposed cysteine were created in ΔN6 by mutating either S20 (AB loop), S33 (BC loop), or S61 (DE loop) to cysteine (Figure 1A) while maintaining the disulfide bond involving C25 and C80 (Experimental Procedures). Chemical modification with (1-oxyl-2,2,5,5-tetramethyl-Δ3-pyrroline-3-methyl) methanethiosulfonate (MTSL) yielded ΔN6 molecules 100% labeled at a single site (Experimental Procedures). These chemically modified molecules were then used in PRE studies to map the interactions between ^{14}N-labeled and MTSL-labeled ΔN6 with ^{15}N-labeled hβ_2m or mβ_2m, each pair in a stoichiometric ratio (60 μM each) at pH 6.2 and 25°C (Experimental Procedures). Under the conditions employed, and in the absence of agitation, fibril formation does not occur for either pair of proteins over the time course of the experiment (<40 hr). Accordingly, the difference in the ^{1}H R_2 relaxation rates of the ^{15}N-labeled protein (hβ_2m/mβ_2m) in the presence of oxidized or reduced MTSL-labeled ^{14}N-ΔN6 was measured (H_N-Γ_2 rate) (Experimental Procedures) and used to map the interaction surfaces of the different protein pairs.

The PRE data collected for the inhibitory interaction between ^{14}N-labeled ΔN6 (S61C-MTSL) and ^{15}N-labeled mβ_2m are shown in Figure 4Ai and Figure S2A. Backbone assignments for mβ_2m at pH 6.2 were obtained using standard triple-resonance NMR experiments and uniformly ^{15}N/^{13}C-labeled protein (Experimental Procedures). The results showed high Γ_2 values (Γ_2 > 60 s^{-1}) for residues in the BC and DE loops of mβ_2m and lower Γ_2 values (<60 s^{-1}) for residues in the N-terminal 10 residues and the FG loop. These regions cluster on one side of mβ_2m surrounding P32 (Figure 1A; Figure 4A, inset), a residue that undergoes *cis-trans* isomerization known to be required for amyloid formation from hβ_2m (Eichner et al., 2011; Sakata et al., 2008). A similar PRE pattern was obtained when the spin label was attached at position 33 (Figure 4Aii). The results suggest that the region of mβ_2m surrounding P32 is involved in the interaction with ΔN6 to create a heterodimer (as supported by analytical ultracentrifugation; see below) that kinetically inhibits amyloid formation. Consistent with this supposition, when the spin label is moved to position 20 on ^{14}N-labeled ΔN6, the Γ_2 rates of mβ_2m in the BC and DE loops are substantially reduced (<25 s^{-1}) (Figure 4Aiii), suggesting that S20 is distant from the site of interaction (Supplemental Experimental Procedures). These data suggest, therefore, that a head-to-head configuration of the ΔN6-mβ_2m heterodimer, involving the BC and DE loops from both monomers, creates the inhibitory complex.

Having identified the protein-protein interactions that lead to the inhibition of ΔN6 fibril formation, we next investigated the interactions that lead to ΔN6-induced promotion of hβ_2m fibril assembly. Again, ^{14}N-labeled ΔN6 was spin labeled with MTSL at residues 61, 33, or 20 and PREs to ^{15}N-labeled hβ_2m were measured (Figure 4B; Figure S2B). In marked contrast with the results obtained for the ΔN6-mβ_2m interaction, the magnitude of the Γ_2 values is reduced significantly when the spin-labeled ΔN6 variants are mixed with hβ_2m (compare Figures 4Ai and 4Aii with Figures 4Bi and 4Bii), consistent with the ~7-fold lower K_d of the hβ_2m-ΔN6 complex (Figures 3C and 3D). Despite the differences in magnitude of the Γ_2 rates for the two complexes, the pattern of H_N-Γ_2 values obtained is similar to that for the ΔN6-mβ_2m interaction, with the largest PREs observed for residues 55–65 in the DE loop and 26–34 in the BC loop when the spin label is attached at position 61 (Figure 4Bi). Residues in the N-terminal region (residues 2–10) showed increased PRE rates when the spin label is attached at position 33, which were not observed when MTSL was added at position 61 (Figures 4Bi and 4Bii). Again, only very small PREs were observed when MTSL was added at position 20 (Figure 4Biii). These results suggest that the promotion of hβ_2m fibril formation also involves a head-to-head association of the two monomers.

Distinct Conformational Ensembles with Structurally Similar Binding Surfaces

To obtain more detailed insights into the protein complexes that give rise to the inhibition or promotion of amyloid formation, the PRE data were used in a rigid body/torsion angle simulated annealing approach to generate structural ensembles of the different complexes by minimizing the difference between the observed and calculated Γ_2 values. PRE data for each complex obtained using spin labels at positions 33 and 61 in ΔN6 were fitted simultaneously, along with data from chemical shift perturbations upon binding that were treated as ambiguous distance restraints (see below and Experimental Procedures). Data arising from spin-labeled ΔN6 at position 20 were not included (Supplemental Experimental Procedures). The population of the interconverting species was set to 18% in both cases based on the known K_ds of each complex.

In a first series of simulated annealing calculations, the interconverting species were represented as a single conformer (N = 1) (Experimental Procedures). The results of this analysis revealed a head-to-head configuration for the association of ΔN6 with mβ_2m in which the DE loops from each monomer make the majority of the intermolecular contacts (Figure 5A). Interestingly, the high Q factor (0.54; Figure S2C; Supplemental Experimental Procedures) suggests that multiple conformations are required to satisfy the experimental restraints. In exchanging

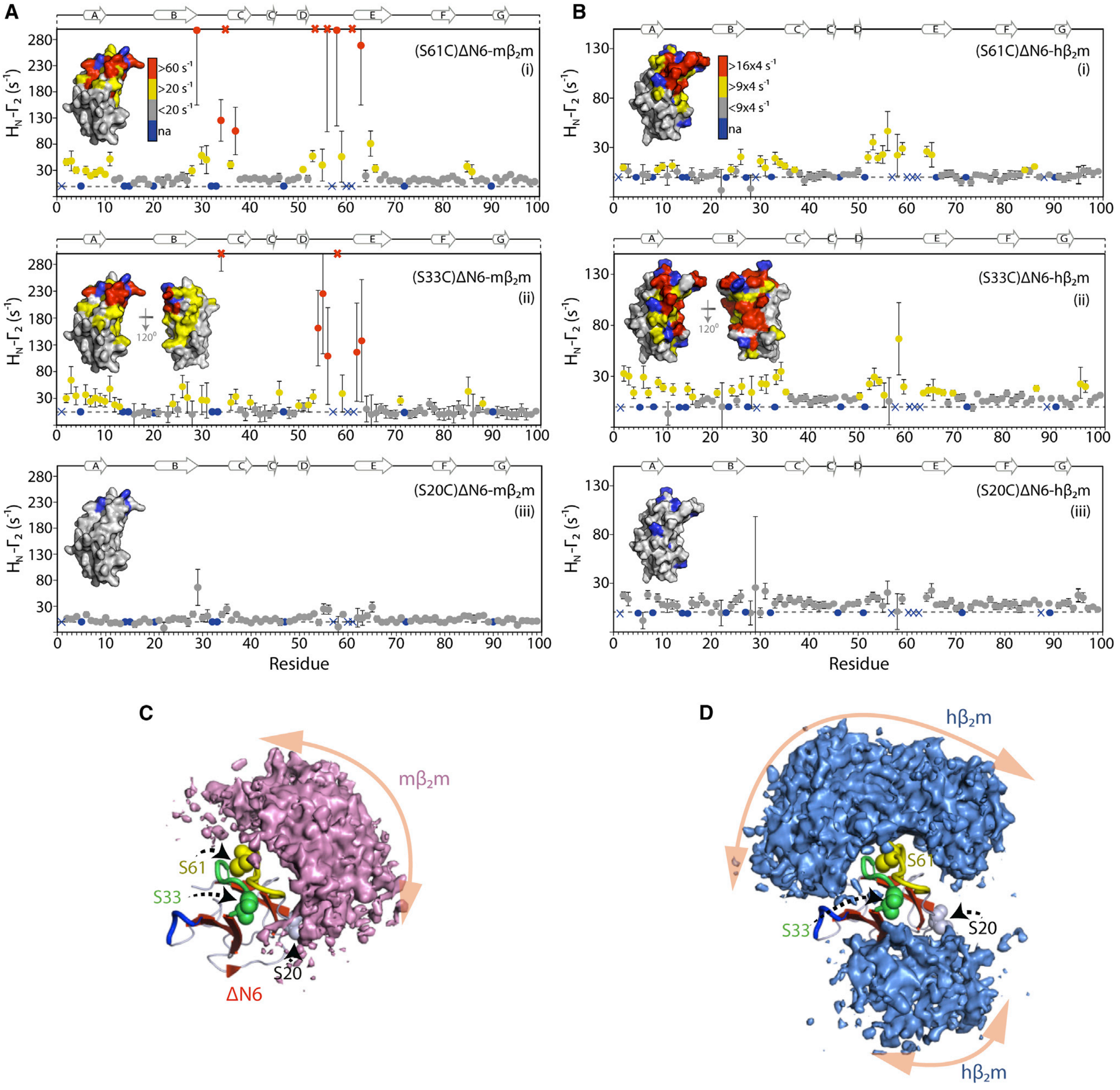

Figure 4. Interaction Interfaces in Different Protein Complexes

(A) Per-residue Γ_2 rates of $m\beta_2m$ (60 μM) when MTSL is attached to S61 (i), S33 (ii), or S20 (iii) on ΔN6 (60 μM) colored according to their amplitude (blue, not assigned; gray, insignificant; yellow, >20 s^{-1}; red, >60 s^{-1}; pH 6.2, 25°C). The structure of $m\beta_2m$ as a surface representation colored by the amplitude of the Γ_2 rates is shown (insets). Red crosses indicate residues for which the Γ_2 rate is either too large to appear on this scale or resonances broadened beyond detection when the spin label is oxidized and hence the Γ_2 rate cannot be measured. Blue dots represent proline or overlapping resonances, and blue crosses denote residues for which the assignments are missing. Error bars were calculated from the noise level in the experiment.

(B) As in (A) but for the interaction between ^{14}N- and MTSL-labeled ΔN6 (60 μM) and ^{15}N-labeled $h\beta_2m$ (60 μM). The structure of $h\beta_2m$ is colored according to the amplitude of the Γ_2 rates after extrapolation to the same % bound as in (A) (blue, not assigned; gray, insignificant; yellow, >9 × 4 s^{-1}; red, >16 × 4 s^{-1}). Note that the scale is expanded in (B).

(C) The distribution of the $m\beta_2m$ molecules in the ΔN6-$m\beta_2m$ complex, with the $m\beta_2m$ ensemble shown as a pink surface around ΔN6 (cartoon). The 50 top-scoring ensembles (N = 2, 2 × 50 structures) were included in the calculation.

(D) As in (C) but for the ΔN6-$h\beta_2m$ association. The pose of ΔN6 is identical to (C) and the ensemble of $h\beta_2m$ subunits is colored in blue. The BC, DE, and FG loops of ΔN6 are highlighted in green, yellow, and blue, respectively, and the positions of the spin label (S20, S33, and S61) are shown as spheres.

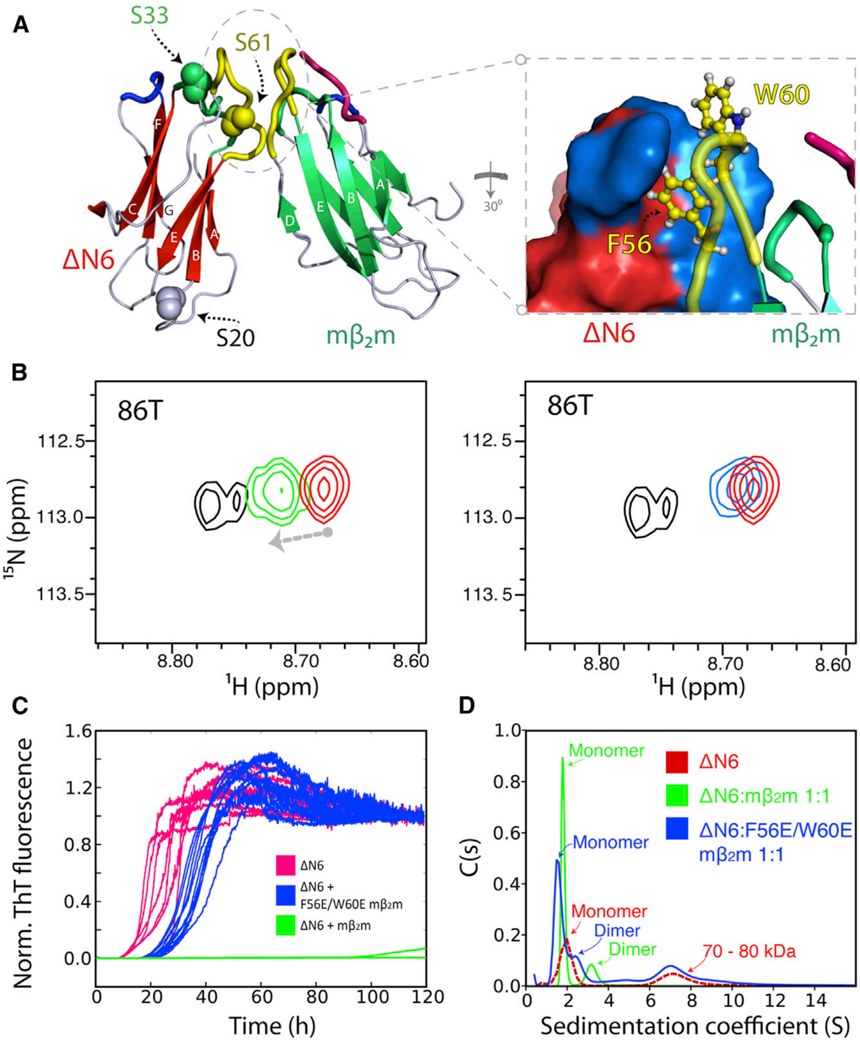

Figure 5. F56 and W60 in mβ$_2$m Form Interactions Required for Amyloid Inhibition

(A) The lowest-energy calculated structure of the ΔN6 (red)-mβ$_2$m (green) complex highlighting F56 and W60 in the interface. Interface residues are colored blue on ΔN6 (right).

(B) Representative sample resonances in the ^{1}H-^{15}N HSQC spectrum of ^{15}N-labeled ΔN6 (80 μM; red) that show chemical shift changes upon the addition of ^{14}N-labeled mβ$_2$m (green) but not its F56E/W60E variant (160 μM; blue). Addition of mβ$_2$m shifts the resonances of ΔN6 toward their positions at pH 8.2 (black), where ΔN6 is not amyloidogenic (Eichner et al., 2011) (additional examples are shown in Figure S5A).

(C) Fibrillation kinetics of ΔN6 alone (20 μM; pink) at pH 6.2 and in the presence of a 2-fold molar excess of mβ$_2$m (green) or F56E/W60E mβ$_2$m (blue).

(D) Sedimentation velocity AUC traces of ΔN6 alone (60 μM; red), ΔN6 (60 μM) mixed with an equimolar concentration of mβ$_2$m (green), or F56E/W60E mβ$_2$m (blue).

systems the observed PRE rate is the weighted population average of the species in solution, as long as those are in the fast exchange regime (Iwahara et al., 2004). In this case, the PRE methodology allows the visualization of the ensemble of the interconverting species. Increasing the number of conformers to two (N = 2) results in a significant decrease in the Q factor for the ΔN6-mβ$_2$m interaction (Q = 0.37), with no further significant decrease (Q = 0.36) when N is increased to three (Figures S2C and S2D). Similar analysis of the ΔN6-hβ$_2$m association revealed that (at least) two conformers are also required to describe the experimentally measured PRE data (Figures S2E and S2F).

The associating monomers in the ΔN6-mβ$_2$m and ΔN6-hβ$_2$m ensembles were visualized as atomic probability density maps as described (Tang et al., 2006) (Figures 4C and 4D). The resulting ensemble for the ΔN6-mβ$_2$m complex shows that mβ$_2$m molecules cluster around the DE loop of ΔN6 (residues 52–63), which makes the majority of contacts with mβ$_2$m (Figure 4C; Figure S3A; Movie S1). ΔN6, by contrast, shows a bimodal distribution around the DE loop of mβ$_2$m, with one cluster of molecules facing the β sheet composed of the A, B, E, and D strands, whereas the second cluster of ΔN6 molecules locates opposite the edge strands D and C (Movie S2). On the other hand, the ΔN6-hβ$_2$m interaction is more heterogeneous, extending to both sides of the apical region of ΔN6 (around P32) (Figure 4D; Figure S3B; Movie S1). The volume of the ΔN6-mβ$_2$m density map is calculated to be 7,157 Å^3, whereas that of the ΔN6-hβ$_2$m cluster is almost twice as large (13,670 Å^3; a cutoff of 40% was used in both cases; Table S1). Interestingly, the distributions of mβ$_2$m and hβ$_2$m molecules around ΔN6 do not completely overlay. Areas showing high intermolecular contacts unique to the ΔN6-hβ$_2$m complex involve the BC and FG loops of ΔN6 interacting with the BC and DE loops of hβ$_2$m (Figure S3B). A correlation between the hydrophobic surface area of mβ$_2$m (shaped mainly by the region surrounding the DE loop) and the distribution of ΔN6 molecules is observed, indicating that this interaction interface is predominantly hydrophobic in nature, with residues F56, W60, and F62 participating in key intermolecular contacts (Figure S3C; Movie S2). By contrast, the apical region of hβ$_2$m (DE, BC, and FG loops) displays less solvent-exposed hydrophobic surface area and a greater predominance of charged residues that reflect the differences in the sequence of the proteins in these regions (Figure 1; Figure S3D; Table S1; Movie S2). Together, the results indicate that inhibition of ΔN6 fibril formation involves a "specific" head-to-head protein association driven by hydrophobic interactions with mβ$_2$m. On the other hand, the ΔN6-hβ$_2$m interaction, although also adopting a head-to-head configuration, is weaker, more heterogeneous, and involves electrostatic interactions. Whether these data reflect the formation of a range of "encounter complexes"

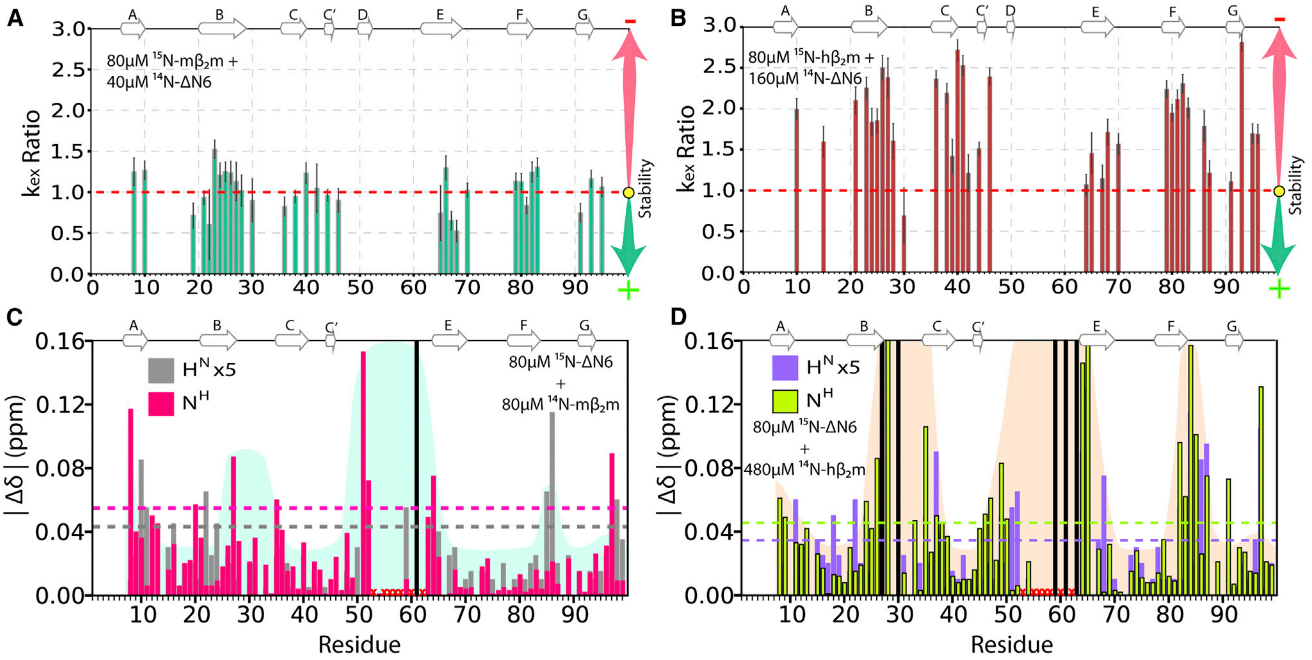

Figure 6. Dynamic versus Rigid Body Interactions in Different Protein Complexes

(A) The ratio of the H/D exchange rates of ^{15}N-labeled mβ_2m bound (~22%) to unlabeled ΔN6 (80 μM mβ_2m + 40 μM ΔN6) versus free (80 μM) ^{15}N-labeled mβ_2m (k_{ex} ratio, bound:free) plotted against residue number. An increase in k_{ex} ratio indicates a loss of H/D exchange protection upon binding. Error bars represent the propagated error of the fits to the raw data shown in Figure S6.

(B) As in (A) but for free (80 μM) ^{15}N-labeled hβ_2m versus ~22% ^{15}N-labeled hβ_2m bound to ΔN6 (80 μM hβ_2m + 160 μM ΔN6). Note that exchange of hβ_2m occurs by a mixed EX1/EX2 mechanism (Hodkinson et al., 2009), ruling out analysis of these data in terms of the free energy of binding.

(C) Differences in ^{1}H (gray) and ^{15}N (red) chemical shifts when ^{15}N-labeled ΔN6 and ^{14}N-labeled mβ_2m are mixed in a 1:1 ratio (80 μM each; ~45% ΔN6-bound). Dotted lines represent two standard deviations of the mean over the entire data set for each nucleus.

(D) As in (C) but for ^{15}N-labeled ΔN6 mixed with ^{14}N-labeled hβ_2m (80 μM hβ_2m + 480 μM ΔN6; ~45% ΔN6-bound). Black bars denote residues that are broadened beyond detection but show significant chemical shift changes when less mβ_2m/hβ_2m is added. Red crosses denote ΔN6 residues that are broadened at pH 6.2. Residues that experience significant chemical shift changes on binding are highlighted in blue and pink backgrounds in (C) and (D), respectively.

between ΔN6 and hβ_2m that is not observed for the ΔN6-mβ_2m interaction, or whether they report on the transient formation of higher-order oligomers between ΔN6 and hβ_2m, remains to be resolved.

Mutation of Aromatic Residues Prevents Inhibition of ΔN6 Assembly by mβ_2m

To confirm that the head-to-head association of ΔN6 with mβ_2m is involved in inhibition of fibril formation, two amino acid substitutions (F56E and W60E) were introduced into mβ_2m at sites that were found to participate in the majority of intermolecular contacts between the two molecules (Figure 5A; Figure S4A). The ability of this variant to bind to ΔN6 and to inhibit fibril assembly was then monitored using NMR and ThT fluorescence assays, respectively. When ^{14}N-labeled F56E/W60E mβ_2m (160 μM) was mixed with ^{15}N-labeled ΔN6 (80 μM) at pH 6.2, only small changes in the chemical shifts of ΔN6 (~20% in comparison to wild-type mβ_2m) were observed in the BC, DE, and FG loops (Figure 5B; Figures S5A and S5B), consistent with the proteins no longer interacting tightly. Consistent with these observations, F56E/W60E mβ_2m is unable to inhibit ΔN6 fibril assembly when added in a 2-fold molar excess (Figure 5C; Figures S5C and S5D), conditions under which wild-type mβ_2m delays the onset of amyloid for more than 120 hr (Figure 2A; Figure S1C). The interaction of wild-type mβ_2m with ΔN6 prevents the formation of oligomeric species by the latter protein as observed by sedimentation velocity analytical ultracentrifugation (AUC) (Figure 5D), resulting in a monomer-dimer (~80:20) equilibrium, consistent with a specific interaction as suggested by the analysis of the PRE data. Notably, under identical conditions, the F56E/W60E variant abolishes the ability of the murine protein to dissociate preformed oligomers of ΔN6 (Figure 5D).

Binding-Induced Unfolding versus Rigid Body Docking: A Rationale for the Outcome of Biomolecular Collision

To investigate why biomolecular collision of hβ_2m or mβ_2m with ΔN6 results in different outcomes of assembly, the effect of ΔN6 binding on the conformational dynamics of each monomer was measured using hydrogen-deuterium (H/D) exchange. In each case, the rate of H/D exchange of monomeric (unbound) hβ_2m/mβ_2m was compared with its ΔN6-bound counterpart at pH 6.2, using samples in which the protein concentrations of hβ_2m/mβ_2m were adjusted to generate complexes containing a similar percent (~20%) of ΔN6-bound hβ_2m/mβ_2m monomer. These experiments showed that the (H/D) exchange rates of mβ_2m are unaffected (k_{ex} increases by less than ~1.3-fold)

upon interaction with ΔN6 (Figure 6A; Figure S6A). By contrast, the addition of ΔN6 to hβ_2m causes a 2- to 3-fold increase in the H/D exchange rates of residues throughout the sequence of hβ_2m (Figure 6B; Figure S6B), consistent with an increase in global dynamics of the protein upon interaction with ΔN6. These results were confirmed using a variety of ΔN6 concentrations for both complexes, ranging from 40 to 320 μM.

Close examination of the chemical shift changes that occur when ^{14}N-labeled ΔN6 is added to ^{15}N-labeled mβ_2m reveals that the residues that undergo significant chemical shift changes also experience increased PRE rates (BC and DE loops), confirming that these regions of the protein form the interaction interface (Figure 3A). On the other hand, residues in the N-terminal region including the AB loop of hβ_2m (residues 12–13) show significant chemical shift changes upon binding to ΔN6 (Figure 3B) but minor PREs (Figure 4B), consistent with these residues not being involved in the interface of the lowest-energy structures of the ΔN6-hβ_2m complex (Figure S3B). These observations suggest that the binding of ΔN6 to hβ_2m provides sufficient energy to alter the conformation of the N-terminal 12 residues of hβ_2m (observed previously by H/D exchange and relaxation NMR methods; Eichner et al., 2011) such that a more amyloidogenic conformation is adopted. By contrast, the nonamyloidogenic (and thermodynamically less stable) mβ_2m ($\Delta G_{un}^{\circ}{}_{mouse} = -10.7$ kJ/mol, $\Delta G_{un}^{\circ}{}_{human} = -22.5$ kJ/mol; C. Pashley and S.E.R., unpublished data) is not affected significantly by binding. Differences in cooperativity or local stability of the interacting monomers thus dictate the progress of amyloid assembly.

Finally, the consequences of binding on the conformational properties of ΔN6 were investigated by measuring the changes in the chemical shifts of ^{15}N-labeled ΔN6 (80 μM) upon titration with ^{14}N-labeled mβ_2m (80 μM) or ^{14}N-labeled hβ_2m (480 μM) (~45% ΔN6 bound in each case) (Figures 6C and 6D). Significant chemical shift differences were observed for residues in the BC and DE loops of ΔN6 upon binding to hβ_2m and mβ_2m, consistent with the head-to-head structure of both complexes. The larger number of ΔN6 residues showing chemical shift differences observed upon binding and the greater Δδ observed for the ΔN6-hβ_2m complex are consistent with the larger interface of this interaction, but could also suggest that ΔN6 responds to binding hβ_2m by undergoing conformational change. The picture that emerges, therefore, is that the promotion of hβ_2m fibril formation by ΔN6 involves weak binding that nonetheless leads to conformational changes in one or both of the interacting partners. By contrast, the ΔN6-mβ_2m complex, even though employing a similar head-to-head interaction, involves the formation of a relatively specific, tight binding, inhibitory complex with little or no effect on the conformational properties of the interacting partners.

DISCUSSION

Protein Interaction Surfaces and the Molecular Mechanism of β₂m Aggregation

Amyloid fibrils share similar structural features based upon a cross-β core, irrespective of the organism of origin, the protein involved, or the sequence of the protein precursor (Eisenberg and Jucker, 2012). Despite their similarity in structure, amyloid fibrils can be beneficial to the organism concerned, whereas for others amyloid formation is deleterious (Otzen, 2010). For each scenario, mechanisms have evolved that either facilitate assembly or protect against the accumulation of aggregation-competent proteins, depending on whether the fibrils are beneficial or not (Bucciantini et al., 2002; Otzen, 2010; Maji et al., 2009). One such example can be found in prions, proteins that possess at least one amyloid-competent conformation that is infectious by being able to transmit its structural and pathological properties onto innocuously folded prion monomers (Sindi and Serio, 2009). When prion molecules are transferred between species, they can lose their infectivity or allow propagation depending on the organism involved, establishing a so-called species barrier (Chien et al., 2003; Tessier and Lindquist, 2009; Baskakov, 2014). The precise molecular details of how and why species barriers occur between very similar proteins remain unclear. ΔN6 has been shown to possess prion-like properties in its ability to convert hβ_2m in an aggregation-prone conformation by biomolecular collision (although the protein is not infectious) (Eichner et al., 2011). Here we show that the prion-like characteristics of ΔN6 are not only limited to its ability to convert hβ_2m into an amyloid-competent conformation but also in its ability to experience species barriers (when the molecule interacts with mβ_2m, amyloid assembly is inhibited). The results show that aggregation propensity is not simply related to the kinetic and/or thermodynamic properties of the proteins involved (the least stable β_2m variant studied here [mβ_2m] inhibits assembly, whereas propagation involves interaction of ΔN6 with the most stable variant [hβ_2m]). Instead, the fate of amyloid assembly involves a fine interplay between molecular recognition and protein plasticity, which is governed by the precise location and chemical properties of the interfaces involved in the first biomolecular interaction events.

Interactions that Result in Inhibition or Promotion of Amyloid Assembly

Amyloid diseases are usually late-onset disorders, with symptoms appearing many decades into life, even for individuals carrying the most deleterious of mutations (Greenwald and Riek, 2010). Why this is the case remains unclear; possibilities include the time taken to nucleate fibril formation, and/or atrophy or overload of the proteostatic mechanisms that protect cells from protein misfolding and aggregation (Balch et al., 2008). Defining the nature of the complex network of protein-protein interactions that form in the earliest stages of amyloid assembly is of crucial importance, therefore, in our quest to understand the events that initiate protein aggregation at a molecular level. Such knowledge will also open the door to the design of inhibitors able to arrest amyloid formation by targeting specific surfaces that block the formation of fibrils and their toxic precursors, thereby halting the disease process at its outset.

Attempts to identify the intermolecular interactions that form early in amyloid assembly have remained a significant challenge as a consequence of the interactions' heterogeneity and transient nature (Cremades et al., 2012). By exploiting the power of biomolecular NMR methods and applying them to β_2m sequences from different species, we have been able to define

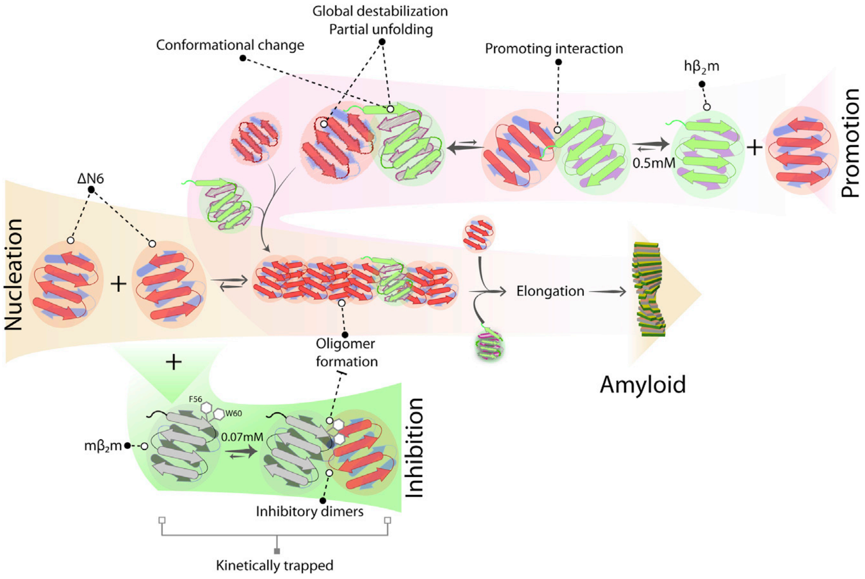

Figure 7. Model for the Nucleation, Inhibition, and Promotion of ΔN6 Fibril Formation

ΔN6 self-assembles into amyloid fibrils in a reaction that involves the formation of preamyloid oligomers (middle). $m\beta_2m$ has the ability to push the equilibrium back to monomers and trapped heterodimers, destroying or delaying the formation of the critical nucleus and kinetically inhibiting the formation of fibrils. This interaction involves the DE loops of both molecules and results in the accumulation of kinetically trapped heterodimers (bottom). $h\beta_2m$ interacts with ΔN6 in a similar head-to-head manner as $m\beta_2m$, but this interaction causes conformational changes and/or partial unfolding of $h\beta_2m$. The destabilization of the native fold generates species with increased amyloid potential, presumably facilitated by *cis-trans* isomerization of P32 in destabilized, ΔN6-bound $h\beta_2m$, explaining the mechanism by which ΔN6 is able to enhance the amyloid potential of $h\beta_2m$.

the intermolecular surfaces that determine the course of amyloid assembly. Specifically, we show that the interaction of ΔN6 with $m\beta_2m$ inhibits aggregation via trapping the amyloidogenic precursor (ΔN6) in kinetically stable dimers (K_d = 68 ± 20 μM). These involve the formation of a relatively well defined interface, stabilized by hydrophobic interactions involving the side chains of residues in the DE and BC loops of both molecules, including F56 and W60 (Figure 7, bottom). Interestingly, $m\beta_2m$ is the least stable variant of the three β_2m homologs studied here, as shown by its increased H/D exchange rates and decreased unfolding free energy relative to ΔN6 and $h\beta_2m$ (T.K.K., C. Pashley, and S.E.R., unpublished data). Thermodynamically and kinetically unstable proteins, therefore, and not only their stable counterparts (e.g., antibodies or affibodies; Dumoulin et al., 2003; Hoyer et al., 2008), can act as efficient and specific inhibitors of aggregation. Surprisingly, the amyloid-promoting association of ΔN6 with $h\beta_2m$ also involves a head-to-head interaction similar, but not identical, to that of the inhibitory complex. Consistent with this finding, the folding intermediate I_T of $h\beta_2m$ that structurally resembles ΔN6 (Eichner and Radford, 2009; Eichner et al., 2011) was recently shown to form transient oligomers during folding that are also organized in a head-to-head configuration, although the structures formed and their implications for aggregation were not described (Rennella et al., 2013).

We show here that the amyloid-promoting interaction between ΔN6 and $h\beta_2m$ is thermodynamically weaker than its inhibitory counterpart (K_d = 494 ± 180 μM) and involves multiple interaction sites that involve complementary electrostatic interactions between the interacting molecules that are not utilized in its inhibitory ΔN6-$m\beta_2m$ counterpart. These differences in the interaction interfaces result in binding-induced conformational changes in $h\beta_2m$ that are manifested by a 2- to 3-fold increase in its hydrogen exchange rates (Figure 7, top). This interaction also alters the conformation of the AB loop of $h\beta_2m$, as shown previously (Eichner et al., 2011). Accordingly, ΔN6 is able to act as protein saboteur, each molecule interacting with numerous copies of $h\beta_2m$, destabilizing the native fold of $h\beta_2m$ and allowing P32 to relax from its native *cis* isomer to its *trans* form, which then traps the protein irreversibly in an aggregation-competent state. *cis* Pro32 in $h\beta_2m$, therefore, acts as a key switch in amyloid formation. Accordingly, any event that promotes relaxation of Pro32 to the *trans* conformer (mutation, formation of ΔN6 or I_T, interaction with Cu^{2+} ions, chaperones, or proline isomerase) promotes formation of amyloid fibrils (reviewed in Eichner and Radford, 2011).

Implications for the Origins of Transmissibility in Amyloid Diseases

The results presented reveal that subtle differences in the nature of protein-protein interactions can give rise to fundamentally different outcomes of amyloid assembly that depend on the affinity of the interaction, the stability of the interacting partners, and the chemical nature of the interacting surfaces. The results have significance that extends beyond the specific case of the β_2m variants studied here. The catalytic templating model proposed to explain the conversion of the cellular human prion protein (PrP^C) to its infectious scrapie form (PrP^{SC}) is one such case (Aguzzi et al., 2008). Accordingly, mutations that have little effect on the structural and thermodynamic properties of the monomeric PrP precursors (Bae et al., 2009) could alter the surface properties of the protein, influencing the network of intermolecular interactions formed, and hence lead to increased or decreased infectivity. Other amyloid proteins that are intrinsically disordered (such as $A_{\beta40}$ and α-synuclein) are known to mutually enhance each other's aggregation (Guo et al., 2013), possibly involving a similar mechanism of binding-induced conformational change. Indeed, heteropolymerization in amyloid assembly seems to be more common than initially anticipated (Sarell et al., 2013a). As shown here, protein association, response to binding, and the effect of transient intermolecular association on the course of assembly are all interlinked. Binding, even to similar surfaces, can cause a different response on the partners

involved and thus lead to a different outcome of assembly. The HET-S/HET-s prion strains in filamentous fungi represent another example (Greenwald et al., 2010). HET-S, even though 97% identical in sequence to HET-s, does not aggregate, and can also inhibit the propagation of the prion form of HET-s by biomolecular interaction, resembling the effect of mβ_2m on ΔN6 assembly. A model for prion inhibition by HET-S has been proposed in which HET-S, although able to interact with HET-s and adopt the amyloid β-solenoid fold, is incompetent for further polymerization (Greenwald et al., 2010), further highlighting the observation that collision of similar proteins can result in different outcomes of assembly. Application of the approach taken here for β_2m to other proteins involved in human disease, including the classic examples of species barriers in PrP propagation (Baskakov, 2014), prion compatibility in yeast and other fungi (Tessier and Lindquist, 2009), and other proteins purported to be infectious (Brundin et al., 2010), will reveal the similarities and distinctions between ΔN6-induced conformational conversion and amyloid inhibition and the molecular events occurring in other systems.

As well as providing insights into the molecular origins of species barriers in amyloid formation, the results presented provide opportunities for the design of molecules to control amyloid disease by targeting intermolecular contacts in the specific surfaces involved. The design of small molecules able to disrupt protein-protein interactions and the generation of other reagents (antibodies, affibodies, or nucleic acid aptamers [Bunka et al., 2007] selected to bind to a specific surface) are exciting possibilities for future avenues of research. The complexity of amyloid assembly, especially in the cellular environment, may require multiple routes involving different strategies to delay, prevent, or revert disease to be deployed simultaneously (for example by combining interference of protein assembly with small molecules or aptamers in concert with regulation of the cellular mechanisms that recognize protein misfolding events). The ability to target the earliest biomolecular events in the aggregation cascade offers potential for a route toward amyloid therapy that will add to the arsenal of approaches currently being developed to combat these devastating disorders.

EXPERIMENTAL PROCEDURES

Protein Preparation

hβ_2m, mβ_2m, and ΔN6 (^{14}N- and ^{15}N-labeled) and their variants were expressed and purified as described (Platt et al., 2005).

Assembly of Amyloid-like Fibrils

Samples containing 0.6–60 μM protein, 10 mM sodium phosphate buffer (pH 6.2), 83.3 mM NaCl (total ionic strength 100 mM), 0.02% (w/v) sodium azide, and 10 μM ThT were incubated at 37°C in sealed 96-well plates with agitation at 600 rpm (Supplemental Experimental Procedures).

PRE Experiments

The ΔN6 variants (^{14}N-labeled) C20S, C33S, and S61C modified with MTSL (Supplemental Experimental Procedures) were mixed with ^{15}N-labeled hβ_2m or mβ_2m (60 μM, unless otherwise stated) in 10 mM sodium phosphate buffer (pH 6.2) and H_N-PRE data were measured as described in Supplemental Experimental Procedures.

Simulated Annealing Calculations

All structure calculations were performed using a torsion angle-simulated annealing protocol in XPLOR-NIH as described (Iwahara et al., 2004) (Supplemental Experimental Procedures).

K_d Measurements

Binding affinities for the complexes of mβ_2m and hβ_2m with ΔN6 were determined at pH 6.2 and 25°C by titrating 80 μM ^{15}N-labeled mβ_2m with 0–320 μM ^{14}N-labeled ΔN6 or 80 μM ^{15}N-labeled hβ_2m with 0–480 μM ^{14}N-labeled ΔN6 and measurement of the resulting chemical shift changes using ^{1}H-^{15}N HSQC spectra (Supplemental Experimental Procedures).

Hydrogen Exchange Experiments

The rate of H/D exchange of samples of ^{15}N-labeled hβ_2m or mβ_2m (80 μM) alone or mixed with ^{14}N-labeled ΔN6 (160 or 40 μM, respectively) to produce ~22% bound complexes in each case was measured at pH 6.2. Hydrogen exchange was measured using SOFAST-HMQC NMR methods as previously described (Schanda et al., 2005) (Supplemental Experimental Procedures).

Additional Procedures and Further Information

Detailed description of all other methods and protocols can be found in Supplemental Experimental Procedures.

ACCESSION NUMBERS

Assignments for the backbone atoms of mβ_2m have been deposited in the BioMagResBank under accession number 19772.

SUPPLEMENTAL INFORMATION

Supplemental Information includes Supplemental Experimental Procedures, six figures, one table, and two movies and can be found with this article online at http://dx.doi.org/10.1016/j.molcel.2014.05.026.

ACKNOWLEDGMENTS

We thank Claire Sarell and Rebecca Thompson for help with electron microscopy, James Ault for performing the mass spectrometry experiments, Clare Pashley for assistance with AUC, Nasir Khan for technical support, and David Brockwell, Anastasia Zhuravleva, and the members of the S.E.R. and Homans groups for stimulating discussions. We acknowledge the Wellcome Trust (094232) and the University of Leeds for funding the NMR instrumentation. T.K.K. and S.E.R. acknowledge funding from the European Research Council (ERC) under European Union's Seventh Framework Programme (FP7/2007-2013)/ERC grant agreement no. 322408. T.K.K. was supported by the Wellcome Trust (089311/Z/09/Z).

Received: February 7, 2014
Revised: April 22, 2014
Accepted: May 8, 2014
Published: June 26, 2014

REFERENCES

Aguzzi, A., Sigurdson, C., and Heikenwaelder, M. (2008). Molecular mechanisms of prion pathogenesis. Annu. Rev. Pathol. *3*, 11–40.

Bae, S.-H., Legname, G., Serban, A., Prusiner, S.B., Wright, P.E., and Dyson, H.J. (2009). Prion proteins with pathogenic and protective mutations show similar structure and dynamics. Biochemistry *48*, 8120–8128.

Baker, N.A., Sept, D., Joseph, S., Holst, M.J., and McCammon, J.A. (2001). Electrostatics of nanosystems: Application to microtubules and the ribosome. Proc. Natl. Acad. Sci. USA *98*, 10037–10041.

Balch, W.E., Morimoto, R.I., Dillin, A., and Kelly, J.W. (2008). Adapting proteostasis for disease intervention. Science *319*, 916–919.

Baskakov, I.V. (2014). The many shades of prion strain adaptation. Prion. Published online February 11, 2014. http://dx.doi.org/10.4161/pri.27836.

Brundin, P., Melki, R., and Kopito, R. (2010). Prion-like transmission of protein aggregates in neurodegenerative diseases. Nat. Rev. Mol. Cell Biol. *11*, 301–307.

Bucciantini, M., Giannoni, E., Chiti, F., Baroni, F., Formigli, L., Zurdo, J., Taddei, N., Ramponi, G., Dobson, C.M.C., and Stefani, M. (2002). Inherent toxicity of aggregates implies a common mechanism for protein misfolding diseases. Nature *416*, 507–511.

Bunka, D.H.J., Mantle, B.J., Morten, I.J., Tennent, G.A., Radford, S.E., and Stockley, P.G. (2007). Production and characterization of RNA aptamers specific for amyloid fibril epitopes. J. Biol. Chem. *282*, 34500–34509.

Calabrese, M.F., and Miranker, A.D. (2009). Metal binding sheds light on mechanisms of amyloid assembly. Prion *3*, 1–4.

Campioni, S., Mannini, B., Zampagni, M., Pensalfini, A., Parrini, C., Evangelisti, E., Relini, A., Stefani, M., Dobson, C.M., Cecchi, C., and Chiti, F. (2010). A causative link between the structure of aberrant protein oligomers and their toxicity. Nat. Chem. Biol. *6*, 140–147.

Chien, P., and Weissman, J.S. (2001). Conformational diversity in a yeast prion dictates its seeding specificity. Nature *410*, 223–227.

Chien, P., DePace, A.H., Collins, S.R., and Weissman, J.S. (2003). Generation of prion transmission barriers by mutational control of amyloid conformations. Nature *424*, 948–951.

Chien, P., Weissman, J.S., and DePace, A.H. (2004). Emerging principles of conformation-based prion inheritance. Annu. Rev. Biochem. *73*, 617–656.

Chiti, F., and Dobson, C.M. (2009). Amyloid formation by globular proteins under native conditions. Nat. Chem. Biol. *5*, 15–22.

Clore, G.M., and Iwahara, J. (2009). Theory, practice, and applications of paramagnetic relaxation enhancement for the characterization of transient low-population states of biological macromolecules and their complexes. Chem. Rev. *109*, 4108–4139.

Cremades, N., Cohen, S.I.A., Deas, E., Abramov, A.Y., Chen, A.Y., Orte, A., Sandal, M., Clarke, R.W., Dunne, P., Aprile, F.A., et al. (2012). Direct observation of the interconversion of normal and toxic forms of α-synuclein. Cell *149*, 1048–1059.

Dumoulin, M., Last, A.M., Desmyter, A., Decanniere, K., Canet, D., Larsson, G., Spencer, A., Archer, D.B., Sasse, J., Muyldermans, S., et al. (2003). A camelid antibody fragment inhibits the formation of amyloid fibrils by human lysozyme. Nature *424*, 783–788.

Eichner, T., and Radford, S.E. (2009). A generic mechanism of β_2-microglobulin amyloid assembly at neutral pH involving a specific proline switch. J. Mol. Biol. *386*, 1312–1326.

Eichner, T., and Radford, S.E. (2011). Understanding the complex mechanisms of β_2-microglobulin amyloid assembly. FEBS J. *278*, 3868–3883.

Eichner, T., Kalverda, A.P., Thompson, G.S., Homans, S.W., and Radford, S.E. (2011). Conformational conversion during amyloid formation at atomic resolution. Mol. Cell *41*, 161–172.

Eisenberg, D., and Jucker, M. (2012). The amyloid state of proteins in human diseases. Cell *148*, 1188–1203.

Esposito, G., Michelutti, R., Verdone, G., Viglino, P., Hernández, H., Robinson, C.V., Amoresano, A., Dal Piaz, F., Monti, M., Pucci, P., et al. (2000). Removal of the N-terminal hexapeptide from human β_2-microglobulin facilitates protein aggregation and fibril formation. Protein Sci. *9*, 831–845.

Fitzpatrick, A.W.P., Debelouchina, G.T., Bayro, M.J., Clare, D.K., Caporini, M.A., Bajaj, V.S., Jaroniec, C.P., Wang, L., Ladizhansky, V., Müller, S.A., et al. (2013). Atomic structure and hierarchical assembly of a cross-β amyloid fibril. Proc. Natl. Acad. Sci. USA *110*, 5468–5473.

Gejyo, F., Yamada, T., Odani, S., Nakagawa, Y., Arakawa, M., Kunitomo, T., Kataoka, H., Suzuki, M., Hirasawa, Y., Shirahama, T., et al. (1985). A new form of amyloid protein associated with chronic hemodialysis was identified as β_2-microglobulin. Biochem. Biophys. Res. Commun. *129*, 701–706.

Giasson, B.I., Forman, M.S., Higuchi, M., Golbe, L.I., Graves, C.L., Kotzbauer, P.T., Trojanowski, J.Q., and Lee, V.M.Y. (2003). Initiation and synergistic fibrillization of tau and α-synuclein. Science *300*, 636–640.

Greenwald, J., and Riek, R. (2010). Biology of amyloid: structure, function, and regulation. Structure *18*, 1244–1260.

Greenwald, J., Buhtz, C., Ritter, C., Kwiatkowski, W., Choe, S., Maddelein, M.-L., Ness, F., Cescau, S., Soragni, A., and Leitz, D. (2010). The mechanism of prion inhibition by HET-S. Mol. Cell *38*, 889–899.

Guo, J.L., Covell, D.J., Daniels, J.P., Iba, M., Stieber, A., Zhang, B., Riddle, D.M., Kwong, L.K., Xu, Y., Trojanowski, J.Q., and Lee, V.M. (2013). Distinct α-synuclein strains differentially promote tau inclusions in neurons. Cell *154*, 103–117.

Hodkinson, J.P., Jahn, T.R., Radford, S.E., and Ashcroft, A.E. (2009). HDX-ESI-MS reveals enhanced conformational dynamics of the amyloidogenic protein β_2-microglobulin upon release from the MHC-1. J. Am. Soc. Mass Spectrom. *20*, 278–286.

Hoyer, W., Grönwall, C., Jonsson, A., Ståhl, S., and Härd, T. (2008). Stabilization of a β-hairpin in monomeric Alzheimer's amyloid-β peptide inhibits amyloid formation. Proc. Natl. Acad. Sci. USA *105*, 5099–5104.

Ivanova, M.I., Sawaya, M.R., Gingery, M., Attinger, A., and Eisenberg, D. (2004). An amyloid-forming segment of β_2-microglobulin suggests a molecular model for the fibril. Proc. Natl. Acad. Sci. USA *101*, 10584–10589.

Iwahara, J., Schwieters, C.D., and Clore, G.M. (2004). Ensemble approach for NMR structure refinement against ^{1}H paramagnetic relaxation enhancement data arising from a flexible paramagnetic group attached to a macromolecule. J. Am. Chem. Soc. *126*, 5879–5896.

Jahn, T.R., Parker, M.J., Homans, S.W., and Radford, S.E. (2006). Amyloid formation under physiological conditions proceeds via a native-like folding intermediate. Nat. Struct. Mol. Biol. *13*, 195–201.

Maji, S.K., Perrin, M.H., Sawaya, M.R., Jessberger, S., Vadodaria, K., Rissman, R.A., Singru, P.S., Nilsson, K.P., Simon, R., Schubert, D., et al. (2009). Functional amyloids as natural storage of peptide hormones in pituitary secretory granules. Science *325*, 328–332.

Middleton, C.T., Marek, P., Cao, P., Chiu, C.-C., Singh, S., Woys, A.M., de Pablo, J.J., Raleigh, D.P., and Zanni, M.T. (2012). Two-dimensional infrared spectroscopy reveals the complex behaviour of an amyloid fibril inhibitor. Nat. Chem. *4*, 355–360.

Neudecker, P., Robustelli, P., Cavalli, A., Walsh, P., Lundström, P., Zarrine-Afsar, A., Sharpe, S., Vendruscolo, M., and Kay, L.E. (2012). Structure of an intermediate state in protein folding and aggregation. Science *336*, 362–366.

Otzen, D. (2010). Functional amyloid: turning swords into plowshares. Prion *4*, 256–264.

Platt, G.W., McParland, V.J., Kalverda, A.P., Homans, S.W., and Radford, S.E. (2005). Dynamics in the unfolded state of β_2-microglobulin studied by NMR. J. Mol. Biol. *346*, 279–294.

Rennella, E., Cutuil, T., Schanda, P., Ayala, I., Gabel, F., Forge, V., Corazza, A., Esposito, G., and Brutscher, B. (2013). Oligomeric states along the folding pathways of β_2-microglobulin: kinetics, thermodynamics, and structure. J. Mol. Biol. *425*, 2722–2736.

Rudolph, M.G., Shen, L.Q., Lamontagne, S.A., Luz, J.G., Delaney, J.R., Ge, Q., Cho, B.K., Palliser, D., McKinley, C.A., Chen, J., et al. (2004). A peptide that antagonizes TCR-mediated reactions with both syngeneic and allogeneic agonists: functional and structural aspects. J. Immunol. *172*, 2994–3002.

Sakata, M., Chatani, E., Kameda, A., Sakurai, K., Naiki, H., and Goto, Y. (2008). Kinetic coupling of folding and prolyl isomerization of β_2-microglobulin studied by mutational analysis. J. Mol. Biol. *382*, 1242–1255.

Sarell, C.J., Stockley, P.G., and Radford, S.E. (2013a). Assessing the causes and consequences of co-polymerization in amyloid formation. Prion *7*, 359–368.

Sarell, C.J., Woods, L.A., Su, Y., Debelouchina, G.T., Ashcroft, A.E., Griffin, R.G., Stockley, P.G., and Radford, S.E. (2013b). Expanding the repertoire of amyloid polymorphs by co-polymerization of related protein precursors. J. Biol. Chem. *288*, 7327–7337.

Schanda, P., Kupče, E., and Brutscher, B. (2005). SOFAST-HMQC experiments for recording two-dimensional heteronuclear correlation spectra of proteins within a few seconds. J. Biomol. NMR *33*, 199–211.

Sindi, S.S., and Serio, T.R. (2009). Prion dynamics and the quest for the genetic determinant in protein-only inheritance. Curr. Opin. Microbiol. *12*, 623–630.

Smith, D.P., Radford, S.E., and Ashcroft, A.E. (2010). Elongated oligomers in β_2-microglobulin amyloid assembly revealed by ion mobility spectrometry-mass spectrometry. Proc. Natl. Acad. Sci. USA *107*, 6794–6798.

Tang, C., Iwahara, J., and Clore, G.M. (2006). Visualization of transient encounter complexes in protein-protein association. Nature *444*, 383–386.

Tang, C., Ghirlando, R., and Clore, G.M. (2008). Visualization of transient ultraweak protein self-association in solution using paramagnetic relaxation enhancement. J. Am. Chem. Soc. *130*, 4048–4056.

Tessier, P.M., and Lindquist, S. (2009). Unraveling infectious structures, strain variants and species barriers for the yeast prion [PSI^+]. Nat. Struct. Mol. Biol. *16*, 598–605.

Xue, W.F., Homans, S.W., and Radford, S.E. (2008). Systematic analysis of nucleation-dependent polymerization reveals new insights into the mechanism of amyloid self-assembly. Proc. Natl. Acad. Sci. USA *105*, 8926–8931.

Zhuravleva, A., and Gierasch, L.M. (2011). Allosteric signal transmission in the nucleotide-binding domain of 70-kDa heat shock protein (Hsp70) molecular chaperones. Proc. Natl. Acad. Sci. USA *108*, 6987–6992.

Molecular Cell, Volume *55*

Supplemental Information

Visualization of Transient Protein-Protein Interactions that Promote or Inhibit Amyloid Assembly

Theodoros K. Karamanos, Arnout P. Kalverda, Gary S. Thompson, and Sheena E. Radford

Supplemental Figures and Legends

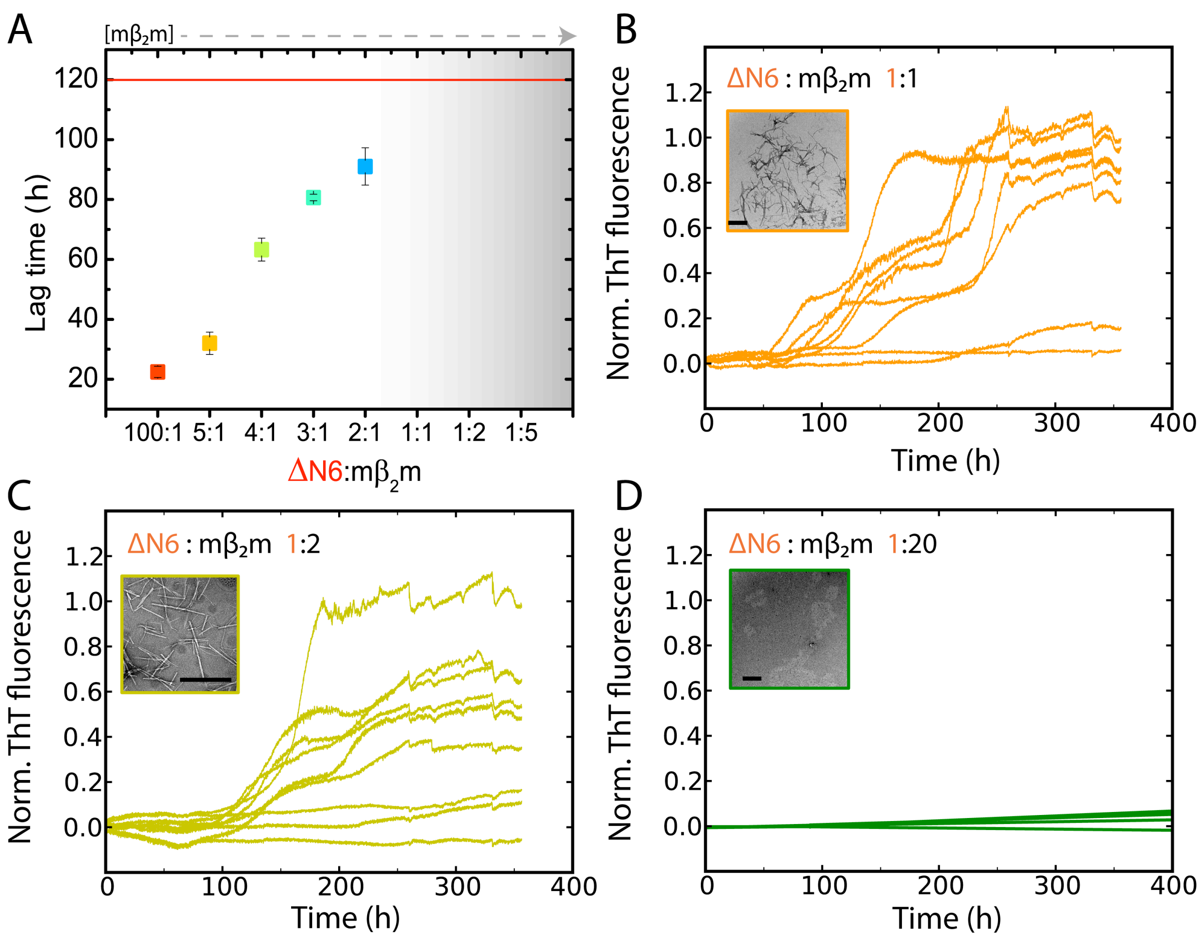

Figure S1 - related to Figure 2: Kinetic inhibition of ΔN6 amyloid formation by mβ_2m. (A) Plot of the average lag time of fibril formation for different molar ratios of ΔN6:mβ_2m. The grey-shaded area represents experiments where the lag time could not be estimated because the protein mixtures did not show an increase in ThT fluorescence by the end of the experiment shown in Figure 2A (120h-red line). Error bars represent the standard error of the mean. (B) Kinetics of amyloid assembly of mixtures of ΔN6:mβ_2m: 30µM:30µM, (C) 20µM:40µM and (D) 10µM:200µM over timescales up to 400h monitored using ThT fluorescence. Negative stain EM images of the fibrils formed at the end of each reaction are inset (scale bar = 500nm).

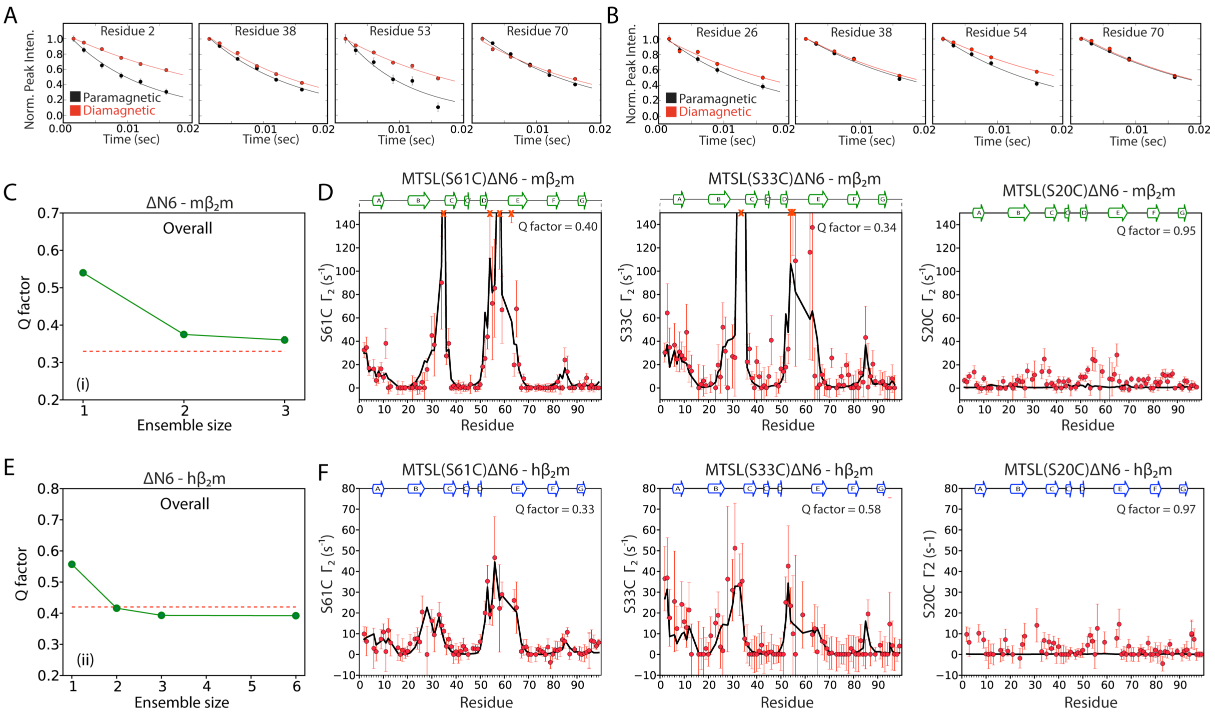

Figure S2 – related to Figure 4. Agreement between the experimental and back-calculated intermolecular PRE data for the ΔN6-mβ_2m and ΔN6-hβ_2m interactions. Intermolecular PRE profiles for the interaction between ^{14}N-ΔN6 spin labeled at position 61 and ^{15}N-labeled mβ_2m (A) or ^{15}N-labeled hβ_2m (B). (C) Plots of the calculated Q factor versus the number of ensemble members for the interaction of ΔN6 with mβ_2m. Dashed red lines represent the best Q factor possible for each dataset, if the quality of the fit is comparable to the error of the experimental data and is calculated as described in (Tang et al., 2008). (D) Experimental and calculated PRE data for the ΔN6-mβ_2m interaction when the spin label is attached at position 61 (left), 33 (middle) or 20 (right). Predicted PRE rates for an ensemble size of 2 (N=2) are shown in black lines while red dots denote the experimentally measured PRE rates. Data arising from the spin label on position 20 were not used in the fitting. The calculated Γ_2 rates shown represent the average values (per residue), back-calculated from 50 independent calculations (50x2 structures). All ensemble members were equally weighted during the calculations. (E) As in (C) and (F) as in (D) but for the association of ΔN6 with hβ_2m.

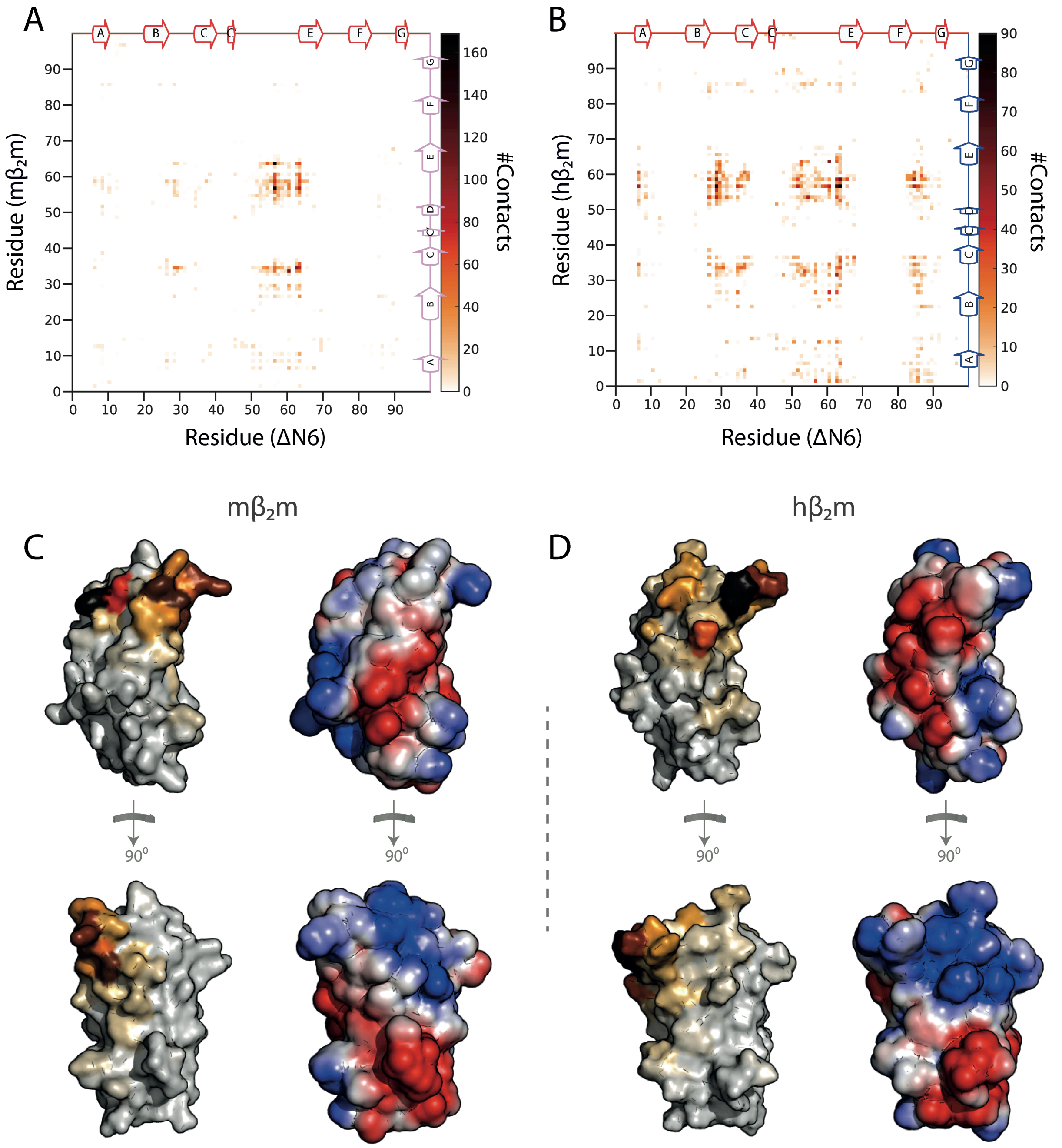

Figure S3 – related to Figure 4. Analysis of the ΔN6-m$β_2$m and the ΔN6-h$β_2$m interaction ensembles. (A) Contact map for the 50 best scoring ensembles for the ΔN6-m$β_2$m or (B) the ΔN6-h$β_2$m interaction. Every non-hydrogen atom with an intermolecular distance less than 4Å to any other (non-hydrogen) atom is identified as a contact. The number of contacts for atoms of each residue is color-coded as shown in the color-bar. (C) Structures m$β_2$m colored according to the number of intermolecular contacts, using the same color scale as in (A) (left). A surface representation of the protein, colored according to its electrostatic potential (red

negative, blue positive, ±2k_BT) is shown in the right. (D) As in (C) but for hβ_2m. The pose of the proteins is the same in both cases, with the BC, DE and FG loops at the top of the molecule.

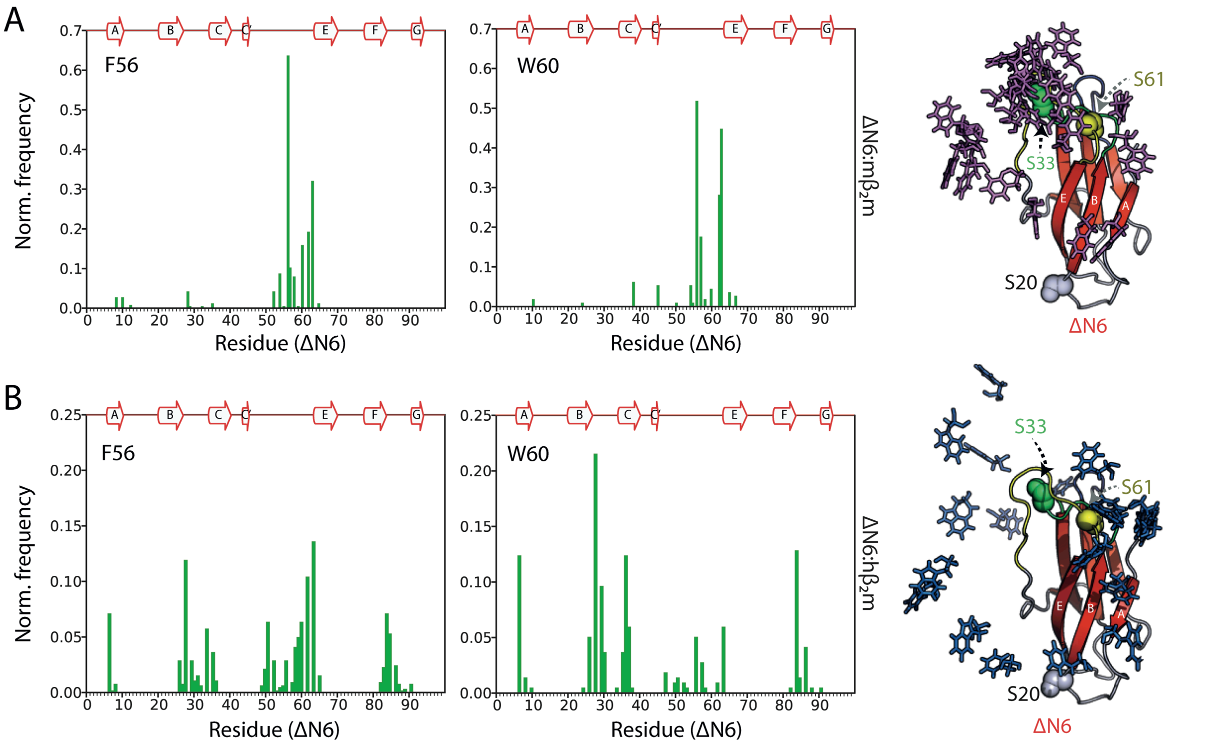

Figure S4 – related to Figure 5. The roles of F56 and W60 in the interface of different complexes. (A) Normalized frequency histograms of the number of contacts that F56 (left) or W60 (middle) of mβ_2m make with residues of ΔN6 in the ΔN6-mβ_2m complex. The 50 best-scoring ensembles (N=2, 50x2 structures) were analysed. These histograms essentially represent horizontal slices of the heat maps shown in Figure S3. The rightmost panel shows the position of F56 and W60 of mβ_2m (sticks) in the top 10 ensembles. ΔN6 is shown as red cartoon and the positions of the spin labels (S20, S33 and S61) are highlighted in spheres. (B) As in (A) but for the ΔN6-hβ_2m interaction. The distribution of F56 and W60 in the ΔN6-hβ_2m complex is much more diverse in comparison its ΔN6-mβ_2m counterpart. As a consequence, whilst the F56E/W60E mβ_2m mutant prevents the association of ΔN6 with mβ_2m, a more detailed mutational analysis in the interface of the ΔN6-hβ_2m complex is required to unpick the roles of specific residues in the course of assembly (Figure S4B).

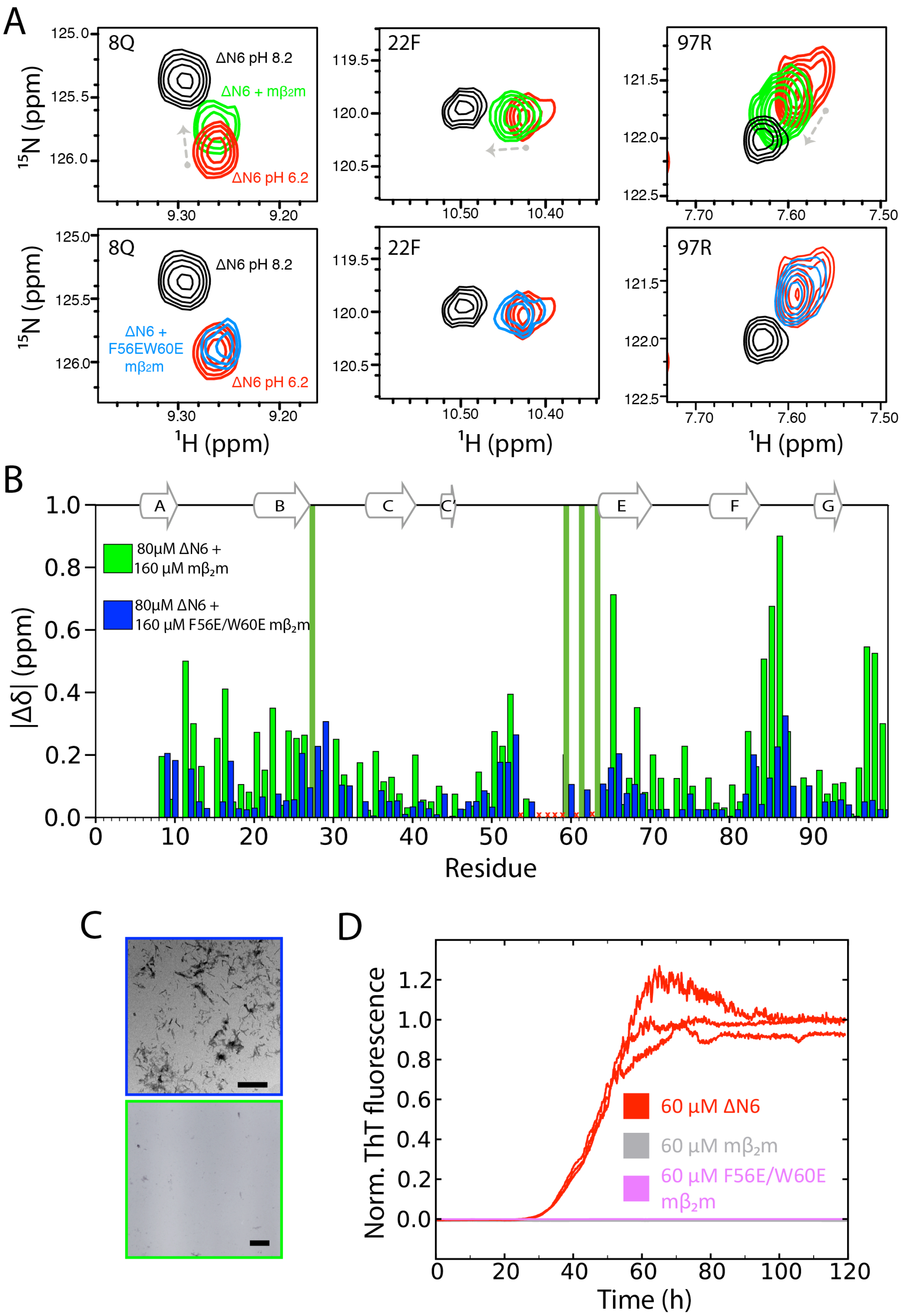

Figure S5 –related to Figure 5. Residual interactions between F56E/W60E mβ_2m and ΔN6 are not sufficient to inhibit fibrilation. (A) Additional examples of resonances of ^{15}N-labeled

ΔN6 (80μM, red) that show chemical shift changes upon the addition of ^{14}N-labeled mβ_2m (green), but not its F56E/W60E variant (160μM, blue) at pH6.2, 25°C. (B) Changes in the chemical shifts of 80μM ^{15}N-labelled ΔN6 upon addition of 160μM ^{14}N-labelled mβ_2m (green) or F56E/W60E mβ_2m (blue) (pH 6.2, 25ºC). Residues that are broadened beyond detection because of exchange line broadening in the spectrum of ΔN6:mβ_2m have an arbitrary value of 1. The small chemical shift differences observed in the F56E/W60E mβ_2m:ΔN6 sample suggest a residual interaction with increased K_d. (C) Negative stain electron micrograph of 20μM ΔN6 mixed with two molar equivalents of F56E/W60E mβ_2m (top) or wild-type mβ_2m (bottom). Bar represents 500nm. (D) Aggregation kinetics of ΔN6, F56E/W60E mβ_2m or wild-type mβ_2m (60μM each alone) followed by ThT fluorescence. Three example traces of each protein variant are shown.

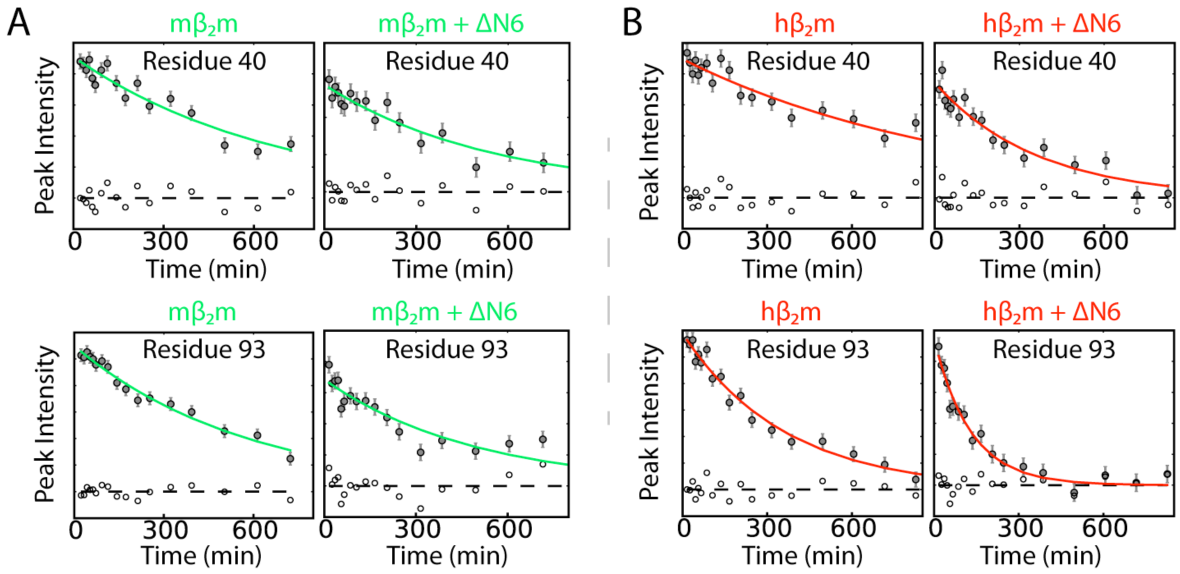

Figure S6 – related to Figure 6. H/D exchange rates of mβ_2m and hβ_2m upon interaction with ΔN6. (A) Plots of peak intensity versus time after the initiation of H/D exchange for example residues of ^{15}N-labeled mβ_2m alone (80µM, left column) or in the presence of 40µM ^{14}N-labeled ΔN6 (right column). Solid grey dots represent the raw data and solid lines show fits to single exponentials. Error bars were calculated from the noise level of the experiment. Open symbols show the residuals of the fits. (B) as in (A) but for ^{15}N-labeled hβ_2m alone (80µM) or in the presence of 160µM ^{14}N-labeled ΔN6.

Supplemental Movies

Movie S1, related to Figure 4. The mβ_2m-ΔN6 and hβ_2m-ΔN6 complexes involve different subunit orientations of a common head-to-head dimer. Movie animation of the structural ensembles shown in Figures 4C and 4D. ΔN6 is shown as cartoon representation with its BC loop highlighted in green, the DE loop in yellow and the FG loop in blue. The ensemble of mβ_2m molecules around ΔN6 is shown as a pink surface on the left hand side, while the hβ_2m ensemble is shown as a blue surface in the right had side.

Movie S2, related to Figure 4. The mβ_2m-ΔN6 and hβ_2m-ΔN6 complexes show different chemical properties in the interface. Mβ_2m (left hand side) and hβ_2m (right hand side) are shown as a surface representation coloured according to their electrostatic potential ($\pm 2k_BT$), with the BC, DE and FG loops on the top. The ensemble of ΔN6 molecules around mβ_2m and/or hβ_2m is shown as green and yellow mesh respectively. This representation is essentially the complementary picture of the ensembles shown in Figures 4C and 4D (where mβ_2m and/or hβ_2m were shown as weighted atomic probability density maps). Note the high correlation between the distribution of ΔN6 molecules around mβ_2m with the hydrophobic surface of the latter. By contrast, part of the ΔN6 density map locates opposite the negatively charged part of the BC loop of hβ_2m. The electrostatic surface potential was calculated using APBS (Baker et al., 2001) and movies were rendered in Pymol (Schrodinger, LLC, 2010).

Supplemental Tables

Table S1 – related to Figure 4. Analysis of the interfaces of different β_2m complexes. The buried surface area is calculated as the sum for the two subunits for each complex and is measured using XPLOR-NIH (Schwieters et al., 2003). Interface residues were identified as NACCESS (Hubbard and Thornton, 1993). Errors represent the standard deviation.

	**ΔN6 - mβ_2m complex**	**ΔN6 - hβ_2m complex**
Density volume (Å^3)	7,157	13,670
Buried surface area (Å^2)[1]	1481±290	1359±357
% Hydrophobic residues in the interface	48.6±14.5	43.81±12.6
% Charged residues in the interface	20.4±12.0	31.0±14.8

[1] Within each ensemble member

Supplemental Experimental Procedures

Protein preparation

Mβ_2m and F56A/W60A mβ_2m were purified using the protocol described in (Eichner et al., 2011), but the refolding buffer was adjusted to pH 8.5 and gel filtration was carried out in 10mM sodium phosphate, pH 8.2. S20C, S33C and S61C mutants of ΔN6 were refolded in 10mM TrisHCl buffer containing 0.64M L-arginine and/or 2mM reduced glutathione and/or 0.2mM oxidized glutathione before purification as in (Ladner et al., 2010).

Assembly of amyloid-like fibrils

Each experiment was repeated over at least 10 replicate samples and the median and/or mean lag time and standard error of the mean (SEM) were determined. Analysis of the soluble and insoluble material in each reaction was carried out at the end of fibril growth (120h or 350h) by collecting insoluble material by centrifugation (15.000*g*, 20min) and analysis by SDS-PAGE or ESI-MS (the latter subsequent to depolymerization by incubation for 10h in 100% *(v/v)* 1,1,1,3,3,3-hexafluoro-2-isopropanol (HFIP) (Sarell et al., 2013). Fibril morphology was analyzed using negative stain EM.

Negative-stain EM

Carbon coated copper grids were prepared by the application of a thin layer of formvar with an overlay of thin carbon. Samples were centrifuged (14,000*g*, 10min) and the pellets were resuspended in fresh 10mM sodium phosphate buffer, pH 6.2, diluted to a final protein concentration of 12μM with deionized water and then applied to the grid in a drop-wise fashion. The grid was then carefully dried with filter paper before it was negatively stained by

the addition of 18 μl of 2% (*w/v*) uranyl acetate. Micrographs were recorded on a Philips CM10 or a JEOL JEM-1400 electron microscope.

Analytical ultracentrifugation

For sedimentation velocity experiments, a total volume of 450μl sample in 10mM sodium phosphate buffer, pH 6.2, 83.3mM NaCl was inserted in standard double-sector Epon centerpieces equipped with sapphire windows, inserted in an An60 Ti four-cell rotor. Sample concentrations included 60μM or 120μM (ΔN6 alone), 60μM ΔN6 mixed with 60μM mβ_2m or 60μM F56E/W60E mβ_2m at pH 6.2. Absorbance data at 280nm were acquired at a rotor speed of 50,000rpm at 25°C. Data were analyzed using the c(s) continuous distribution of the Lamm equations with the software SEDFIT (Brown and Schuck, 2006).

$$D(s) = \frac{\sqrt{2}}{18\pi} kTs^{-1/2}(\eta(f/f_0)_w)^{-3/2}((1-\bar{v}\rho)/\bar{v})^{1/2} \quad ,$$

where D(s) is the diffusion coefficient, k Boltzmann's constant, T the temperature in K, s the sedimentation coefficient, f is the frictional coefficient, f_0 the frictional coefficient of a compact smooth sphere, η the solvent viscosity, ρ represents the solvent density and $\bar{v}$ the partial specific volume.

Backbone assignments of mβ_2m

Backbone assignments for mβ_2m were obtained using triple resonance NMR techniques (HNCA, HNCO, HNCACB, HNCACO, HN(CO)CACB) and samples containing 500-750μM uniformly labeled $^{13}C/^{15}N$ mβ_2m in 10mM sodium phosphate buffer, pH 6.2, 83.3mM NaCl. Assignments are deposited in BMRB (19772).

PRE experiments

The ΔN6 variants (^{14}N-labeled) S20C, S33C and S61C (1-2mg/mL) were incubated with 5mM DTT for 20min, and then labeled immediately with MTSL by incubation with a 40-fold molar excess of the spin label for 4h in 25mM sodium phosphate buffer, pH 7.0, 1mM EDTA at room temperature. Excess spin label was removed by gel filtration (PD10 column, GE Healthcare). Spin-labeled ΔN6 was used directly or stored at -80°C. In all cases 100% labeling resulted at a single site as revealed by ESI-MS (not shown). For each PRE experiment MTSL-labeled ^{14}N-ΔN6 (10-60μM) was mixed with ^{15}N-labeled hβ_2m or mβ_2m (60-150μM) and the difference of the proton R_2 rates between oxidized and reduced (by addition of 1mM ascorbic acid) MTSL-labeled ^{14}N-ΔN6 was measured. Data were recorded at 25°C using a ^{1}H-^{15}N correlation based pulse sequence with 5-6 time-points (0.0016-0.016s) and at least 32 scans per incremental delay, utilizing a Varian-Inova 750MHz spectrometer equipped with a cryogenic probe. R_2 rates were extracted by fitting the relaxation data to single exponentials using in-house scripts. The H_N-Γ_2 rate was then calculated as the difference between the R_2 rate in the paramagnetic versus the diamagnetic sample:

$$\Gamma_2 = R_{2,para} - R_{2,dia} \quad .$$

Errors were calculated based on the noise of the experiment. The small PRE signal observed when ΔN6 is modified with MTSL at position 20 can be attributed to non-specific binding of the spin label itself, since addition of free MTSL results in a similar PRE profile. Thus, data arising from spin-labeled ΔN6 at position 20 were not included in quantitative analysis of the PRE experiments.

Simulated annealing calculations

Simulated annealing calculations were carried out in XPLOR-NIH (Schwieters et al., 2003). To account for the flexibility of the MTSL side chain, the paramagnetic group was

represented as a 5 membered ensemble. The agreement between the experimental and the back-calculated data is described by the PRE Q factor (Tang et al., 2006) defined as:

$$Q = \left[\sum i(\Gamma_{2,i}^{obs} - \Gamma_{2,i}^{cal})^2 / \sum i(\Gamma_{2,i}^{obs})^2\right]^{1/2} ,$$

where $\Gamma_{2,i}{}^{obs}$ is the observed Γ_2 value for residue i and $\Gamma_{2,i}{}^{calc}$ is the calculated Γ_2 value. All calculations were started from randomized starting positions.

The computational strategy employed included two PRE potential terms (arising from S61C-ΔN6 and S33C-ΔN6) and classic geometry restraints to restrict deviation from bond lengths, angles and dihedrals. To generate a dataset suitable for this analysis, PREs arising from position 61 and 33 for the ΔN6-hβ_2m interaction were (each) measured in two independent experiments and the average PRE value for each residue was used for fitting. For the ΔN6-mβ_2m interaction, resonances in the BC and DE loop are not visible in the spectrum of the oxidized sample when the spin label is attached at positions 33 or 61 when the proteins are mixed in a 1:1 molar ratio. To obtain an estimate for the Γ_2 rate for these residues, the PRE experiments (using spin-labeled ΔN6 at positions 33 or 61) were repeated at different protein concentrations to: 1) improve the signal-to-noise ratio and 2) reduce the concentration of the bound complex so that a more accurate Γ_2 rate can be measured. PREs were then extrapolated to their values for a 1:1 complex using the measured K_d and the resulting dataset was used for quantitative analysis of the structural properties of the complex. Resonances for which an estimation of the R_2 rate in the presence of oxidized spin label was not possible, were incorporated in the protocol as nOe-type of restraints with an upper bound of 11.5Å and a lower bound of 9Å. Additionally, chemical shift perturbations observed upon binding were incorporated as sparse, highly ambiguous intermolecular distance restraints as described in (Clore and Schwieters, 2003). As chemical shifts can be influenced by numerous factors upon protein-protein interaction, the treatment of the derived data undertaken here results in a loose

potential term that is unlikely to bias the structure calculation. Finally, the protocol also included a weak radius of gyration restraint (R_{gyr}) calculated as $2.2N^{0.38}$, where N is the number of atoms in the complex. R_{gyr} is required in order to prevent bias towards more extended structures and tends to underestimate the true value of the radius of gyration (Kuszewski et al., 1999).

The aforementioned potential terms were used in a rigid-body energy minimization/ simulated annealing in torsion angle space protocol to minimize the difference between the observed and calculated Γ_2 rates, starting from random orientations. The first step in the structure calculation consisted of 5000 steps of energy minimization against only the sparse chemical shift restraints, followed by simulated annealing dynamics with all the potential terms active, where the temperature is slowly decreased (3000-25K) over 4fs. During the hot phase (T=3000K) the PRE and nOe terms were underweighted to allow the proteins to sample a large conformational space and they were geometrically increased during the cooling phase. Proteins were treated as rigid bodies until the initiation of the cooling phase, where side chains were allowed to float (semi-rigid body calculation). The final step included torsion angle minimization using all potential terms. Ensemble calculations where the interacting species are represented as multiple states (N>1) were carried out as before but in this case the Γ_2^{cal} is calculated as the average value between the conformers. The population of each conformer was set by specifying its weight in the calculation. For ensemble calculations the Q factor is calculated by averaging the predicted PRE value over all ensemble members corrected by their weight (the Q factor of the ensemble of ensembles).

In the case of the ΔN6-mβ_2m interaction when N=1, 7 out of 10 lowest energy structures share a backbone RMSD for the mβ_2m subunit of 4.4Å, suggesting that the complex shown in

Figure 5A represents the main associating species in solution. On the other hand, a single conformer representation (N=1) for the ΔN6-hβ_2m association yields a hβ_2m subunit RMSD of 20.5Å for the 10 lowest energy structures, an observation that also supports the larger conformational ensemble between ΔN6 and hβ_2m shown in Figure 4D. Buried surface area calculations were carried out in XPLOR-NIH (Schwieters et al., 2003).

Fitting K_d values

The total chemical shift differences as each ^{15}N-labeled protein was titrated with ^{14}N-labeled ΔN6 (pH 6.2, 25ºC) was calculated using the function:

$$\Delta\delta_{tot} = \sqrt{(5 * \delta^1 H)^2 + (\delta^{15} N)^2}\,.$$

Residues for which the difference in chemical shift upon binding was ≥2 standard deviations from the mean were considered significant. These were used globally to extract the K_d using the function:

$$\Delta\delta = \Delta\delta_{\max} \frac{[L_T] + [U_T] + K_d - \sqrt{([L_T] + [U_T] + K_d)^2 - 4[L_T][U_T]}}{2[L_T]}\,,$$

where $[L_T]$, $[U_T]$ are the total concentrations of the labeled and unlabeled protein added respectively, and $\Delta\delta_{max}$ represents the highest value of the chemical shift difference upon titration. A total of 10 and 8 residues were fitted for the ΔN6:mβ_2m and the ΔN6:hβ_2m interactions respectively. Four representative examples are shown in Figures 3C and 3D for clarity. Errors on the measured peak positions were calculated as the standard deviation of the mean for residues that show insignificant chemical shift changes. K_ds were extracted by Monte Carlo analysis with 1000 steps performed using in-house scripts.

Hydrogen exchange measurements

Samples for H/D exchange NMR were made in 10mM sodium phosphate pH 6.2 buffer and

freezed-dried. On the day of the experiment, samples were dissolved in 100% (*v/v*) D_2O containing 83.3mM NaCl and hydrogen exchange was measured using SOFAST-HMQC NMR methods (Schanda et al., 2005) utilizing a 750MHz Varian Inova spectrometer (Agilent) equipped with a cryogenic probe. The dead time of the experiment was 5-10min and each spectrum was acquired for 10-15min. H/D exchange rates for mβ_2m/hβ_2m were measured at 25ºC and 37ºC respectively. Rates were extracted by fitting single exponentials (Figure S6).

Supplemental References

Baker, N.A., Sept, D., Joseph, S., Holst, M.J., and McCammon, J.A. (2001). Electrostatics of nanosystems: Application to microtubules and the ribosome. Proc. Natl. Acad. Sci. USA *98*, 10037–10041.

Brown, P.H., and Schuck, P. (2006). Macromolecular size-and-shape distributions by sedimentation velocity analytical ultracentrifugation. Biophys. J. *90*, 4651–4661.

Clore, G.M., and Schwieters, C.D. (2003). Docking of protein–protein complexes on the basis of highly ambiguous intermolecular distance restraints derived from ^{1}H / ^{15}N chemical shift mapping and backbone ^{1}H - ^{15}N residual dipolar couplings using conjoined rigid body/torsion angle dynamics. J. Am. Chem. Soc. *125*, 2902–2912.

Eichner, T., Kalverda, A.P., Thompson, G.S., Homans, S.W., and Radford, S.E. (2011). Conformational conversion during amyloid formation at atomic resolution. Mol. Cell *41*, 161–172.

Hubbard, S.J., and Thornton, J.M. (1993). "NACCESS," computer program.

Kuszewski, J., Gronenborn, A., and Clore, M. (1999). Improving the packing and accuracy of NMR structures with a pseudopotential for the radius of gyration. J. Am. Chem. Soc. *121*, 2337–2338.

Ladner, C.L., Chen, M., Smith, D.P., Platt, G.W., Radford, S.E., and Langen, R. (2010). Stacked sets of parallel, in-register β-strands of β_2-microglobulin in amyloid fibrils revealed by site-directed spin labeling and chemical labeling. J. Biol. Chem. *285*, 17137–17147.

Sarell, C.J., Woods, L.A., Su, Y., Debelouchina, G.T., Ashcroft, A.E., Griffin, R.G., Stockley, P.G., and Radford, S.E. (2013). Expanding the repertoire of amyloid polymorphs by co-polymerization of related protein precursors. J. Biol. Chem. *288*, 7327–7337.

Schanda, P., Kupče, Ē., and Brutscher, B. (2005). SOFAST-HMQC experiments for recording two-dimensional heteronuclear correlation spectra of proteins within a few seconds. J. Biomol. NMR *33*, 199–211.

Schrodinger, LLC (2010). The PyMOL Molecular Graphics System, Version~1.7

Schwieters, C.D., Kuszewski, J.J., Tjandra, N., and Clore, G.M. (2003). The Xplor-NIH NMR molecular structure determination package. J. Magn. Reson. *160*, 65–73.

Tang, C., Ghirlando, R., and Clore, G.M. (2008). Visualization of transient ultra-weak protein self-association in solution using paramagnetic relaxation enhancement. J. Am. Chem. Soc. *130*, 4048–4056.

Tang, C., Iwahara, J., and Clore, G.M. (2006). Visualization of transient encounter complexes in protein-protein association. Nature *444*, 383–386.
